# Supplementary material for: Can we design the next generation of digital health communication programs by leveraging the power of artificial intelligence to segment target audiences, bolster impact and deliver differentiated services? A machine learning analysis of survey data from rural India
Source: BMJ Open. 2023 Mar 17;13(3):e063354. doi: 10.1136/bmjopen-2022-063354 (PMC10030469; doi:10.1136/bmjopen-2022-063354)

Supplementary Table1. Study sample characteristics (variables used as starting point for couple's survey data)

	Women's survey		Men's survey	
**Variables**	N	%	N	%
**Education**				
0-5 years	610	18	586	17
>5 years	2874	82	2898	83
**District**				
Hoshangabad	345	10	345	10
Mandsaur	676	19	676	19
Rajgarh	791	23	791	23
Rewa	1672	48	1672	48
**Ethnicity/Caste**				
General	780	22	698	20
OBC	1690	49	1738	50
Scheduled caste	647	19	690	20
Scheduled tribe	345	10	357	10
**Age at time of enrollment in years**				
18-24	2027	58	564	16
25-34	1391	40	2477	71
35+	66	2	443	13
**Education**				
Never been to school	347	10	100	3
Primary school or less	610	18	586	17
Middle school	1042	30	932	27
High school	1168	34	1322	38
Higher education	317	9	544	16
**MNO**				
Airtel	893	26	791	23
Idea	1572	45	967	28
Jio	229	7	1270	36
Tata	9	0	4	0
vodafone	781	22	427	12
BSNL			24	1
**Frequency of most recent top up**				
More than 3 months	299	9		
Within 1 month	1626	47		

Within 1 week	718	21		
Within 3 months	841	24		
**Who topped up credit**				
Husband	2784	80		
Other	357	10		
self	343	10		
**Who taught respondent how to use phone**				
Husband	794	23		
Other	178	5		
Self	2512	72		
**Permission for wife's phone use**				
Wife takes permission to make call	1133	33		
Wife takes permission before picking up call	1614	46		
Wife takes permission to recharge	838	24		
Women need oversight to use phone	2514	72		
**Type of phone**				
Brick phone	454	13	357	10
Feature phone	2206	63	1234	35
Smart phone	824	24	1838	53
**Use phone to call spouse**	2563	74	2926	84
**Use phone to call ASHAs**	293	8	2478	71
**Use phone for internet**	1	0	1417	41
**Use phone to listen radio**	1	0	1868	54
**Observe phone**				
Phone working	2820	81	3251	93
**Digital Tasks**				
Able to navigate IVR prompts	2995	86	3319	95
Give a missed call	2409	69	2890	83
Store contacts on phone	2845	82	2999	86
Open SMS	1654	47	2966	85
Read SMS	1102	32	2188	63
Overall digital literacy	937	27	1938	56
Open and read SMS	1102	32	2188	63
**Involvement in Decision making**				
About daily household expenditures	713	20	2065	59
About big expenditures	623	18	2243	64

About health during pregnancy	937	27	3081	88
**Employment status**	1398	40	3458	99
**Socio-economic status**				
Poorest	542	16	542	16
Poorer	646	19	646	19
Middle	710	20	710	20
Richer	760	22	760	22
Richest	826	24	826	24
**Phone in the household**				
1	759	22	759	22
2	1437	41	1437	41
>2	1288	37	1288	37
**Parity**				
No child	1406	40	1406	40
One child	1256	36	1256	36
Two and more	822	24	822	24
**Religion**				
Hindu	3297	95	3297	95
Muslim	183	5	183	5
Other	4	0	4	0
**Frequency of phone use in last 3 months**				
Every day	2700	77		
not every day	784	23		
**Age at marriage**				
0-15 years	416	12		
>15 years	3068	88		

Supplementary Table 2. Metrics used for cluster validation (Davies-Bouldin and Calinski-Harabatz criterions have been normalized to [0,1] ,1 indicating a good partition)

Number of clusters	**Within cluster sum of square**	**Silhouette index**	**Ray -Turi index**	**Calinski – Harabatz index**
**2**	64791,07	0,812424	0,873942	0,820123
**3**	62595,37	0,801119	1	0,9563
**4**	60983,52	0,509252	0,853942	0,360082
**5**	59662,45	0,466859	0,529231	0,243941
**6**	58571,27	0,454165	0,482203	0,161834
**7**	57686,73	0,420884	0,427094	0,096974
**8**	56943,46	0,402445	0,249373	0,044445
**9**	56322,05	0,386873	0,268434	0

Table 3a. Men's sample characteristics by cluster based on Men's survey data from four districts of Madhya Pradesh

	**Total** n=3,484		**Cluster 1** n=1,408		**Cluster 2** n=666		**Cluster 3** n=1,410	
	%	n	%	n	%	n	%	n
**Sociodemographic characteristics**								
**Caste**								
General	20	698	15	208	17	112	27	378
OBC	50	1 738	45	637	50	334	54	767
Scheduled tribe	10	357	15	213	11	73	5	71
Scheduled caste	20	690	25	350	22	146	14	194
**Education**								
Never been to school	3	100	7	92	1	6	-	2
Primary school or less	17	586	29	403	13	84	7	99
Middle school	27	932	32	446	28	189	21	297
High school	38	1 322	29	415	42	280	44	627
Higher education	16	544	4	52	16	107	27	385
**Number of phones in the household**								
0-1	22	759	34	476	24	157	9	126
2	41	1 437	45	629	43	284	37	524
3+	37	1 288	22	303	34	225	54	760

**Phone ownership and sharing**								
Own phone and do not share	17	578	16	221	8	50	22	307
Own phone and do share	78	2 730	73	1 031	91	607	77	1 092
Share only	3	93	5	73	1	9	1	11
**Phone type (observed)**								
Brick phone	10	357	22	304	3	17	3	36
Feature phone	35	1 234	68	953	23	151	9	130
Smart phone	53	1 838	7	96	75	498	88	1 244
**Men's phone use**								
Daily phone use (reported)	95	3 327	89	1 260	99	662	100	1 405
**Phone features used (reported)**								
Calls	98	3 422	96	1 350	100	666	100	1 406
SMS	46	1 615	19	263	55	369	70	983
WhatsApp	61	2 109	7	97	95	635	98	1 377
Watch video	80	2 784	52	726	99	659	99	1 399
Share video	58	2 008	6	87	89	591	94	1 330
Make video	35	1 209	9	121	47	316	55	772
Download Apps	47	1 640	2	29	70	468	81	1 143
Music	86	2 984	68	959	97	649	98	1 376
Radio	26	889	14	200	32	210	34	479
Search Google	55	1 925	9	128	82	548	89	1 249
Search YouTube	67	2 327	21	300	98	653	97	1 374
Camera	84	2 921	61	857	99	659	100	1 405
Share photo	59	2 039	7	93	90	602	95	1 344
Mobile money	16	560	0	3	15	103	32	454
Transfer mobile money	13	463	0	1	12	82	27	380
Transfer mobile credit	13	459	0	1	12	83	27	375
**Men's Digital skills (observed)**								
Able to navigate IVR prompts	95	3 319	91	1 280	98	656	98	1 383
Give a missed call	83	2 890	72	1 020	88	588	91	1 282
Store contacts on phone	86	2 999	73	1 031	94	623	95	1 345
Open SMS	85	2 966	71	994	94	624	96	1 348
Read SMS	63	2 188	38	530	73	483	83	1 175
Overall Basic Digital Skill Level	56	1 938	29	415	65	432	77	1 091
**WhatsApp skills (observed)**								
Open WhatsApp	58	2 017	6	91	91	605	94	1 321
Send WhatsApp text	49	1 718	3	44	75	498	83	1 176
Send WhatsApp voice note	49	1 719	3	42	73	488	84	1 189
**Watch video on phone (observed)**	74	2 568	43	603	94	624	95	1 341
**Men report getting images and videos from**								

Internet: YouTube	59	2 062	19	274	83	554	88	1 234
Internet: Google	45	1 569	9	130	64	429	72	1 010
Other relatives	36	1 249	4	63	54	360	59	826
Friends locally	55	1 916	11	153	83	550	86	1 213
Friends other states	25	885	1	21	36	238	44	626
**Computer/ tablet ownership and use**								
Own Computer/ tablet	6	220	1	13	4	28	13	179
Daily computer / tablet use	5	184	0	3	5	30	11	151
Ever use of the internet from any device/ location (reported)	66	2 305	32	447	87	580	91	1 278
Daily internet use in last 3 months (reported)	55	1 906	14	199	77	515	85	1 192
**Wife owns phone**	57	3 484	42	591	-	-	100	1 410
**Wife's phone type**								
Brick phone	10	363	10	134	0	1	16	228
Feature phone	29	1 016	27	375	-	-	45	641
Smart phone	19	647	8	106	-	-	38	541
**Wife shares phone with**								
Husband	44	1 543	33	461	-	-	77	1 082
Children (male or female)	5	180	4	52	-	-	9	128
Parents in law	9	329	6	83	-	-	17	246
Wife's parents	3	107	2	33	-	-	5	74
Other relatives	58	2 028	44	615	0	3	100	1 410
Friend/ neighbour	1	30	1	9	-	-	1	21
**Phone features wife uses (reported)**								
Calls: receive, dial, or speak	100	3 475	100	1 404	100	663	100	1 408
SMS	33	1 146	16	228	28	185	52	733
WhatsApp	35	1 225	11	155	38	255	58	815
Watch shows	54	1 871	26	368	68	450	75	1 053
Music or radio	100	3 484	100	1 408	100	666	100	1 410
Search internet	34	1 192	12	168	36	240	56	784
Camera	74	2 589	55	772	84	559	89	1 258
**Men's perceptions about restrictions (if any) which should be placed on phone use**								
**No restrictions should be placed on adult phone use**	86	2 992	85	1 192	86	571	87	1 229
**Oversight needed for**								
Men	47	1 647	54	767	46	307	41	573
Women	72	2 514	79	1 114	71	476	66	924
Male children	82	2 863	86	1 207	79	523	80	1 133
Female children	92	3 198	93	1 311	91	608	91	1 279
**Men report that their wife needs their permission to pick up**								

**calls from**								
Someone unknown	46	1 614	46	653	51	341	44	620
Family	13	461	17	237	18	122	7	102
Friends/ Neighbours	32	1 121	35	488	41	274	25	359
Health workers	22	757	25	356	29	195	15	206
Business associates	28	990	29	410	35	232	25	348
**Men report women need their permission to make a call to**								
Family	17	600	21	293	24	162	10	145
Friends/ Neighbours	21	735	25	345	28	187	14	203
Health workers	20	692	22	315	29	192	13	185
Business associates	14	484	17	236	16	109	10	139
Unknown to husband	17	608	20	286	20	134	13	188
**Men report women need their permission to send SMS or WhatsApp to**								
Family	2	72	1	12	4	28	2	32
Friends/ Neighbours	3	101	1	12	6	41	3	48
Health workers	2	77	1	9	5	30	3	38
Business associates	2	54	1	11	3	18	2	25
Unknown to husband	3	100	1	13	5	35	4	52
**Man has concerns about wife's phone ownership or use**	1	24	1	10	2	11	0	3
**Reasons for concern (multi-select):**								
Cost of phone	0	3	0	1	0	2	-	-
Cost of using phone	0	9	0	4	0	2	0	3
Reputational risk	0	13	0	5	1	8	-	-
Relationships with other men	0	3	0	2	0	1	-	-
Bad friendships with other women	0	3	0	1	0	2	-	-
Financially defrauded	0	1	-	-	0	1	-	-
**Men would like their wives to use the mobile phone to**								
Transfer money	41	1 439	30	423	42	281	52	735
Buy/ pay for things	37	1 304	26	368	38	256	48	680

Table 3b. Women's sample characteristics by cluster based on women's baseline survey data from four districts of Madhya Pradesh

	Total n=3,484		Cluster 1 n=1,408		Cluster 2 n=666		Cluster 3 n=1,410	
	%	n	%	n	%	n	%	n
**Sociodemographic characteristics**								
**Socioeconomic status**								
Poorest	16	542	26	369	13	88	6	85
Poorer	19	646	27	379	18	117	11	150
Middle	20	710	22	313	25	167	16	230
Richer	22	760	15	214	25	165	27	381
Richest	24	826	9	133	19	129	40	564
**District**								
Hoshangabad	10	345	11	151	11	76	8	118
Mandsaur	19	676	13	181	14	95	28	400
Rajgarh	23	791	21	302	29	191	21	298
Rewa	48	1 672	55	774	46	304	42	594
**Mean age (years)**	72	3 484	25	1 408	23	666	24	1 410
**Ethnicity/Caste**								
General	22	780	17	242	19	129	29	409
OBC	49	1 690	45	628	48	321	53	741
Scheduled caste	19	647	23	322	21	140	13	185
Scheduled tribe	10	345	14	203	11	72	5	70
**Education**								
Never been to school	10	347	16	229	8	50	5	68
Primary school or less	18	610	23	327	17	114	12	169
Middle school	30	1 042	32	451	35	236	25	355
High school	34	1 168	26	363	33	223	41	582
Higher education	9	317	3	38	6	43	17	236
**Phone ownership and sharing**								
Own phone and do not share	51	1 781	43	609	38	256	65	916
Own phone and share	22	772	23	318	22	145	22	309
Share only	26	923	34	475	40	264	13	184
**Phone type (observed)**								
Brick phone	7	248	8	113	8	50	6	85
Feature phone	63	2 206	74	1 040	54	359	57	807
Smart phone	24	824	11	158	28	188	34	478
No phone observed	6	206	7	97	10	69	3	40
**Women's phone characteristics**								
**Phone features (observed)**								
Call	79	2 765	76	1 072	71	470	87	1 223

Speaker	79	2 762	76	1 072	71	470	87	1 220
SMS	79	2 768	76	1 074	71	471	87	1 223
Contacts	79	2 766	76	1 072	71	471	87	1 223
Camera	66	2 302	63	889	60	398	72	1 015
Music/ audio content	69	2 419	66	923	63	419	76	1 077
Internet	49	1 712	42	596	47	312	57	804
Bluetooth	64	2 243	60	842	59	390	72	1 011
Radio/FM	69	2 416	64	907	62	415	78	1 094
**Applications installed on phone (observed)**								
Facebook	25	859	17	237	23	156	33	466
WhatsApp	17	603	8	113	18	117	26	373
Shareit	10	364	4	61	11	71	16	232
**Proportion of phones with zero balance at time of interview**	48	1 666	47	655	50	334	48	677
**Who topped up credit?**								
Husband	80	2 784	79	1 109	81	537	81	1 138
Self	10	357	11	157	12	79	9	121
Other	10	343	10	142	8	50	11	151
**Frequency of most recent top-up**								
Within 1 week	21	718	24	343	19	125	18	250
Within 1 month	47	1 626	46	645	46	309	48	672
Within 3 months	24	841	21	299	23	155	27	387
More than 3 months	9	299	9	121	12	77	7	101
**Total amount of last top up**								
>50	55	1 902	59	831	47	311	54	760
0-50	45	1 582	41	577	53	355	46	650
**Women's phone use**								
**Digital skill (observed)**								
Able to navigate IVR prompts	69	2 409	81	1 142	87	578	90	1 275
Give a missed call	82	2 845	64	895	60	401	79	1 113
Store contacts on phone	47	1 654	73	1 021	83	555	90	1 269
Open SMS	32	1 102	33	471	39	263	65	920
Read SMS	32	1 102	18	255	26	171	48	676
Overall Basic Digital Skill Level	27	937	15	213	21	139	41	585
**Communication**	74	2 563	65	917	68	455	84	1 191
Call with spouse	73	2 542	81	905	80	454	89	1 183
Call with friends, relatives	43	1 485	83	478	87	297	82	710
Call with health workers	32	1 132	99	317	99	196	97	619
SMS with husband	16	545	97	103	99	91	96	351

SMS with friends, relatives	9	330	98	45	100	49	100	236
SMS with health workers	6	213	100	27	100	24	99	162
Dialled a number and listened to pre-recorded message	77	2 700	72	1 010	73	489	85	1 201
**Who taught respondent how to use phone?**								
Spouse	5	178	5	72	5	35	5	71
Self	72	2 512	70	986	71	472	75	1 054
Other	23	794	25	350	24	159	20	285

Supplementary Table 4. Strong signals (variable used for the spide charts are highlighted)

	Cluster 1 (n=1408)	Cluster 2 (n=666)	Cluster 3 (n=1410)
**Men paid for wife's balance**	37	0	90
**Men can perform basic internet search**	7	66	77
**Men report that their wife uses prepaid pack**	42	0	100
**Men report that women need their permission to add credit**	18	0	42
**Men report ever use of internet**	31	87	91
**Observe men watching Video**	42	93	95
**Men can send WhatsApp text**	3	77	85
**Men report use of WhatsApp**	7	91	95
**Men report that their wife's use the phone to**			
Search internet	12	36	55
Watch show	26	66	75
WhatsApp	11	37	57
Men report that they can send photo on WhatsApp	4	88	93
Men report that they can send a WhatsApp voice message	3	73	84
**Men report getting images and videos from**			
Internet: YouTube	19	84	88
Internet: Google	9	64	71
Other relatives	4	55	59
Friends locally	11	83	87
Friends other states	2	36	44
**Men report not using the internet frequently**	86	23	15
**Men have smart phone**	6	75	88
**Men report using the internet frequently**	14	77	85
**Men have feature phone**	68	23	9
**Number of phones in the household**			
3+	19	32	61
0-1	43	39	2
**Men report that their wife own's a phone**	42	0	100
**Men report that their wife does not own a phone**	58	100	0
**Men report their wife shares phone she owns with husband**	32	0	77
**Men observed to open WhatsApp**	6	91	94
**Men's observed digital literacy**	29	64	77
**Men observed to read SMS**	37	72	82
**Features men report using on their phone**			
Share photo	7	90	96
Search YouTube	21	98	98
Search Google	9	82	88
Download Apps	2	70	82
Make video	8	48	55
Share video	6	88	94
Watch video	51	99	99
WhatsApp	7	95	98
SMS	18	55	69
**Observe TikTok App on men's phone**	1	36	48
**Men have internet in their household**	25	54	69
**Men report women having a phone other than Samsung or Jio**	24	0	53

**Men report that women have a feature phone**	26	0	46

Supplementary Figure 1. PCA with 95% of cumulative explained variance on couples' data.

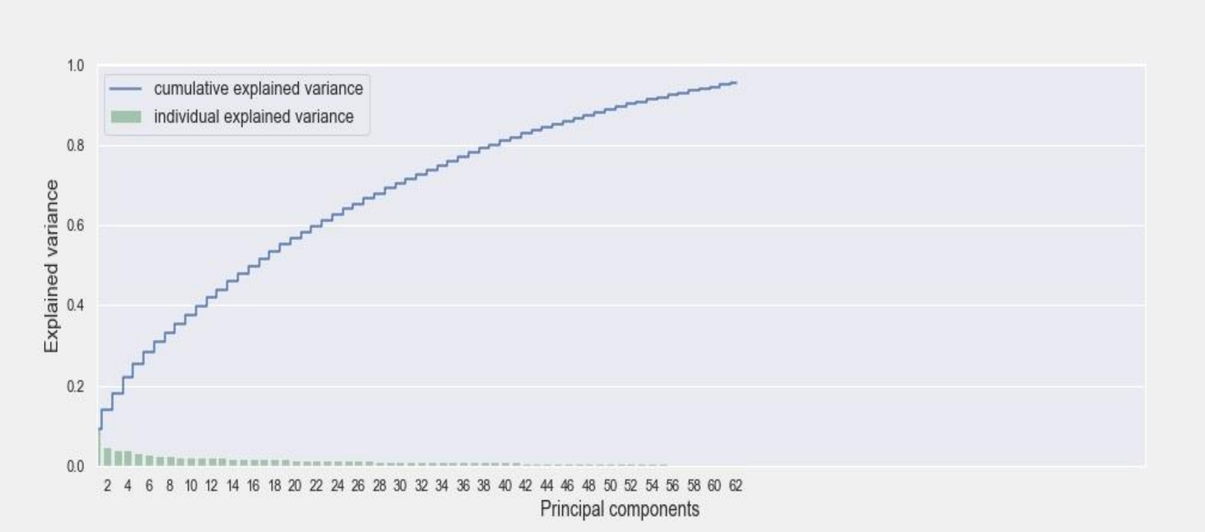

Supplement: Supplementary data [file bmjopen-2022-063354supp001.pdf]
