# Supplementary material for: Can we design the next generation of digital health communication programs by leveraging the power of artificial intelligence to segment target audiences, bolster impact and deliver differentiated services? A machine learning analysis of survey data from rural India
Source: BMJ Open. 2023 Mar 17;13(3):e063354. doi: 10.1136/bmjopen-2022-063354 (PMC10030469; doi:10.1136/bmjopen-2022-063354)
Supplement: Author's manuscript [file bmjopen-2022-063354.draft_revisions.pdf]

BMJ Open is committed to open peer review. As part of this commitment we make the peer review history of every article we publish publicly available.

When an article is published we post the peer reviewers' comments and the authors' responses online. We also post the versions of the paper that were used during peer review. These are the versions that the peer review comments apply to.

The versions of the paper that follow are the versions that were submitted during the peer review process. They are not the versions of record or the final published versions. They should not be cited or distributed as the published version of this manuscript.

BMJ Open is an open access journal and the full, final, typeset and author-corrected version of record of the manuscript is available on our site with no access controls, subscription charges or pay-per-view fees (http://bmjopen.bmj.com).

If you have any questions on BMJ Open's open peer review process please email info.bmjopen@bmj.com

BMJ Open

Can we design the next generation of digital health communication programs by leveraging the power of artificial intelligence to segment target audiences, bolster impact, and deliver differentiated services? A machine learning analysis of survey data from rural India

Journal:	*BMJ Open*
Manuscript ID	bmjopen-2022-063354
Article Type:	Original research
Date Submitted by the Author:	06-Apr-2022

Complete List of Authors:	Bashingwa, Jean ; University of Cape Town Faculty of Health Sciences,<br>Mohan, Diwakar; Johns Hopkins University Bloomberg School of Public Health<br>Chamberlain, Sara; BBC Media Action, BBC Media Action, India; BBC Media Action, Asia<br>Scott, Kerry; Johns Hopkins University Bloomberg School of Public Health;<br>Ummer, Osama; Oxford Policy Management, ; BBC Media Action,<br>Godfrey, Anna; BBC Media Action,<br>Mulder, Nicola; University of Cape Town<br>Moodley, Deshen; University of Cape Town, Department of Computer Science<br>LeFevre, Amnesty; Johns Hopkins University, International Health

Keywords:	Public health < INFECTIOUS DISEASES, HEALTH ECONOMICS, Community child health < PAEDIATRICS

SCHOLARONE™
Manuscripts

I, the Submitting Author has the right to grant and does grant on behalf of all authors of the Work (as defined in the below author licence), an exclusive licence and/or a non-exclusive licence for contributions from authors who are: i) UK Crown employees; ii) where BMJ has agreed a CC-BY licence shall apply, and/or iii) in accordance with the terms applicable for US Federal Government officers or employees acting as part of their official duties; on a worldwide, perpetual, irrevocable, royalty-free basis to BMJ Publishing Group Ltd ("BMJ") its licensees and where the relevant Journal is co-owned by BMJ to the co-owners of the Journal, to publish the Work in this journal and any other BMJ products and to exploit all rights, as set out in our licence.

The Submitting Author accepts and understands that any supply made under these terms is made by BMJ to the Submitting Author unless you are acting as an employee on behalf of your employer or a postgraduate student of an affiliated institution which is paying any applicable article publishing charge ("APC") for Open Access articles. Where the Submitting Author wishes to make the Work available on an Open Access basis (and intends to pay the relevant APC), the terms of reuse of such Open Access shall be governed by a Creative Commons licence – details of these licences and which Creative Commons licence will apply to this Work are set out in our licence referred to above.

Other than as permitted in any relevant BMJ Author's Self Archiving Policies, I confirm this Work has not been accepted for publication elsewhere, is not being considered for publication elsewhere and does not duplicate material already published. I confirm all authors consent to publication of this Work and authorise the granting of this licence.

For peer review only

Can we design the next generation of digital health communication programs by leveraging the power of artificial intelligence to segment target audiences, bolster impact, and deliver differentiated services? A machine learning analysis of survey data from rural India

Jean Juste Harrisson Bashingwa, PhD (corresponding author)
MRC/Wits-Agincourt Unit, School of Public Health, University of the Witwatersrand, 27 St. Andrews Road, Parktown, 2193, South Africa
Email: jeanjuste@aims.ac.za

Diwakar Mohan, DrPH
Department of International Health, Johns Hopkins Bloomberg School of Public Health, 615 N. Wolfe St, Baltimore, Maryland, USA
Email: dmohan3@jhu.edu

Sara Chamberlain, MA
Innov8 Old Fort Saket District Mall, Saket District Centre, Sector 6, Pushp Vihar, New Delhi, Delhi 110017, India
Email: sara.chamberlain@in.bbcmediaaction.org

Kerry Scott, PhD
Department of International Health, Johns Hopkins Bloomberg School of Public Health, 615 N. Wolfe St, Baltimore, Maryland, USA
Email: kscott26@jhu.edu

Osama Ummer, MHA
(1) BBC Media Action-India, Innov8 Old Fort Saket District Mall, Saket District Centre, Sector 6, Pushp Vihar, New Delhi, Delhi 110017, India
(2) Oxford Policy Management-Delhi, 4/6 First Floor, Siri Fort Institutional Area, New Delhi, Delhi 110049, India
Email: kposamaummer@gmail.com

Anna Godfrey, PhD
BBC Media Action, Ibex House, 42-47 Minories, London, EC3N 1DY, England
Email: anna.godfrey@bbc.co.uk

Nicola Mulder, PhD
Computational Biology Division, Department of Integrative Biomedical Sciences, Institute of Infectious Disease and Molecular Medicine, Faculty of Health Sciences, University of Cape Town
Anzio Road, Observatory, 7925, Cape Town, South Africa
Email: nicola.mulder@uct.ac.za

Deshen Moodley, PhD
Department of Computer Science,
18 University Avenue, University of Cape Town
Rondebosch, Cape Town, South Africa
Email: deshen@cs.uct.ac.za

For peer review only

Amnesty E. LeFevre PhD
1. School of Public Health and Family Medicine, University of Cape Town, Falmouth Rd, Observatory, Cape Town, 7925, South Africa
2. Department of International Health, Johns Hopkins Bloomberg School of Public Health, 615 N. Wolfe St, Baltimore, Maryland, USA
Email: aelefevre@gmail.com

Abstract (268 of 300 words)
Objectives
Direct to beneficiary (D2B) mobile health communication programs have been used to provide reproductive, maternal, neonatal and child health (RMNC) information to women and their families in a number of countries globally. Programs to date have provided the same content, at the same frequency, using the same channel to large beneficiary populations. This manuscript presents a proof of concept approach that uses machine learning to segment populations of women with access to phones and their husbands into distinct clusters to support differential digital program design and delivery.
Setting
Data used in this study were drawn from cross-sectional survey conducted in four districts of Madhya Pradesh, India.
Participants
Study participant included pregnant women with access to a phone (n=5,095) and their husbands (n=3,842)
Results
We used an iterative process involving K-means clustering and Ridge regression to segment couples into three distinct clusters. Cluster 1 (n=1,408) tended to be poorer, lessor educated men and women, with low levels of digital access and skills. Cluster 2 (n=666) had a mid-level of digital access and skills among men but not women. Cluster 3 (n=1,410) had high digital access and skill among men and moderate access and skills among women. Exposure to the D2B program 'Kilkari' showed the greatest difference in Cluster 2, including an 8% difference in use of reversible modern contraceptives, 7% in child immunisation at 10 weeks, 3% in child immunisation at 9 months, and 4% in the timeliness of immunisation at 10 weeks and 9 months.
Conclusions
Findings suggest that segmenting populations into distinct clusters for differentiated program design and delivery may serve to improve reach and impact.

For peer review only

Summary Box:
What is already known?
- Direct to beneficiary mobile health communication programs have a significant impact on some health behaviours but not all.
- The magnitude of impact has additionally been observed to vary based on beneficiary characteristics, including sociodemographic characteristics and digital access and use.

What are the new findings?
- Machine learning can be used to segment populations of women with access to phones and their husbands into distinct clusters for differential program design and delivery.
- Data on observed and reported mobile phone characteristics, access and use were integral to developing distinct clusters.

What do the new findings imply?

- Segmenting populations into distinct clusters for differentiated program design and delivery may serve to increase the reach and deepen the impact of mobile health communication programs.

Introduction

Digital health solutions have the potential to address critical gaps in information access and service delivery, which underpin high mortality [1-4]. Mobile health communication programs, which provide information directly to beneficiaries, are among the few examples of digital health solutions to have scaled widely in a range of settings [5, 6]. Historically, these solutions have been designed as 'blunt instruments' – providing the same content, with the same frequency, using the same digital channel to large target populations. While this approach has enabled solutions to scale, it has contributed to variability in their reach and impact, due in part to differences in women's access to and use of mobile phones, particularly in low- and middle-income countries [7, 8].

Despite near ubiquitous ownership of mobile phones at a household level, a growing body of evidence suggests that there is a substantial gap between men and women's ownership, access to and use of mobile phones [9-11]. In India, there is a 45% gap between women's reported access to a phone and ownership at a household level [11]. Variations in the size of the gap have been observed across states and urban/rural areas, and by sociodemographic characteristics, including education, caste, and socioeconomic status [11]. Amongst women with reported access to a mobile phone, the gender gap further persists in the use of mobiles, in part because of patriarchal gender norms and limited digital skills [12]. Collectively, these gender gaps underscore the need to consider inequities in phone access and use patterns when designing and implementing D2B mobile health communication programs.

Kilkari, designed and scaled by BBC Media Action in collaboration with the Ministry of Health and Family Welfare, is India's largest direct to beneficiary mobile health information program. When BBC Media Action transitioned Kilkari to the national government in April 2019, it had been implemented in 13 states and reached over 10 million women and their families [13, 14]. Evidence on the program's impact from a randomized control trial conducted in Madhya Pradesh, India, between 2018 and 2021, suggests that across study arms, Kilkari was associated with a 3.7% increase in modern reversible contraceptive use (RR: 1.12, 95% CI: 1.03 to 1.21, p=0.007), and a 2.0% decrease in the proportion of male or females sterilized since the birth of the child (RR: 0.85, 95% CI: 0.74 to 0.97, p=0.016) [14]. The program's impact on contraceptive use, however, varied across key population sub-groups. Among women exposed to 50% or more of the Kilkari content as compared to those not exposed, differences in reversible method use were greatest for those in the poorest socioeconomic strata (15.8% higher), for those in disadvantaged castes (12.0% higher), and for those with any male child (9.9% higher) [14]. Kilkari's overall and varied impact across beneficiary groups raises important questions about whether the differential targeting of women and their families might lead to efficiency gains and deepen impact.

In this manuscript, we argue that to maximize reach, exposure, and deepen impact, the future design of mobile health communication solutions will need to consider the heterogeneity of beneficiaries, including

For peer review only

within husband-wife couples, and move away from a one-size-fits all model towards differentiated program design and delivery. Drawing from husbands' and wives' survey data captured as part of a randomised controlled trial of Kilkari in Madhya Pradesh India, we used a three-step process involving K-means clustering and Ridge regression to segment couples into distinct clusters. We then assess differences in health behaviours across respondents in both study arms of the RCT. Findings are anticipated to inform future efforts to capture data and refine methods for segmenting beneficiary populations and in turn optimizing the design and delivery of mobile health communication programs in India and elsewhere globally.

Methods

Kilkari program overview

Kilkari is an outbound service that makes weekly, stage-based, pre-recorded calls about reproductive, maternal, neonatal and child health (RMNCH) directly to families' mobile phones, starting from the second trimester of pregnancy until the child is one year old. Kilkari is comprised of 90 minutes of reproductive, maternal, newborn and child health content sent via 72 once weekly voice calls (average call duration: 1 minute, 15 seconds). Approximately 18% of cumulative call content is on family planning; 13% on child immunisation; 13% on nutrition; 12% on infant feeding; 10% on pregnancy care; 7% on entitlements; 7% on diarrhoea; 7% on postnatal care; and the remainder on a range of topics including intrapartum care, water and sanitation (WASH), and early childhood development. BBC Media Action designed and piloted Kilkari in the Indian state of Bihar in 2012-2013, and then redesigned and scaled it in collaboration with the Ministry of Health and Family Welfare between 2015 and 2019. Evidence on the evaluation design and program impact are reported elsewhere [15].

Setting

Data used in this analysis were collected from four districts of the central Indian state of Madhya Pradesh as part of the impact evaluation of Kilkari described elsewhere [14]. Madhya Pradesh (population 75 million) is home to an estimated 20% of India's population and falls below national averages for most sociodemographic and health indicators [16]. Wide differences by gender and between urban and rural areas persist for wide range of indicators including literacy, phone access and health seeking behaviours. Among men and women 15-49 years of age, 59% of women (78% urban and 51% rural) were literate as compared to 82% of men in 2015-2016 [16]. Amongst literate women, 23% had 10 or more years of schooling (44% urban and 14% rural) [16]. Despite near universal access to phones at a household level, only 19% of women in rural areas and 50% in urban had access to a phone that they themselves could use in 2015 [16]. Among pregnant women, over half (52%) of pregnant women received the recommended four ANC visits in urban areas as compared to only 30% in rural areas [16]. Despite high rates of institutional delivery (94%) in urban areas, only 76% of women in rural areas reported delivering in a health facility in 2015 [16]. These disparities underscore the population heterogeneity within and across Madhya Pradesh.

Sample population

The sample for this study were obtained through cross-sectional surveys administered between 2018 and 2020 to women (n=5,095) with access to a mobile phone and their husbands (n=3,842) in four districts of Madhya Pradesh [15]. At the time of the first survey (2018-2019), the women were 4-7 months pregnant; the latter survey (2019-2020) re-interviewed the same women at 12 months postpartum. Their husbands were only interviewed once, during the latter survey round. The surveys spanned 1.5 hours in length. In this analysis, modules on household assets and member characteristics; phone access and use, including observed digital skills (navigate IVR prompts, give a missed call, store contacts on a phone, open SMS, read SMS) were used to develop models. Data on practice for maternal and child health behaviours, including infant and young child feeding, family planning, pregnancy and postpartum care were used to explore the differential impact of Kilkari across clusters but not used in the development of clusters [15].

Approach to segmentation

For peer review only - http://bmjopen.bmj.com/site/about/guidelines.xhtml

Figure 1 presents a framework used for developing homogenous clusters of men and women in four districts of rural Madhya Pradesh India. Box 1 describes the steps undertaken at each point in the framework in detail. We started with data elements collected on phone access and use as well as population sociodemographic characteristics collected as part of a cross-sectional survey described elsewhere [17]. Unsupervised learning was undertaken using K-Means cluster and strong signals were identified. Strong signals were defined as variables that had at least a prevalence of 70% in one or more clusters and differed from another cluster by 50% or more. For example, 6% of men own a smart phone in cluster 1, 88% in cluster 2 and 75% in cluster 3. Therefore, having a smart phone can be considered as a strong signal. Additional details are summarised in Box 1. Once defined, we then explored differences in health care practices across study clusters among those exposed and not exposed to Kilkari within each cluster.

Patient and public involvement

Patients were first engaged upon identification in their households as part of a household listing carried out in mid/ late 2018. Those meeting eligibility criteria were interviewed as part of the baseline survey, and ultimately randomized to the intervention and control arms. Prior to the administration of the baseline, a small number of patients were involved in the refinement of survey tools through qualitative interviews, including cognitive interviews, which were carried out to optimise survey questions, including the language and translation used. Finalised tools were administered to patients at baseline and endline, and for a sub-sample of the study population, additional interviews carried out over the phone and via qualitative interviews between the baseline and endline surveys. Unfortunately because of COVID-19 patients and associated travel restrictions could not be involved in the dissemination of study findings.

For peer review only

Box 1. Step-wise process for developing and refining a machine learning approach for population segmentation

Data collected from special surveys like the couple's data set used here are relatively smaller in terms of sample size but large with regard to the number of data elements available. In such high dimensional data, there are many irrelevant dimensions which can mask existing clusters in noisy data, making more difficult the development of effective clustering methods [18]. Several approaches have been proposed to address this problem. They can be grouped into two categories: static or adaptive *dimensionality reduction*, including principal components analysis (PCA) [19, 20] and *subspace clustering* consisting on selecting a small number of original dimensions (features) in some unsupervised way or using expert knowledge so that clusters become more obvious in the subspace [21, 22] . In this study we combined subspace clustering using expert knowledge and adaptive dimensionality reduction (Supplementary Figure 1) to find subspace where clusters are most well separated and well defined. Therefore, as part of subspace clustering, we chose to start with couples' survey data, including variables related to socio demographic characteristic, phone ownership, use and literacy (Supplementary Table 1). Emergent clusters were overlapping. We decided to use men's survey data on phone access and use as a starting point.

Step 1. Defining variables which characterise homogenous groups

Analyses started with a predefined set of data elements captured as part of a men's cross-sectional survey including sociodemographic characteristics and phone access and use. K-Means clustering was used to identify clusters and the elbow method was used to define the optimal number of clusters. Strong signals were then identified. Variables which had at least a prevalence of 70% in one or more clusters and differed from another cluster by 50% or more were considered to have a strong signal.

Step 2. Model strengthen through the identification and addition of new variables

Once an initial model was developed drawing from the predefined set of data from the men's survey and strong signals were identified, we reviewed available data from the combined dataset (data from the men's survey and women's survey). Signal strength was used as an outcome variable or target in a linear regression with L1 regularization or Lasso regression (Least Absolute Shrinkage and Selection Operator). Regularization is a technique used in supervised learning to avoid overfitting. Lasso Regression adds absolute value of magnitude of coefficient as penalty term to the loss function. The loss function becomes:

$$Loss = Error(y,y) + \alpha \sum_{i=1}^{N} |\omega_i|$$

where ω_i are coefficients of linear regression $y = \omega_1 x_1 + \omega_2 x_2 + ... + \omega_N x_N + b$

Lasso Regression works well for selecting features in very large datasets as it shrinks the less important features of coefficients to zero [23, 24]. Merged women's survey and men's survey data were used as predictors for the regression, excluding variables related to heath knowledge and practices. We ended up with a sample of 3,484 rows and 1,725 variables after data pre-processing.

Step 3. Refining clusters using supervised learning

We then re-ran K-Means clustering with three clusters (K=3) using important features selected by lasso regression. This methodology was used to refine the clusters and subsequently identify new strong signals. After step 3 was conducted, we repeated step 2, and kept on iteratively repeating step 2 and 3 until there was no gain in strong signals.

Figure 1. Framework for segmentation analysis

For peer review only

K-Means algorithm

As part of Steps 1 and 3, K-means algorithms were used (Box 1). A K-Means algorithm is one method of cluster analysis designed to uncover natural groupings within a heterogeneous population by minimizing Euclidean distance between them [25]. When using a K-Means algorithm, the first step is to choose the number of clusters K that will be generated. The algorithm starts by selecting K points randomly as the initial centres (also known as cluster means or centroids) and then iteratively assigns each observation to the nearest centre. Next, the algorithm computes the new mean value (centroid) of each cluster's new set of observation. K-Means re-iterates this process, assigning observations to the nearest centre. This process repeats until a new iteration no longer reassigns any observations to a new cluster (convergence). Four metrics have been used for the validation of clustering: within cluster sum of squares, silhouette index, Ray-Turi criterion and Calinski-Harabatz criterion. Elbow method was used to find the right K (number of clusters) [26]. Figure 2 is a chart showing the within cluster sum of squares (or inertia) by the number of groups (k value) chosen for several executions of the algorithm.

Figure 2. Elbow method used to help decide ultimate number of clusters appropriate for the data.

Inertia is a metric that shows how dissimilar the members of a group are. The less inertia there is, the more similarity there is within a cluster (compactness). The main purpose of clustering is not to find 100% compactness, it is rather to find a fair number of groups that could explain with satisfaction a considerable part of the data (k=3 in this case). Silhouette analysis helped to evaluate the goodness of clustering or clustering validation (*Figure 3)*. It can be used to study the separation distance between the resulting clusters. The silhouette plot displays a measure of how close each point in one cluster is to points in the neighbouring clusters. This measure has a range of [-1, 1]. Silhouette coefficients near +1 indicate that the sample is far from the neighbouring clusters. A value of 0 indicates that the sample is very close to the decision boundary between two neighbouring clusters and negative values indicate that those samples might have been assigned to the wrong cluster. Figure 3 shows that choosing three clusters was more efficient than four for the data from the available surveys for two reasons: 1) there were less points with negative silhouettes, 2) the cluster size (thickness) was more uniform for three groupings. Other criterions used to evaluate quality of clustering are obtained by combining the 'within cluster compactness index' and 'between-cluster spacing index' [27]. Calinski-Harabatz criterion is given by: $C(k) = \frac{Trace(B)\ (n-k)}{Trace(W)\ (k-1)}$ and Ray-Turi criterion is given by $r(k) = \frac{distance\ (W)}{distance\ (B)}$ where B is the between-cluster covariance matrix (so high values of B denote well-separated clusters) and W is the within-cluster covariance matrix (so low values of W correspond to compact clusters) . They both ended up with same conclusions that 3 clusters were the best choice for the data we had. Supplementary Table 2 gives different metrics used and values obtained for various clusters.

Figure 3. Silhouette analysis for three and four clusters

Results

Sample characteristics

Supplementary Tables 3a and 3b summarise the sample characteristics by cluster for men and women interviewed. Figure 4 and Supplementary Table 4 presents select characteristics with 'strong signals' for each cluster.

Cluster 1 (n=1,408) constitutes 40% of the sample population and was comprised of men and women with low levels of digital access and skills (Figure 4). This cluster included the poorest segment of the sample population: 36% had a primary school or lower education and 40% were from a scheduled tribe/caste. Most

For peer review only - http://bmjopen.bmj.com/site/about/guidelines.xhtml

men owned a feature (68%) or brick phone (22%); used the phone daily (89%); and while able to navigate IVR prompts (91%), only 29% were able to perform all of the five basic digital skills assessed. Women in this cluster similarly had lower levels of education as compared to other clusters (39% have primary school or less education); used feature (74%) or brick phones (8%); and had low digital skills (15% were able to perform the five basic digital skills assessed).

Cluster 2 (n=666; 19% of sample population), is comprised of men with mid-level and women with low digital access and skills. In this cluster, 75% of men owned smartphones, 65% were observed to successfully perform the five basic digital skills assessed, and 36% could perform a basic internet search. Men in Cluster 2 also self-reported accessing videos from YouTube (84%) and using WhatsApp (95%). Women in Cluster 2 had low phone ownership; nearly half of women reported owning a phone (38% owned a phone and did not share it, 22% owned and shared a phone) — findings which contradict their husbands' reports of 0% women's phone ownership. Only 21% of women in this cluster were observed to be able to successfully perform the five basic digital skills assessed. However, based on husband's reporting of their wives' digital skills, 36% of women could search the internet, 37% used WhatsApp, and 66% watched shows on someone else's phone.

Cluster 3 (n=1,410; 40% of sample population) is comprised of couples with high level digital access among both husbands and wives, and lower-level digital skill among wives (Figure 4). An estimated 67% of couples in this cluster were in the richer or richest socioeconomic strata, while 71% of men and 58% of women had high school or higher levels of education. Men in this cluster reported using the internet frequently (85%), were observed to own smart phones (88%), and had high levels of digital skills: 77% could perform the five basic digital skills assessed, 77% could perform a basic internet search, and 85% could send a WhatsApp message When reporting on their wife's digital access and skills, all men in this cluster reported that their wives' owned phones (100%), but often shared these phones with their husbands (77%), using them to watch shows (75%), search the internet (55%), or use WhatsApp (57%). However, a much lower level of women interviewed in this cluster were observed to own Feature (57%) or Smart phones (34%) and had moderate digital skills with 41% being able to successfully perform the five basic digital skills assessed.

Figure 4. Distribution of select characteristics with strong signals by Cluster

Differences in health outcomes by Cluster

Table 1 presents differences in health outcomes by Cluster among those exposed and not exposed to Kilkari as part of the randomised controlled trial in Madhya Pradesh. Findings suggest that the greatest impact was observed among those exposed to Kilkari in Cluster 2, which is the smallest cluster identified (19% of the sample population). Amongst this population, differences between exposed and not exposed were 8% for reversible modern contraceptive methods, 7% for immunisation at 10 weeks, 3% for immunisation at 9 months, and 4% for timely immunisation at 10 weeks and 9 months. Additionally, an 8% difference between exposed and not exposed was observed for the proportion of women who report being involved in the decision about what complementary foods to give child.

Among Clusters 1 and 3, improvements were observed among those exposed to Kilkari for a small number of outcomes. In Cluster 1, those exposed to Kilkari had a 3-4% higher rate of immunisation at 6, 10, 14 weeks than those not exposed. In both Clusters 1 and 3 the timeliness of immunisation improved at 10 weeks amongst those exposed. No improvements were observed for use of modern reversible contraception in either cluster.

For peer review only

Table 1. Differential impact of Kilkari exposure on family planning, infant feeding and immunizations per cluster

	Cluster 1					Cluster 2					Cluster 3				
	Not exposed		Exposed		% difference	Not exposed		Exposed		% difference	Not exposed		Exposed		% difference
	%	n	%	n		%	n	%	n		%	n	%	n	
**Family planning**															
Current modern family planning use	42	269	41	316	-1	42	130	44	157	2	50	340	51	368	1
Reversible methods	29	183	30	232	1	30	94	38	133	8	41	280	44	319	3
Sterilized	12	77	10	80	-2	11	33	8	30	-3	10	66	7	54	-3
Sterilized	18	114	16	121	-2	15	47	12	44	-3	14	99	12	84	-2
**Infant and young child feeding**															
Immediate breastfeeding	96	610	95	736	-1	93	291	95	336	2	94	645	93	675	-1
Gave child semi solid food yesterday	98	624	99	762	1	99	309	99	350	0	99	676	98	715	-1
Exclusive breastfeeding	6	39	6	48	0	7	21	8	28	1	6	43	7	51	1
Fed child solid, semi-solid or soft foods the minimum number of times during the previous day	54	344	55	423	1	62	193	64	228	2	66	450	65	469	-1
Minimum acceptable diet	27	171	28	219	1	29	91	26	92	-3	25	170	27	198	2
Women involved in the decision about what complementary foods to give child	89	569	92	708	3	82	256	90	319	8	88	604	87	634	-1
**Immunization**															
Fully immunized	44	280	44	340	0	45	139	49	173	4	51	350	48	352	-3
Birth	70	444	70	542	0	71	223	73	259	2	72	493	74	534	2
6 weeks	75	475	78	600	3	78	242	79	280	1	77	528	78	568	1
10 weeks	72	460	76	584	4	72	225	79	279	7	75	514	76	554	1
14 weeks	68	432	71	550	3	74	230	74	263	0	75	511	75	541	0
9 months	68	433	68	522	0	69	214	72	255	3	75	510	74	538	-1
Timeliness: birth	69	438	67	515	-2	68	213	69	246	1	70	477	72	525	2
Timeliness: 6 weeks	45	287	46	353	1	45	139	44	155	-1	51	349	51	371	0
Timeliness: 10 weeks	25	162	28	217	3	23	71	27	94	4	31	213	34	248	3
Timeliness: 14 weeks	13	85	13	102	0	14	43	14	51	0	19	131	22	162	3
Timeliness: 9 months	14	89	13	99	-1	12	37	16	55	4	18	126	17	126	-1

Discussion

Evidence on the impact of direct to beneficiary mobile health communication programs is limited but broadly suggests that they can cost-effectively improve some reproductive, maternal and child health practices. This analysis aims to serve as a proof of concept for segmenting beneficiary populations to support the design of more targeted mobile health communication programs. We used a three-step iterative process involving a combination of supervised and unsupervised learning (K-means clustering and Lasso regression) to segment couples into distinct clusters. Three identifiable groups emerge each with differing health behaviours. Findings suggest that exposure the D2B program Kilkari may have a differential impact among the clusters.

Implications for designing future digital solutions

Findings demonstrate that the impact of the D2B solution Kilkari varied across homogenous clusters of women with access to mobile phones and their husbands in Madhya Pradesh. Across delivery channels, our analysis indicates that mobile health communication could not be effectively delivered to husbands and wives in Cluster 1 using WhatsApp, because smartphone ownership and WhatsApp use in this cluster are negligible. IVR, on the other hand, could be used to reach couples in Cluster 1, but reach is likely to be sporadic because of high levels of phone sharing with others (78% among men and 57% among women). On the other hand, WhatsApp and YouTube are likely to be effective digital channels for communicating with both husbands and wives in Cluster 3, where most men and women own or use smartphones and WhatsApp.

Beyond delivery channels, study findings raise a number of important learnings for content development as well as optimising beneficiary reach and exposure. The creative approach to content created for Cluster 3, where 40% of women are from the richest socio-economic status and only 17% have never been to school or have a Primary School education or less, would need to be very different from the creative approach to content created for Cluster 1, where 53% have a poorest or poorer socio-economic status, and 39% have never been to school or have a Primary School education or less. Similarly, this analysis adds to qualitative findings [12] and provides important insights into how gender norms related to women's use of mobile phones may effect reach and impact. While few (13-15%) husbands indicated that 'adults' need oversight to use mobile phones, men's perceptions varied when asked about specific use cases. Across all Clusters, nearly half of husbands indicated that their wives needed permission to pick up phone calls from unknown numbers – an important insight for IVR programs which may make outbound calls without pre-warning to beneficiaries. In Clusters 1 and 2, 25% and 29% of husband's, respectively, report that their wives need permission to answer calls from health workers – as compared to 15% in Cluster 3. While restrictions on SMS and WhatsApp were lower than making or receiving calls, these channels are less viable given women's limited access to smartphones, low literacy and digital skills. Overall, men's perceptions on the restrictions needed on the receipt and placement of calls by women was lower for Cluster 3. However, despite the relative wealth of beneficiaries in Cluster 3 (67% were in the richer or richest socioeconomic strata), 48% of women had zero balance on their mobile phones at the time of interview. Collectively, these findings highlight the immense challenges which underpin efforts to facilitate women's phone access and use. They too underline the criticality of designing mobile health communication content for couples, rather than just wives to ensure the buy-in of male gatekeepers, and for continuing to prioritize face to face communication with women on critical health issues.

Approach to segmentation

Data in our sample were captured as part of special surveys carried out through the impact evaluation of Kilkari. Future programs may be tempted to apply the approach undertaken here to existing datasets, including routine health information systems or other forms of government tracking data. In the India context, while these data are likely to be less costly than special surveys, they are comparatively limited in terms of data elements captured – particularly in terms of data ownership of different types of mobile devices, digital skill levels and usage of specific applications or social media platforms. Data quality may

For peer review only

also be a significant issue in existing datasets (ref). For example, we estimate that SIM change in our study population was 44% over a 12-month period – a factor which when coupled with the absence of systems to update government tracking registries raises important questions about who is retained in these databases, and therefore able to receive mobile health communications—and who is missing. Amongst the variables used, men's phone access and use were most integral to developing distinct clusters. We recommend that future surveys seeking to generate data for designing digital services for women ensure that data elements are captured on men's phone access and use practices as well as their perception of their wife's phone access and use.

In addition to underlying data, our analytic approach differed from other segmentation analyses which consist exclusively of unsupervised learning [28, 29] or supervised learning [30, 31]. Data collected from special surveys like the couple's data set used here are comparatively smaller in terms of sample size but large with regard to the number of data elements available. An alternative approach to that described in this manuscript might be to develop strata based on population characteristics. Indeed, findings from the impact evaluation published elsewhere suggest that women with access to phones in the most disadvantaged sociodemographic strata (poorest (15.8% higher) and disadvantaged castes (12% higher)) had greater impact when exposed to 50% or more of the Kilkari content as compared to those not exposed. With an approach to segmentation based on these strata of highest impact, we know and understand what divides or groups respondents (e.g. socioeconomic status, education) but this may not be enough when they do not explain the underlying reasons for change. In the approach used here, the study population is segmented using multiple characteristics (sociodemographic, digital access and use) simultaneously. The results are clusters comprised of individuals with mixed sociodemographic characteristics which may help to explain the reduced impact observed on health outcomes. Designing a strategy based on previously known / identifiable strata alone has been the basis of targeting in public health but has not maximized reach, exposure and effect to its fullest potential. The approach used here may better group beneficiaries based on their digital access and use characteristics which may serve to increase reach and exposure. However, further research is needed to determine how to deepen impact within these digital clusters.

Limitations

There are several limitations while interpreting our findings. First, data were drawn from surveys conducted with men and women with access to a mobile phone (own a phone or have a phone they can use). Those without any phone access are the most socioeconomically marginalized; future research is needed to determine whether these people will enter Cluster 1 as they gain phone access or whether entirely new cluster analysis will be required as phone access becomes universal. Variables related to digital skills required respondents to have a mobile phone during interview. Observations with missing values on those variables were assumed to be for individual who were not able to perform the task. This assumption may result in the decrease in prevalence of digitally skilled people across clusters. Second, there were model limitations: K-Means algorithms only accept numerical inputs. Converting categorical variables into numerical variables using one hot encoding may result in sparse data when the number of categories is higher, consequently K-means is very unlikely to give meaningful clusters when a large set of variables or characteristics are used. In recognition of the challenge related to model limitation, we set a threshold on the number of categories to include, we also invoked principal components analysis for dimensionality reduction.

Conclusions

Study findings sought to identify distinct clusters of husbands and wives based on their sociodemographic, phone access and use characteristics, and to explore the differential impact of a maternal mobile messaging program across these clusters. Three identifiable groups emerge each with differing levels of digital access and use. Descriptive analyses suggest that improvements in some health behaviours were observed for a greater number of outcomes in Cluster 2, than in Clusters 1 and 3. These findings suggest that one size fits all mobile health communications solutions may only engage one segment of a target beneficiary

For peer review only

population, and offer much promise for future direct to beneficiary and other digital health programs which could see greater reach, exposure and impact through differentiated design and implementation. More quantitative and qualitative work is needed to better understand factors driving the differences in impact and what is likely to motivate adoption of target behaviours in different clusters.

Acknowledgments: We thank the women and families of Madhya Pradesh who generously gave of their time to support this work. We are humbled by the opportunity to convey their perspectives and experiences. We additionally are grateful to Dr. Rajani Ved at the National Health Systems Resource Centre for her support. This work was made possible by the Bill and Melinda Gates Foundation. We thank Diva Dhar, Suhel Bidani, Rahul Mullick, Dr. Suneeta Krishnan, Dr. Neeta Goel and Dr. Priya Nanda for believing in us and giving us this opportunity. We additionally wish to thank BBC Media Action teams in India and London for their partnership and collaboration. The evaluation was unquestionably strengthened by their support, transparency, and willingness to work with us on all facets of the research. We too are grateful to the larger team of enumerators from OPM-India who worked tirelessly over many months to implement the surveys that form the backbone of our analyses. We additionally thank Prabal Singh, Vinit Pattnaik at OPM and Alain Labrique, Smisha Agarwal, and Erica Crawford at Johns Hopkins University for their support. Lastly, our figures have been beautified by the great and ever patient Dan Harder of the Creativity Club UK. We thank him for his work.

Contributions: JJHB conducted the analysis and wrote the paper with AEL and inputs from DM, SC, and other authors. AEL is the overall study PI, helped to secure the funding, led the design of the study tools, supported oversight of field work and analysis, and wrote the manuscript with JJHB and DM. DM helped to secure funding, helmed the study design including sampling and randomisation, helped draft study tools, provided input to data analysis, and edited the manuscript. SC helped to secure the funding, draft and review study tools, interpret data analyses and study findings, and edit the manuscript. AG, KS, helped to draft and review study tools, interpret data analyses and study findings, and edit the manuscript. NM is the UCT study PI and provided input to study design, oversight to the analysis and interpretation, and edited the manuscript.

Competing interests: All authors have completed the Unified Competing Interest form (available on request from the corresponding author) and declare that the research reported was funded by the Bill and Melinda Gates Foundation. AG and SC are employed by BBC Media Action; one of the entities supporting program implementation. The authors do not have other relationships and are not engaged in activities that could appear to have influenced the submitted work.

Funding: Bill and Melinda Gates Foundation

Data sharing: The anonymised raw data underpinning analyses presented will be uploaded at the time of publication as a supplementary file.

References

[1] M. Deshmukh, P.J.W. Mechael, DC: mHealth Alliance, Addressing gender and women's empowerment in mHealth for MNCH: An analytical framework, (2013).

For peer review only

[2] N.U.Z. Khan, S. Rasheed, T. Sharmin, A. Siddique, M. Dibley, A.J.B.h.s.r. Alam, How can mobile phones be used to improve nutrition service delivery in rural Bangladesh?, 18(1) (2018) 1-10.
[3] S. Lund, M. Hemed, B.B. Nielsen, A. Said, K. Said, M. Makungu, V.J.B.A.I.J.o.O. Rasch, Gynaecology, Mobile phones as a health communication tool to improve skilled attendance at delivery in Zanzibar: a cluster-randomised controlled trial, 119(10) (2012) 1256-1264.
[4] M. Njoroge, D. Zurovac, E.A. Ogara, J. Chuma, D.J.B.r.n. Kirigia, Assessing the feasibility of eHealth and mHealth: a systematic review and analysis of initiatives implemented in Kenya, 10(1) (2017) 1-11.
[5] J.J.H. Bashingwa, D. Mohan, S. Chamberlain, S. Arora, J. Mendiratta, S. Rahul, V. Chauhan, K. Scott, N. Shah, O.J.B.G.H. Ummer, Assessing exposure to Kilkari: a big data analysis of a large maternal mobile messaging service across 13 states in India, 6(Suppl 5) (2021) e005213.
[6] J.J.H. Bashingwa, N. Shah, D. Mohan, K. Scott, S. Chamberlain, N. Mulder, S. Rahul, S. Arora, A. Chakraborty, O.J.B.g.h. Ummer, Examining the reach and exposure of a mobile phone-based training programme for frontline health workers (ASHAs) in 13 states across India, 6(Suppl 5) (2021) e005299.
[7] A. LeFevre, S. Chamberlain, N. Singh, K. Scott, P. Menon, P. Barron, R. Ved, A. George, Avoiding the Road to Nowhere: Policy Insights on Scaling up and Sustaining Digital Health, Global Policy (2021).
[8] A. Swartz, A.E. LeFevre, S. Perera, M.V. Kinney, A.S. George, Multiple pathways to scaling up and sustainability: an exploration of digital health solutions in South Africa, Global Health 17(1) (2021) 77.
[9] GSMA, Connected women: The mobile gender gap report 2020, GSM Association (2020).
[10] A.E. LeFevre, N. Shah, J.J.H. Bashingwa, A.S. George, D. Mohan, Does women's mobile phone ownership matter for health? Evidence from 15 countries, BMJ Glob Health 5(5) (2020).
[11] D. Mohan, J.J.H. Bashingwa, N. Tiffin, D. Dhar, N. Mulder, A. George, A.E. LeFevre, Does having a mobile phone matter? Linking phone access among women to health in India: An exploratory analysis of the National Family Health Survey, PLoS One 15(7) (2020) e0236078.
[12] K. Scott, O. Ummer, A. Shinde, M. Sharma, S. Yadav, A. Jairath, N. Purty, N. Shah, D. Mohan, S.J.B.G.H. Chamberlain, Another voice in the crowd: the challenge of changing family planning and child feeding practices through mHealth messaging in rural central India, 6(Suppl 5) (2021) e005868.
[13] D. Mohan, J.J.H. Bashingwa, P. Dane, S. Chamberlain, N. Tiffin, A.J.J.r.p. Lefevre, Use of big data and machine learning methods in the monitoring and evaluation of digital health programs in India: An exploratory protocol, 8(5) (2019) e11456.
[14] A. LeFevre, N. Shah, K. Scott, S. Chamberlain, O. Ummer, J.J.H. Bashingwa, A. Chakraborty, A. Godfrey, P. Dutt, D. Mohan, Are stage-based, direct to beneficiary mobile communication programs effective in improving maternal newborn and child health outcomes in India? Results from an individually randomised controlled trial of a national programme, BMJ Global Health, In press (2021).
[15] A. LeFevre, S. Agarwal, S. Chamberlain, K. Scott, A. Godfrey, R. Chandra, A. Singh, N. Shah, D. Dhar, A. Labrique, A. Bhatnagar, D. Mohan, Are stage-based health information messages effective and good value for money in improving maternal newborn and child health outcomes in India? Protocol for an individually randomized controlled trial, Trials 20(1) (2019) 272.

For peer review only

[16] I.I.f.P. Sciences, National Family Health Survey 2015-2016 State Fact Sheet Madhya Pradesh. Mumbai: International Institute for Population Sciences, Government of India, Ministry of Health and Family Welfare; 2016.
[17] A. LeFevre, N. Shah, K. Scott, S. Chamberlain, O. Ummer, J.J. Bashingwa, A. Chakraborty, R. Ved, D. Mohan, Are stage-based mobile health information messages effective in improving maternal newborn and child health outcomes in India? Results from an individually randomized controlled trial Submitted Lancet GH (2021).
[18] B. Dash, D. Mishra, A. Rath, M.J.I.J.o.E. Acharya, Science, Technology, A hybridized K-means clustering approach for high dimensional dataset, 2(2) (2010) 59-66.
[19] C. Ding, X. He, H. Zha, H.D. Simon, Adaptive dimension reduction for clustering high dimensional data, 2002 IEEE International Conference on Data Mining, 2002. Proceedings., IEEE, 2002, pp. 147-154.
[20] S.J.a.p.a. Dasgupta, Experiments with random projection, (2013).
[21] L. Parsons, E. Haque, H.J.A.s.e.n. Liu, Subspace clustering for high dimensional data: a review, 6(1) (2004) 90-105.
[22] C. Ding, T. Li, Adaptive dimension reduction using discriminant analysis and k-means clustering, Proceedings of the 24th international conference on Machine learning, 2007, pp. 521-528.
[23] R. Muthukrishnan, R. Rohini, LASSO: a feature selection technique in predictive modeling for machine learning, 2016 IEEE international conference on advances in computer applications (ICACA), IEEE, 2016, pp. 18-20.
[24] M. Yamada, W. Jitkrittum, L. Sigal, E.P. Xing, M.J.N.c. Sugiyama, High-dimensional feature selection by feature-wise kernelized lasso, 26(1) (2014) 185-207.
[25] A. Likas, N. Vlassis, J.J.J.P.r. Verbeek, The global k-means clustering algorithm, 36(2) (2003) 451-461.
[26] T.M. Kodinariya, P.R.J.I.J. Makwana, Review on determining number of Cluster in K-Means Clustering, 1(6) (2013) 90-95.
[27] C. Genolini, X. Alacoque, M. Sentenac, C.J.J.o.S.S. Arnaud, kml and kml3d: R packages to cluster longitudinal data, 65(4) (2015) 1-34.
[28] M. Liao, Y. Li, F. Kianifard, E. Obi, S.J.B.n. Arcona, Cluster analysis and its application to healthcare claims data: a study of end-stage renal disease patients who initiated hemodialysis, 17(1) (2016) 1-14.
[29] C. Violán, A. Roso-Llorach, Q. Foguet-Boreu, M. Guisado-Clavero, M. Pons-Vigués, E. Pujol-Ribera, J.M.J.B.f.p. Valderas, Multimorbidity patterns with K-means nonhierarchical cluster analysis, 19(1) (2018) 1-11.
[30] Z. Che, Y. Cheng, S. Zhai, Z. Sun, Y. Liu, Boosting deep learning risk prediction with generative adversarial networks for electronic health records, 2017 IEEE International Conference on Data Mining (ICDM), IEEE, 2017, pp. 787-792.
[31] T.M. Santos, B.O. Cata-Preta, C.G. Victora, A.J.J.V. Barros, Finding Children with High Risk of Non-Vaccination in 92 Low-and Middle-Income Countries: A Decision Tree Approach, 9(6) (2021) 646.

For peer review only

Figure 1. Framework for segmentation analysis
Figure 2. Elbow method used to help decide ultimate number of clusters appropriate for the data.
Figure 3. Silhouette analysis for three and four clusters
Figure 4. Distribution of select characteristics with strong signals by Cluster.
Variables which had at least a prevalence of 70% in one or more clusters and differed from another cluster by 50% or more were considered to have a strong signal (*Reported by men interviewed, **Observed by survey enumerators)

For peer review only

Figure 1. Framework for segmentation analysis.

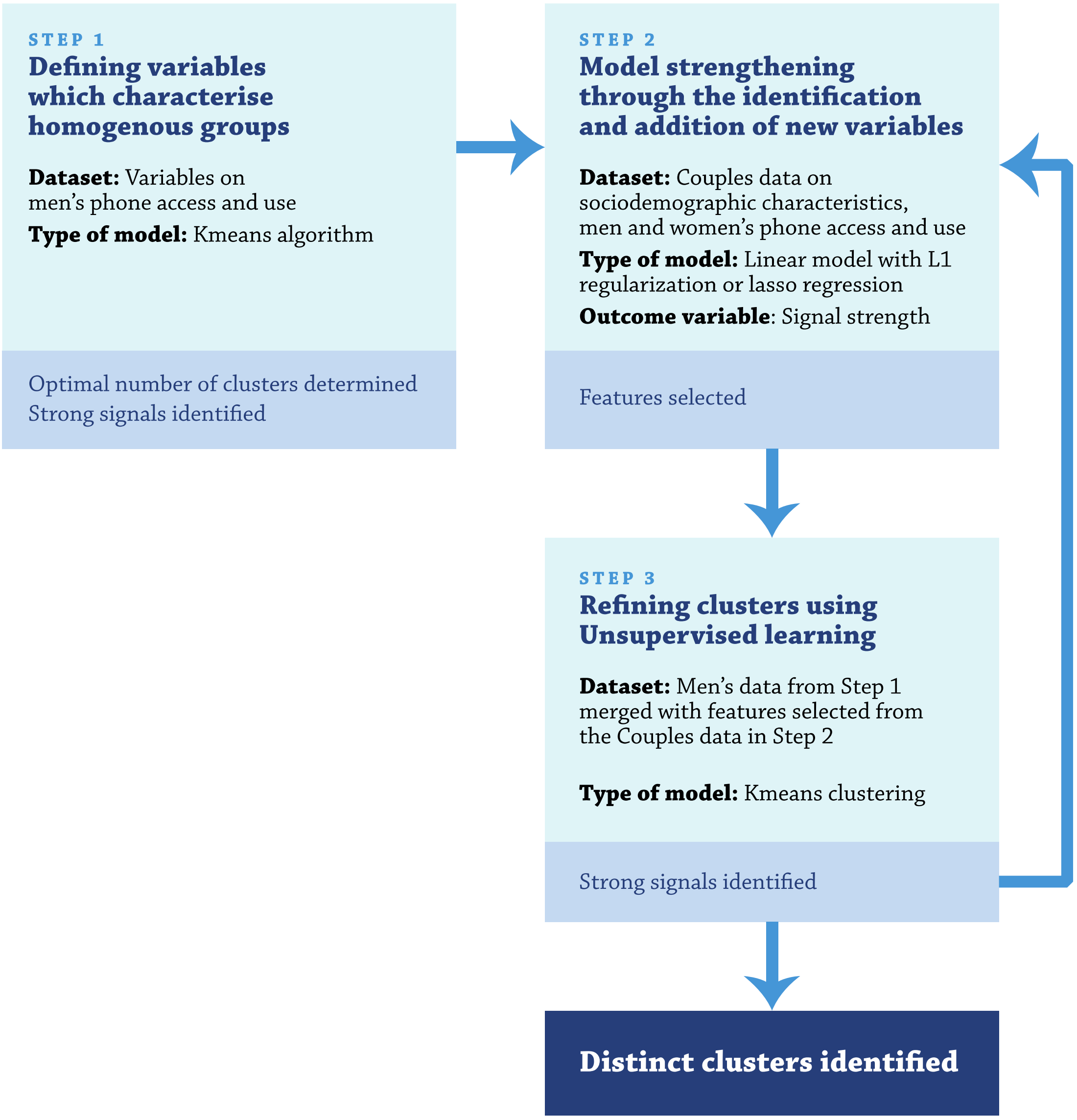

Figure 2. Elbow method used to help decide ultimate number of clusters appropriate for the data.

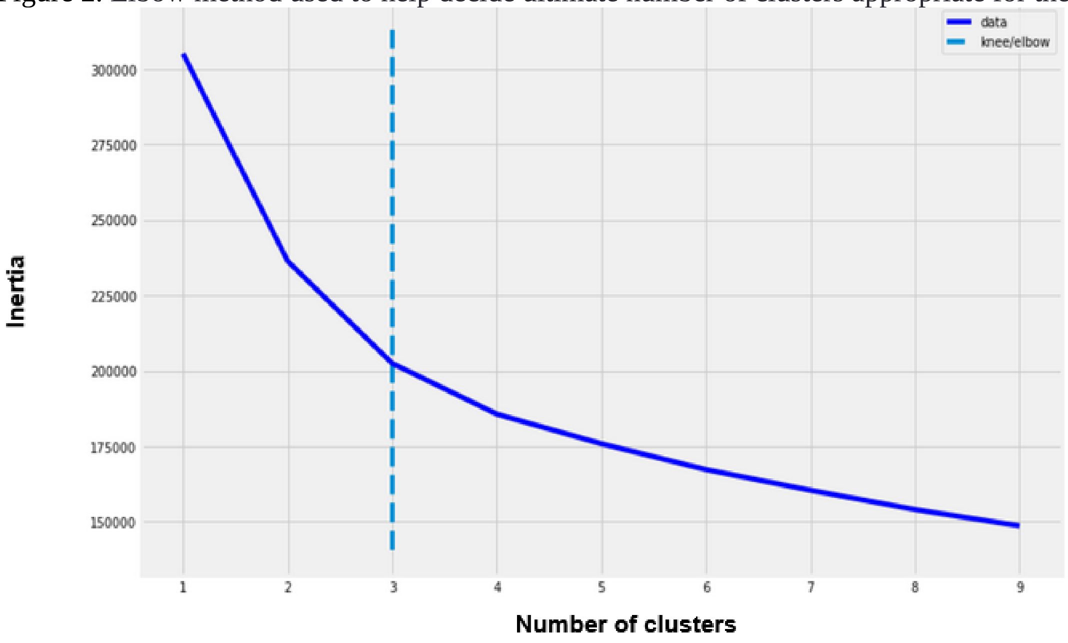

Figure 3. Silhouette analysis for three and four clusters

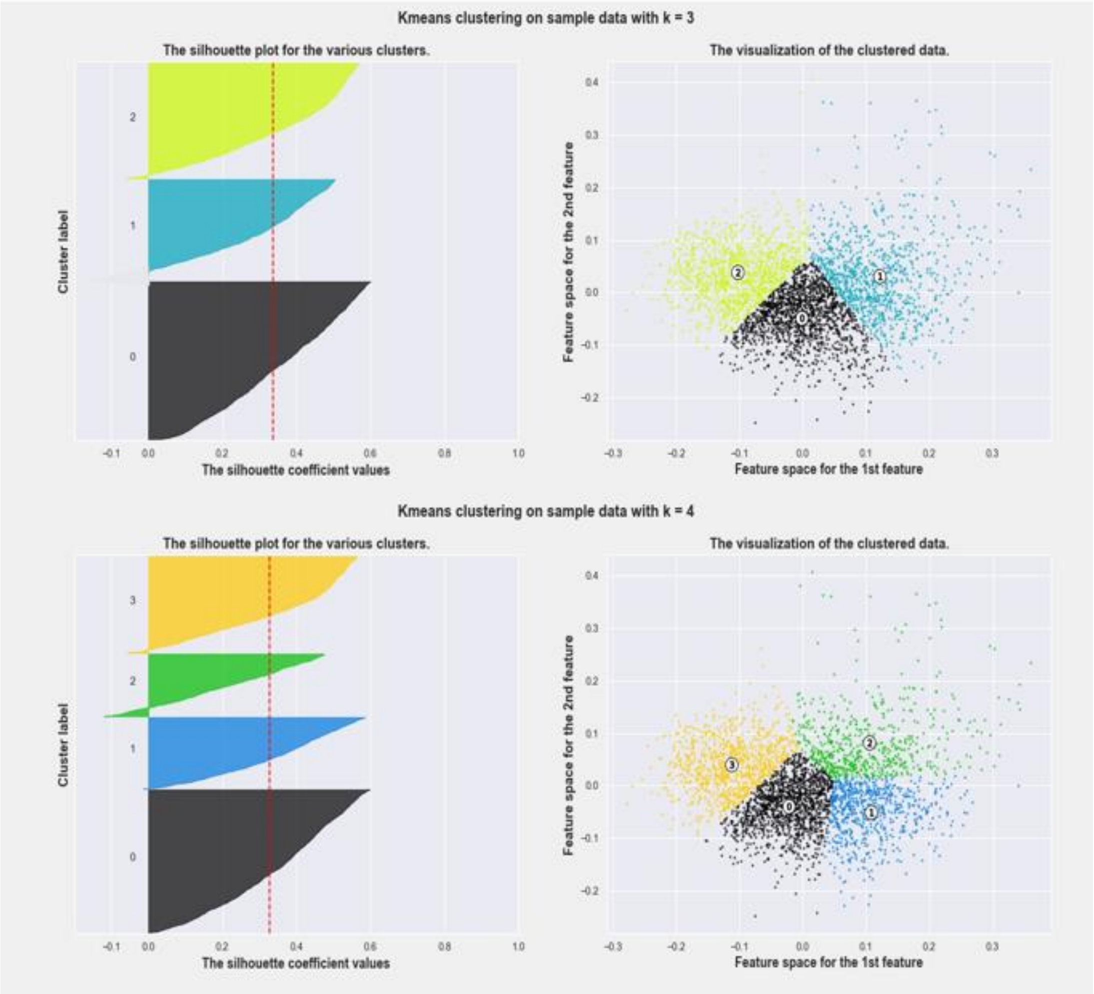

Figure 4. Distribution of select characteristics with strong signals by Cluster. Variables which had at least a prevalence of 70% in one or more clusters and differed from another cluster by 50% or more were considered to have a strong signal.

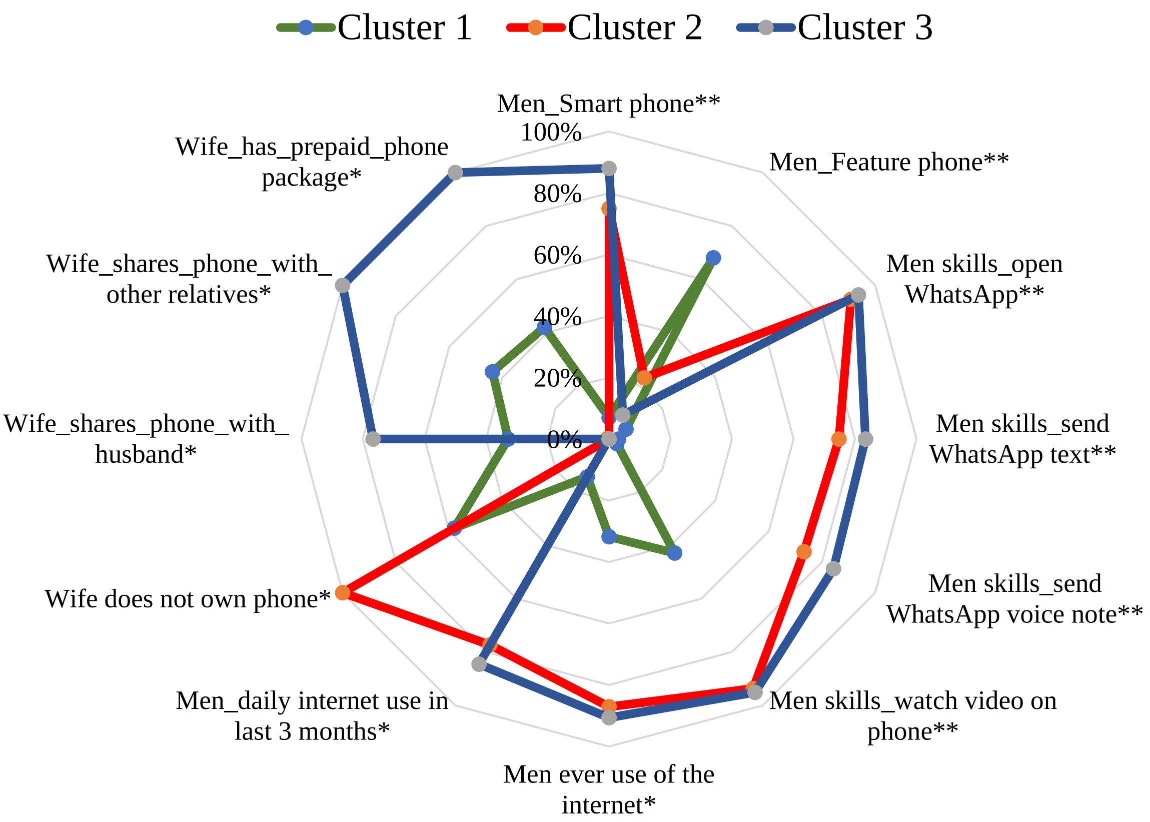

*Reported by men interviewed
**Observed by survey enumerators

For peer review only

Supplementary Table1. Study sample characteristics (variables used as starting point for couple's survey data)

	Women's survey		Men's survey	
**Variables**	N	%	N	%
**Education**				
0-5 years	610	18	586	17
>5 years	2874	82	2898	83
**District**				
Hoshangabad	345	10	345	10
Mandsaur	676	19	676	19
Rajgarh	791	23	791	23
Rewa	1672	48	1672	48
**Ethnicity/Caste**				
General	780	22	698	20
OBC	1690	49	1738	50
Scheduled caste	647	19	690	20
Scheduled tribe	345	10	357	10
**Age at time of enrollment in years**				
18-24	2027	58	564	16
25-34	1391	40	2477	71
35+	66	2	443	13
**Education**				
Never been to school	347	10	100	3
Primary school or less	610	18	586	17
Middle school	1042	30	932	27
High school	1168	34	1322	38
Higher education	317	9	544	16
**MNO**				
Airtel	893	26	791	23
Idea	1572	45	967	28
Jio	229	7	1270	36
Tata	9	0	4	0
vodafone	781	22	427	12
BSNL			24	1
**Frequency of most recent top up**				
More than 3 months	299	9		
Within 1 month	1626	47		

Within 1 week	718	21		
Within 3 months	841	24		
**Who topped up credit**				
Husband	2784	80		
Other	357	10		
self	343	10		
**Who taught respondent how to use phone**				
Husband	794	23		
Other	178	5		
Self	2512	72		
**Permission for wife's phone use**				
Wife takes permission to make call	1133	33		
Wife takes permission before picking up call	1614	46		
Wife takes permission to recharge	838	24		
Women need oversight to use phone	2514	72		
**Type of phone**				
Brick phone	454	13	357	10
Feature phone	2206	63	1234	35
Smart phone	824	24	1838	53
**Use phone to call spouse**	2563	74	2926	84
**Use phone to call ASHAs**	293	8	2478	71
**Use phone for internet**	1	0	1417	41
**Use phone to listen radio**	1	0	1868	54
**Observe phone**				
Phone working	2820	81	3251	93
**Digital Tasks**				
Able to navigate IVR prompts	2995	86	3319	95
Give a missed call	2409	69	2890	83
Store contacts on phone	2845	82	2999	86
Open SMS	1654	47	2966	85
Read SMS	1102	32	2188	63
Overall digital literacy	937	27	1938	56
Open and read SMS	1102	32	2188	63
**Involvement in Decision making**				
About daily household expenditures	713	20	2065	59
About big expenditures	623	18	2243	64

About health during pregnancy	937	27	3081	88
**Employment status**	1398	40	3458	99
**Socio-economic status**				
Poorest	542	16	542	16
Poorer	646	19	646	19
Middle	710	20	710	20
Richer	760	22	760	22
Richest	826	24	826	24
**Phone in the household**				
1	759	22	759	22
2	1437	41	1437	41
>2	1288	37	1288	37
**Parity**				
No child	1406	40	1406	40
One child	1256	36	1256	36
Two and more	822	24	822	24
**Religion**				
Hindu	3297	95	3297	95
Muslim	183	5	183	5
Other	4	0	4	0
**Frequency of phone use in last 3 months**				
Every day	2700	77		
not every day	784	23		
**Age at marriage**				
0-15 years	416	12		
>15 years	3068	88		

For peer review only - http://bmjopen.bmj.com/site/about/guidelines.xhtml

Supplementary Table 2. Metrics used for cluster validation (Davies-Bouldin and Calinski-Harabatz criterions have been normalized to [0,1] ,1 indicating a good partition)

Number of clusters	**Within cluster sum of square**	**Silhouette index**	**Ray -Turi index**	**Calinski – Harabatz index**
**2**	64791,07	0,812424	0,873942	0,820123
**3**	62595,37	0,801119	1	0,9563
**4**	60983,52	0,509252	0,853942	0,360082
**5**	59662,45	0,466859	0,529231	0,243941
**6**	58571,27	0,454165	0,482203	0,161834
**7**	57686,73	0,420884	0,427094	0,096974
**8**	56943,46	0,402445	0,249373	0,044445
**9**	56322,05	0,386873	0,268434	0

Table 3a. Men's sample characteristics by cluster based on Men's survey data from four districts of Madhya Pradesh

	**Total** n=3,484		**Cluster 1** n=1,408		**Cluster 2** n=666		**Cluster 3** n=1,410	
	%	n	%	n	%	n	%	n
**Sociodemographic characteristics**								
**Caste**								
General	20	698	15	208	17	112	27	378
OBC	50	1 738	45	637	50	334	54	767
Scheduled tribe	10	357	15	213	11	73	5	71
Scheduled caste	20	690	25	350	22	146	14	194
**Education**								
Never been to school	3	100	7	92	1	6	-	2
Primary school or less	17	586	29	403	13	84	7	99
Middle school	27	932	32	446	28	189	21	297
High school	38	1 322	29	415	42	280	44	627
Higher education	16	544	4	52	16	107	27	385
**Number of phones in the household**								
0-1	22	759	34	476	24	157	9	126
2	41	1 437	45	629	43	284	37	524
3+	37	1 288	22	303	34	225	54	760

**Phone ownership and sharing**								
Own phone and do not share	17	578	16	221	8	50	22	307
Own phone and do share	78	2 730	73	1 031	91	607	77	1 092
Share only	3	93	5	73	1	9	1	11
**Phone type (observed)**								
Brick phone	10	357	22	304	3	17	3	36
Feature phone	35	1 234	68	953	23	151	9	130
Smart phone	53	1 838	7	96	75	498	88	1 244
**Men's phone use**								
Daily phone use (reported)	95	3 327	89	1 260	99	662	100	1 405
**Phone features used (reported)**								
Calls	98	3 422	96	1 350	100	666	100	1 406
SMS	46	1 615	19	263	55	369	70	983
WhatsApp	61	2 109	7	97	95	635	98	1 377
Watch video	80	2 784	52	726	99	659	99	1 399
Share video	58	2 008	6	87	89	591	94	1 330
Make video	35	1 209	9	121	47	316	55	772
Download Apps	47	1 640	2	29	70	468	81	1 143
Music	86	2 984	68	959	97	649	98	1 376
Radio	26	889	14	200	32	210	34	479
Search Google	55	1 925	9	128	82	548	89	1 249
Search YouTube	67	2 327	21	300	98	653	97	1 374
Camera	84	2 921	61	857	99	659	100	1 405
Share photo	59	2 039	7	93	90	602	95	1 344
Mobile money	16	560	0	3	15	103	32	454
Transfer mobile money	13	463	0	1	12	82	27	380
Transfer mobile credit	13	459	0	1	12	83	27	375
**Men's Digital skills (observed)**								
Able to navigate IVR prompts	95	3 319	91	1 280	98	656	98	1 383
Give a missed call	83	2 890	72	1 020	88	588	91	1 282
Store contacts on phone	86	2 999	73	1 031	94	623	95	1 345
Open SMS	85	2 966	71	994	94	624	96	1 348
Read SMS	63	2 188	38	530	73	483	83	1 175
Overall Basic Digital Skill Level	56	1 938	29	415	65	432	77	1 091
**WhatsApp skills (observed)**								
Open WhatsApp	58	2 017	6	91	91	605	94	1 321
Send WhatsApp text	49	1 718	3	44	75	498	83	1 176
Send WhatsApp voice note	49	1 719	3	42	73	488	84	1 189
**Watch video on phone (observed)**	74	2 568	43	603	94	624	95	1 341
**Men report getting images and videos from**								

Internet: YouTube	59	2 062	19	274	83	554	88	1 234
Internet: Google	45	1 569	9	130	64	429	72	1 010
Other relatives	36	1 249	4	63	54	360	59	826
Friends locally	55	1 916	11	153	83	550	86	1 213
Friends other states	25	885	1	21	36	238	44	626
**Computer/ tablet ownership and use**								
Own Computer/ tablet	6	220	1	13	4	28	13	179
Daily computer / tablet use	5	184	0	3	5	30	11	151
Ever use of the internet from any device/ location (reported)	66	2 305	32	447	87	580	91	1 278
Daily internet use in last 3 months (reported)	55	1 906	14	199	77	515	85	1 192
**Wife owns phone**	57	3 484	42	591	-	-	100	1 410
**Wife's phone type**								
Brick phone	10	363	10	134	0	1	16	228
Feature phone	29	1 016	27	375	-	-	45	641
Smart phone	19	647	8	106	-	-	38	541
**Wife shares phone with**								
Husband	44	1 543	33	461	-	-	77	1 082
Children (male or female)	5	180	4	52	-	-	9	128
Parents in law	9	329	6	83	-	-	17	246
Wife's parents	3	107	2	33	-	-	5	74
Other relatives	58	2 028	44	615	0	3	100	1 410
Friend/ neighbour	1	30	1	9	-	-	1	21
**Phone features wife uses (reported)**								
Calls: receive, dial, or speak	100	3 475	100	1 404	100	663	100	1 408
SMS	33	1 146	16	228	28	185	52	733
WhatsApp	35	1 225	11	155	38	255	58	815
Watch shows	54	1 871	26	368	68	450	75	1 053
Music or radio	100	3 484	100	1 408	100	666	100	1 410
Search internet	34	1 192	12	168	36	240	56	784
Camera	74	2 589	55	772	84	559	89	1 258
**Men's perceptions about restrictions (if any) which should be placed on phone use**								
**No restrictions should be placed on adult phone use**	86	2 992	85	1 192	86	571	87	1 229
**Oversight needed for**								
Men	47	1 647	54	767	46	307	41	573
Women	72	2 514	79	1 114	71	476	66	924
Male children	82	2 863	86	1 207	79	523	80	1 133
Female children	92	3 198	93	1 311	91	608	91	1 279
**Men report that their wife needs their permission to pick up**								

**calls from**								
Someone unknown	46	1 614	46	653	51	341	44	620
Family	13	461	17	237	18	122	7	102
Friends/ Neighbours	32	1 121	35	488	41	274	25	359
Health workers	22	757	25	356	29	195	15	206
Business associates	28	990	29	410	35	232	25	348
**Men report women need their permission to make a call to**								
Family	17	600	21	293	24	162	10	145
Friends/ Neighbours	21	735	25	345	28	187	14	203
Health workers	20	692	22	315	29	192	13	185
Business associates	14	484	17	236	16	109	10	139
Unknown to husband	17	608	20	286	20	134	13	188
**Men report women need their permission to send SMS or WhatsApp to**								
Family	2	72	1	12	4	28	2	32
Friends/ Neighbours	3	101	1	12	6	41	3	48
Health workers	2	77	1	9	5	30	3	38
Business associates	2	54	1	11	3	18	2	25
Unknown to husband	3	100	1	13	5	35	4	52
**Man has concerns about wife's phone ownership or use**	1	24	1	10	2	11	0	3
**Reasons for concern (multi-select):**								
Cost of phone	0	3	0	1	0	2	-	-
Cost of using phone	0	9	0	4	0	2	0	3
Reputational risk	0	13	0	5	1	8	-	-
Relationships with other men	0	3	0	2	0	1	-	-
Bad friendships with other women	0	3	0	1	0	2	-	-
Financially defrauded	0	1	-	-	0	1	-	-
**Men would like their wives to use the mobile phone to**								
Transfer money	41	1 439	30	423	42	281	52	735
Buy/ pay for things	37	1 304	26	368	38	256	48	680

Table 3b. Women's sample characteristics by cluster based on women's baseline survey data from four districts of Madhya Pradesh

	Total n=3,484		Cluster 1 n=1,408		Cluster 2 n=666		Cluster 3 n=1,410	
	%	n	%	n	%	n	%	n
**Sociodemographic characteristics**								
**Socioeconomic status**								
Poorest	16	542	26	369	13	88	6	85
Poorer	19	646	27	379	18	117	11	150
Middle	20	710	22	313	25	167	16	230
Richer	22	760	15	214	25	165	27	381
Richest	24	826	9	133	19	129	40	564
**District**								
Hoshangabad	10	345	11	151	11	76	8	118
Mandsaur	19	676	13	181	14	95	28	400
Rajgarh	23	791	21	302	29	191	21	298
Rewa	48	1 672	55	774	46	304	42	594
**Mean age (years)**	72	3 484	25	1 408	23	666	24	1 410
**Ethnicity/Caste**								
General	22	780	17	242	19	129	29	409
OBC	49	1 690	45	628	48	321	53	741
Scheduled caste	19	647	23	322	21	140	13	185
Scheduled tribe	10	345	14	203	11	72	5	70
**Education**								
Never been to school	10	347	16	229	8	50	5	68
Primary school or less	18	610	23	327	17	114	12	169
Middle school	30	1 042	32	451	35	236	25	355
High school	34	1 168	26	363	33	223	41	582
Higher education	9	317	3	38	6	43	17	236
**Phone ownership and sharing**								
Own phone and do not share	51	1 781	43	609	38	256	65	916
Own phone and share	22	772	23	318	22	145	22	309
Share only	26	923	34	475	40	264	13	184
**Phone type (observed)**								
Brick phone	7	248	8	113	8	50	6	85
Feature phone	63	2 206	74	1 040	54	359	57	807
Smart phone	24	824	11	158	28	188	34	478
No phone observed	6	206	7	97	10	69	3	40
**Women's phone characteristics**								
**Phone features (observed)**								
Call	79	2 765	76	1 072	71	470	87	1 223

Speaker	79	2 762	76	1 072	71	470	87	1 220
SMS	79	2 768	76	1 074	71	471	87	1 223
Contacts	79	2 766	76	1 072	71	471	87	1 223
Camera	66	2 302	63	889	60	398	72	1 015
Music/ audio content	69	2 419	66	923	63	419	76	1 077
Internet	49	1 712	42	596	47	312	57	804
Bluetooth	64	2 243	60	842	59	390	72	1 011
Radio/FM	69	2 416	64	907	62	415	78	1 094
**Applications installed on phone (observed)**								
Facebook	25	859	17	237	23	156	33	466
WhatsApp	17	603	8	113	18	117	26	373
Shareit	10	364	4	61	11	71	16	232
**Proportion of phones with zero balance at time of interview**	48	1 666	47	655	50	334	48	677
**Who topped up credit?**								
Husband	80	2 784	79	1 109	81	537	81	1 138
Self	10	357	11	157	12	79	9	121
Other	10	343	10	142	8	50	11	151
**Frequency of most recent top-up**								
Within 1 week	21	718	24	343	19	125	18	250
Within 1 month	47	1 626	46	645	46	309	48	672
Within 3 months	24	841	21	299	23	155	27	387
More than 3 months	9	299	9	121	12	77	7	101
**Total amount of last top up**								
>50	55	1 902	59	831	47	311	54	760
0-50	45	1 582	41	577	53	355	46	650
**Women's phone use**								
**Digital skill (observed)**								
Able to navigate IVR prompts	69	2 409	81	1 142	87	578	90	1 275
Give a missed call	82	2 845	64	895	60	401	79	1 113
Store contacts on phone	47	1 654	73	1 021	83	555	90	1 269
Open SMS	32	1 102	33	471	39	263	65	920
Read SMS	32	1 102	18	255	26	171	48	676
Overall Basic Digital Skill Level	27	937	15	213	21	139	41	585
**Communication**	74	2 563	65	917	68	455	84	1 191
Call with spouse	73	2 542	81	905	80	454	89	1 183
Call with friends, relatives	43	1 485	83	478	87	297	82	710
Call with health workers	32	1 132	99	317	99	196	97	619
SMS with husband	16	545	97	103	99	91	96	351

SMS with friends, relatives	9	330	98	45	100	49	100	236
SMS with health workers	6	213	100	27	100	24	99	162
Dialled a number and listened to pre-recorded message	77	2 700	72	1 010	73	489	85	1 201
**Who taught respondent how to use phone?**								
Spouse	5	178	5	72	5	35	5	71
Self	72	2 512	70	986	71	472	75	1 054
Other	23	794	25	350	24	159	20	285

For peer review only

Supplementary Table 4. Strong signals (variable used for the spide charts are highlighted)

	Cluster 1 (n=1408)	Cluster 2 (n=666)	Cluster 3 (n=1410)
**Men paid for wife's balance**	37	0	90
**Men can perform basic internet search**	7	66	77
**Men report that their wife uses prepaid pack**	42	0	100
**Men report that women need their permission to add credit**	18	0	42
**Men report ever use of internet**	31	87	91
**Observe men watching Video**	42	93	95
**Men can send WhatsApp text**	3	77	85
**Men report use of WhatsApp**	7	91	95
**Men report that their wife's use the phone to**			
Search internet	12	36	55
Watch show	26	66	75
WhatsApp	11	37	57
Men report that they can send photo on WhatsApp	4	88	93
Men report that they can send a WhatsApp voice message	3	73	84
**Men report getting images and videos from**			
Internet: YouTube	19	84	88
Internet: Google	9	64	71
Other relatives	4	55	59
Friends locally	11	83	87
Friends other states	2	36	44
**Men report not using the internet frequently**	86	23	15
**Men have smart phone**	6	75	88
**Men report using the internet frequently**	14	77	85
**Men have feature phone**	68	23	9
**Number of phones in the household**			
3+	19	32	61
0-1	43	39	2
**Men report that their wife own's a phone**	42	0	100
**Men report that their wife does not own a phone**	58	100	0
**Men report their wife shares phone she owns with husband**	32	0	77
**Men observed to open WhatsApp**	6	91	94
**Men's observed digital literacy**	29	64	77
**Men observed to read SMS**	37	72	82
**Features men report using on their phone**			
Share photo	7	90	96
Search YouTube	21	98	98
Search Google	9	82	88
Download Apps	2	70	82
Make video	8	48	55
Share video	6	88	94
Watch video	51	99	99
WhatsApp	7	95	98
SMS	18	55	69
**Observe TikTok App on men's phone**	1	36	48
**Men have internet in their household**	25	54	69
**Men report women having a phone other than Samsung or Jio**	24	0	53

For peer review only

**Men report that women have a feature phone**	26	0	46

Supplementary Figure 1. PCA with 95% of cumulative explained variance on couples' data.

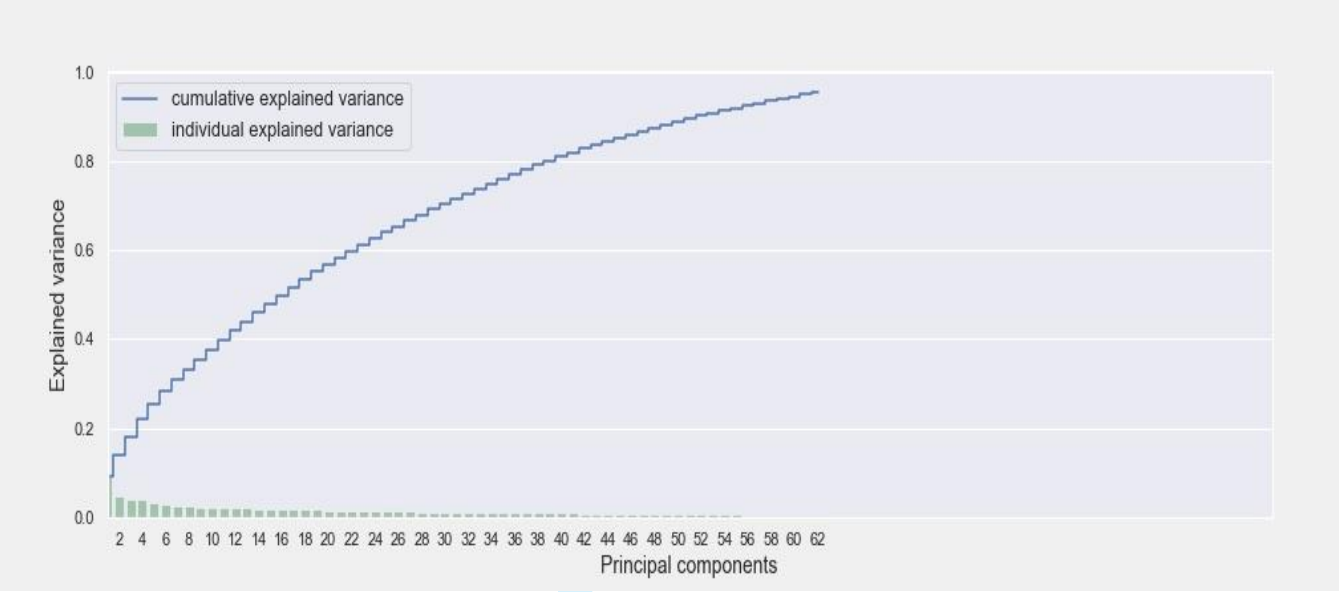

review only

Reporting checklist for quality improvement in health care.

Based on the SQUIRE guidelines.

Instructions to authors

Complete this checklist by entering the page numbers from your manuscript where readers will find each of the items listed below.

Your article may not currently address all the items on the checklist. Please modify your text to include the missing information. If you are certain that an item does not apply, please write "n/a" and provide a short explanation.

Upload your completed checklist as an extra file when you submit to a journal.

In your methods section, say that you used the SQUIREreporting guidelines, and cite them as:

Ogrinc G, Davies L, Goodman D, Batalden P, Davidoff F, Stevens D. SQUIRE 2.0 (Standards for QUality Improvement Reporting Excellence): revised publication guidelines from a detailed consensus process

		Reporting Item	Page Number
**Title**			
	#1	Indicate that the manuscript concerns an initiative to improve healthcare (broadly defined to include the quality, safety,	1

For peer review only

		effectiveness, patientcenteredness, timeliness, cost, efficiency, and equity of healthcare)	
**Abstract**			3
	#02a	Provide adequate information to aid in searching and indexing	3
	#02b	Summarize all key information from various sections of the text using the abstract format of the intended publication or a structured summary such as: background, local problem, methods, interventions, results, conclusions	
**Introduction**			4
Problem description	#3	Nature and significance of the local problem	4
Available knowledge	#4	Summary of what is currently known about the problem, including relevant previous studies	4
Rationale	#5	Informal or formal frameworks, models, concepts, and / or theories used to explain the problem, any reasons or assumptions that were used to develop the intervention(s), and reasons why the intervention(s) was expected to work	4
Specific aims	#6	Purpose of the project and of this report	4
**Methods**			4
Context	#7	Contextual elements considered important at the outset of introducing the intervention(s)	5

For peer review only

Intervention(s)	#08a	Description of the intervention(s) in sufficient detail that others could reproduce it	5
Intervention(s)	#08b	Specifics of the team involved in the work	5
Study of the Intervention(s)	#09a	Approach chosen for assessing the impact of the intervention(s)	6
Study of the Intervention(s)	#09b	Approach used to establish whether the observed outcomes were due to the intervention(s)	6
Measures	#10a	Measures chosen for studying processes and outcomes of the intervention(s), including rationale for choosing them, their operational definitions, and their validity and reliability	6
Measures	#10b	Description of the approach to the ongoing assessment of contextual elements that contributed to the success, failure, efficiency, and cost	7
Measures	#10c	Methods employed for assessing completeness and accuracy of data	7
Analysis	#11a	Qualitative and quantitative methods used to draw inferences from the data	7
Analysis	#11b	Methods for understanding variation within the data, including the effects of time as a variable	7
Ethical considerations	#12	Ethical aspects of implementing and studying the intervention(s) and how they were addressed, including, but	NA

For peer review only

		not limited to, formal ethics review and potential conflict(s) of interest	
**Results**			7
	#13a	Initial steps of the intervention(s) and their evolution over time (e.g., time-line diagram, flow chart, or table), including modifications made to the intervention during the project	7
	#13b	Details of the process measures and outcome	8
	#13c	Contextual elements that interacted with the intervention(s)	8
	#13d	Observed associations between outcomes, interventions, and relevant contextual elements	9
	#13e	Unintended consequences such as unexpected benefits, problems, failures, or costs associated with the intervention(s).	NA
	#13f	Details about missing data	NA
**Discussion**			
Summary	#14a	Key findings, including relevance to the rationale and specific aims	10
Summary	#14b	Particular strengths of the project	10
Interpretation	#15a	Nature of the association between the intervention(s) and the outcomes	10
Interpretation	#15b	Comparison of results with findings from other publications	11

For peer review only

Interpretation	#15c	Impact of the project on people and systems	11
Interpretation	#15d	Reasons for any differences between observed and anticipated outcomes, including the influence of context	11
Interpretation	#15e	Costs and strategic trade-offs, including opportunity costs	11
Limitations	#16a	Limits to the generalizability of the work	11
Limitations	#16b	Factors that might have limited internal validity such as confounding, bias, or imprecision in the design, methods, measurement, or analysis	11
Limitations	#16c	Efforts made to minimize and adjust for limitations	11
Conclusion	#17a	Usefulness of the work	
Conclusion	#17b	Sustainability	11
Conclusion	#17c	Potential for spread to other contexts	12
Conclusion	#17d	Implications for practice and for further study in the field	12
Conclusion	#17e	Suggested next steps	12
**Other information**			12
Funding	#18	Sources of funding that supported this work. Role, if any, of the funding organization in the design, implementation, interpretation, and reporting	2

For peer review only

None The SQUIRE 2.0 checklist is distributed under the terms of the Creative Commons Attribution License CC BY-NC 4.0. This checklist can be completed online using https://www.goodreports.org/, a tool made by the EQUATOR Network in collaboration with Penelope.ai

For peer review only

BMJ Open

Can we design the next generation of digital health communication programs by leveraging the power of artificial intelligence to segment target audiences, bolster impact, and deliver differentiated services? A machine learning analysis of survey data from rural India

Journal:	*BMJ Open*
Manuscript ID	bmjopen-2022-063354.R1
Article Type:	Original research
Date Submitted by the Author:	04-Nov-2022

Complete List of Authors:	Bashingwa, Jean ; University of Cape Town Faculty of Health Sciences,<br>Mohan, Diwakar; Johns Hopkins University Bloomberg School of Public Health<br>Chamberlain, Sara; BBC Media Action, BBC Media Action, India; BBC Media Action, Asia<br>Scott, Kerry; Johns Hopkins University Bloomberg School of Public Health;<br>Ummer, Osama; Oxford Policy Management, ; BBC Media Action,<br>Godfrey, Anna; BBC Media Action,<br>Mulder, Nicola; University of Cape Town<br>Moodley, Deshen; University of Cape Town, Department of Computer Science<br>LeFevre, Amnesty; Johns Hopkins University, International Health

**Primary Subject Heading**:	Public health
Secondary Subject Heading:	Health services research
Keywords:	Public health < INFECTIOUS DISEASES, HEALTH ECONOMICS, Community child health < PAEDIATRICS, Information technology < BIOTECHNOLOGY & BIOINFORMATICS

SCHOLARONE™ Manuscripts

I, the Submitting Author has the right to grant and does grant on behalf of all authors of the Work (as defined in the below author licence), an exclusive licence and/or a non-exclusive licence for contributions from authors who are: i) UK Crown employees; ii) where BMJ has agreed a CC-BY licence shall apply, and/or iii) in accordance with the terms applicable for US Federal Government officers or employees acting as part of their official duties; on a worldwide, perpetual, irrevocable, royalty-free basis to BMJ Publishing Group Ltd ("BMJ") its licensees and where the relevant Journal is co-owned by BMJ to the co-owners of the Journal, to publish the Work in this journal and any other BMJ products and to exploit all rights, as set out in our licence.

The Submitting Author accepts and understands that any supply made under these terms is made by BMJ to the Submitting Author unless you are acting as an employee on behalf of your employer or a postgraduate student of an affiliated institution which is paying any applicable article publishing charge ("APC") for Open Access articles. Where the Submitting Author wishes to make the Work available on an Open Access basis (and intends to pay the relevant APC), the terms of reuse of such Open Access shall be governed by a Creative Commons licence – details of these licences and which Creative Commons licence will apply to this Work are set out in our licence referred to above.

Other than as permitted in any relevant BMJ Author's Self Archiving Policies, I confirm this Work has not been accepted for publication elsewhere, is not being considered for publication elsewhere and does not duplicate material already published. I confirm all authors consent to publication of this Work and authorise the granting of this licence.

For peer review only

Can we design the next generation of digital health communication programs by leveraging the power of artificial intelligence to segment target audiences, bolster impact, and deliver differentiated services? A machine learning analysis of survey data from rural India

Jean Juste Harrisson Bashingwa, PhD (corresponding author)
MRC/Wits-Agincourt Unit, School of Public Health, University of the Witwatersrand, 27 St. Andrews Road, Parktown, 2193, South Africa
Email: jeanjuste@aims.ac.za

Diwakar Mohan, DrPH
Department of International Health, Johns Hopkins Bloomberg School of Public Health, 615 N. Wolfe St, Baltimore, Maryland, USA
Email: dmohan3@jhu.edu

Sara Chamberlain, MA
Innov8 Old Fort Saket District Mall, Saket District Centre, Sector 6, Pushp Vihar, New Delhi, Delhi 110017, India
Email: sara.chamberlain@in.bbcmediaaction.org

Kerry Scott, PhD
Department of International Health, Johns Hopkins Bloomberg School of Public Health, 615 N. Wolfe St, Baltimore, Maryland, USA
Email: kscott26@jhu.edu

Osama Ummer, MHA
(1) BBC Media Action-India, Innov8 Old Fort Saket District Mall, Saket District Centre, Sector 6, Pushp Vihar, New Delhi, Delhi 110017, India
(2) Oxford Policy Management-Delhi, 4/6 First Floor, Siri Fort Institutional Area, New Delhi, Delhi 110049, India
Email: kposamaummer@gmail.com

Anna Godfrey, PhD
BBC Media Action, Ibex House, 42-47 Minories, London, EC3N 1DY, England
Email: anna.godfrey@bbc.co.uk

Nicola Mulder, PhD
Computational Biology Division, Department of Integrative Biomedical Sciences, Institute of Infectious Disease and Molecular Medicine, Faculty of Health Sciences, University of Cape Town
Anzio Road, Observatory, 7925, Cape Town, South Africa
Email: nicola.mulder@uct.ac.za

Deshen Moodley, PhD
Department of Computer Science,
18 University Avenue, University of Cape Town
Rondebosch, Cape Town, South Africa
Email: deshen@cs.uct.ac.za

For peer review only

Amnesty E. LeFevre PhD
1. School of Public Health and Family Medicine, University of Cape Town, Falmouth Rd, Observatory, Cape Town, 7925, South Africa
2. Department of International Health, Johns Hopkins Bloomberg School of Public Health, 615 N. Wolfe St, Baltimore, Maryland, USA
Email: aelefevre@gmail.com

For peer review only

Abstract (268 of 300 words)
Objectives
Direct to beneficiary (D2B) mobile health communication programs have been used to provide reproductive, maternal, neonatal and child health (RMNC) information to women and their families in a number of countries globally. Programs to date have provided the same content, at the same frequency, using the same channel to large beneficiary populations. This manuscript presents a proof of concept approach that uses machine learning to segment populations of women with access to phones and their husbands into distinct clusters to support differential digital program design and delivery.
Setting
Data used in this study were drawn from cross-sectional survey conducted in four districts of Madhya Pradesh, India.
Participants
Study participant included pregnant women with access to a phone (n=5,095) and their husbands (n=3,842)
Results
We used an iterative process involving K-means clustering and Lasso regression to segment couples into three distinct clusters. Cluster 1 (n=1,408) tended to be poorer, lessor educated men and women, with low levels of digital access and skills. Cluster 2 (n=666) had a mid-level of digital access and skills among men but not women. Cluster 3 (n=1,410) had high digital access and skill among men and moderate access and skills among women. Exposure to the D2B program 'Kilkari' showed the greatest difference in Cluster 2, including an 8% difference in use of reversible modern contraceptives, 7% in child immunisation at 10 weeks, 3% in child immunisation at 9 months, and 4% in the timeliness of immunisation at 10 weeks and 9 months.
Conclusions
Findings suggest that segmenting populations into distinct clusters for differentiated program design and delivery may serve to improve reach and impact.

Strengths and limitations of this study:
Strengths

- The step-wise approach combining K-means and Lasso regression is well superior compared to other approaches involving only either supervised or unsupervised machine learning to handle data from household surveys.
- Findings suggest that segmenting populations into homogeneous groups can help to booster uptake of (D2B) mobile health communication programs.

Limitations
- The analysis included only those with a certain (higher than that of general population) level of access to mobile phones - survey respondents were required to have access to a mobile phone (own

a phone or have a phone they can use). While populations without a high level of access to phones may have different findings, our analysis presents what is typical of populations that are enrolled in direct to beneficiary programs.

- K-means algorithm has certain limitations, including problems associated with random initialization of the centroids which leads to unexpected convergence. Also, the empirical nature of the methods may limit the generalisability of the exact variables to other settings.

Introduction

Digital health solutions have the potential to address critical gaps in information access and service delivery, which underpin high mortality [1-9]. Mobile health communication programs, which provide information directly to beneficiaries, are among the few examples of digital health solutions to have scaled widely in a range of settings [10, 11]. Historically, these solutions have been designed as 'blunt instruments' – providing the same content, with the same frequency, using the same digital channel to large target populations. While this approach has enabled solutions to scale, it has contributed to variability in their reach and impact, due in part to differences in women's access to and use of mobile phones, particularly in low- and middle-income countries [12, 13].

Despite near ubiquitous ownership of mobile phones at a household level, a growing body of evidence suggests that there is a substantial gap between men and women's ownership, access to and use of mobile phones [14-16]. In India, there is a 45% gap between women's reported access to a phone and ownership at a household level [16]. Variations in the size of the gap have been observed across states and urban/rural areas, and by sociodemographic characteristics, including education, caste, and socioeconomic status [16]. Amongst women with reported access to a mobile phone, the gender gap further persists in the use of mobiles, in part because of patriarchal gender norms and limited digital skills [17]. Collectively, these gender gaps underscore the need to consider inequities in phone access and use patterns when designing and implementing D2B mobile health communication programs.

Kilkari, designed and scaled by BBC Media Action in collaboration with the Ministry of Health and Family Welfare, is India's largest direct to beneficiary mobile health information program. When BBC Media Action transitioned Kilkari to the national government in April 2019, it had been implemented in 13 states and reached over 10 million women and their families [3, 18, 19] . Evidence on the program's impact from a randomized control trial conducted in Madhya Pradesh, India, between 2018 and 2021, suggests that across study arms, Kilkari was associated with a 3.7% increase in modern reversible contraceptive use (RR: 1.12, 95% CI: 1.03 to 1.21, p=0.007), and a 2.0% decrease in the proportion of male or females sterilized since the birth of the child (RR: 0.85, 95% CI: 0.74 to 0.97, p=0.016) [3, 19]. The program's impact on contraceptive use, however, varied across key population sub-groups. Among women exposed to 50% or more of the Kilkari content as compared to those not exposed, differences in reversible method use were greatest for those in the poorest socioeconomic strata (15.8% higher), for those in disadvantaged castes (12.0% higher), and for those with any male child (9.9% higher) [3, 19]. Kilkari's overall and varied impact across beneficiary groups raises important questions about whether the differential targeting of women and their families might lead to efficiency gains and deepen impact.

In this manuscript, we argue that to maximize reach, exposure, and deepen impact, the future design of mobile health communication solutions will need to consider the heterogeneity of beneficiaries, including within husband-wife couples, and move away from a one-size-fits all model towards differentiated program design and delivery. Drawing from husbands' and wives' survey data captured as part of a randomised controlled trial of Kilkari in Madhya Pradesh India, we used a three-step process involving K-means clustering and Lasso (Least Absolute Shrinkage and Selection Operator) regression to segment couples into distinct clusters. We then assess differences in health behaviours across respondents in both study arms of the RCT. Findings are anticipated to inform future efforts to capture data and refine methods for segmenting

beneficiary populations and in turn optimizing the design and delivery of mobile health communication programs in India and elsewhere globally.

Methods

Kilkari program overview

Kilkari is an outbound service that makes weekly, stage-based, pre-recorded calls about reproductive, maternal, neonatal and child health (RMNCH) directly to families' mobile phones, starting from the second trimester of pregnancy until the child is one year old. Kilkari is comprised of 90 minutes of reproductive, maternal, newborn and child health content sent via 72 once weekly voice calls (average call duration: 1 minute, 15 seconds). Approximately 18% of cumulative call content is on family planning; 13% on child immunisation; 13% on nutrition; 12% on infant feeding; 10% on pregnancy care; 7% on entitlements; 7% on diarrhoea; 7% on postnatal care; and the remainder on a range of topics including intrapartum care, water and sanitation (WASH), and early childhood development. BBC Media Action designed and piloted Kilkari in the Indian state of Bihar in 2012-2013, and then redesigned and scaled it in collaboration with the Ministry of Health and Family Welfare between 2015 and 2019. Evidence on the evaluation design and program impact are reported elsewhere [20].

Setting

Data used in this analysis were collected from four districts of the central Indian state of Madhya Pradesh as part of the impact evaluation of Kilkari described elsewhere [3, 19]. Madhya Pradesh (population 75 million) is home to an estimated 20% of India's population and falls below national averages for most sociodemographic and health indicators [21]. Wide differences by gender and between urban and rural areas persist for wide range of indicators including literacy, phone access and health seeking behaviours. Among men and women 15-49 years of age, 59% of women (78% urban and 51% rural) were literate as compared to 82% of men in 2015-2016 [21]. Amongst literate women, 23% had 10 or more years of schooling (44% urban and 14% rural) [21]. Despite near universal access to phones at a household level, only 19% of women in rural areas and 50% in urban had access to a phone that they themselves could use in 2015 [21]. Among pregnant women, over half (52%) of pregnant women received the recommended four ANC visits in urban areas as compared to only 30% in rural areas [21]. Despite high rates of institutional delivery (94%) in urban areas, only 76% of women in rural areas reported delivering in a health facility in 2015 [21]. These disparities underscore the population heterogeneity within and across Madhya Pradesh.

Sample population

The sample for this study were obtained through cross-sectional surveys administered between 2018 and 2020 to women (n=5,095) with access to a mobile phone and their husbands (n=3,842) in four districts of Madhya Pradesh [20]. At the time of the first survey (2018-2019), the women were 4-7 months pregnant; the latter survey (2019-2020) re-interviewed the same women at 12 months postpartum. Their husbands were only interviewed once, during the latter survey round. The surveys spanned 1.5 hours in length. In this analysis, modules on household assets and member characteristics; phone access and use, including observed digital skills (navigate IVR prompts, give a missed call, store contacts on a phone, open SMS, read SMS) were used to develop models. Data on practice for maternal and child health behaviours, including infant and young child feeding, family planning, pregnancy and postpartum care were used to explore the differential impact of Kilkari across clusters but not used in the development of clusters [20].

Approach to segmentation

Figure 1 presents a framework used for developing homogenous clusters of men and women in four districts of rural Madhya Pradesh India. Box 1 describes the steps undertaken at each point in the framework in detail. We started with data elements collected on phone access and use as well as population sociodemographic characteristics collected as part of a cross-sectional survey described elsewhere[3, 22]. Unsupervised learning was undertaken using K-Means cluster and strong signals were identified. Strong signals were defined as variables that had at least a prevalence of 70% in one or more clusters and differed

For peer review only - http://bmjopen.bmj.com/site/about/guidelines.xhtml

from another cluster by 50% or more. For example, 6% of men own a smart phone in cluster 1, 88% in cluster 2 and 75% in cluster 3. Therefore, having a smart phone can be considered as a strong signal. Additional details are summarised in Box 1. Once defined, we then explored differences in health care practices across study clusters among those exposed and not exposed to Kilkari within each cluster.

Patient and public involvement

Patients were first engaged upon identification in their households as part of a household listing carried out in mid/ late 2018. Those meeting eligibility criteria were interviewed as part of the baseline survey, and ultimately randomized to the intervention and control arms. Prior to the administration of the baseline, a small number of patients were involved in the refinement of survey tools through qualitative interviews, including cognitive interviews, which were carried out to optimise survey questions, including the language and translation used. Finalised tools were administered to patients at baseline and endline, and for a sub-sample of the study population, additional interviews carried out over the phone and via qualitative interviews between the baseline and endline surveys. Unfortunately, because of COVID-19 patients and associated travel restrictions could not be involved in the dissemination of study findings.

For peer review only

Box 1. Step-wise process for developing and refining a machine learning approach for population segmentation

Data collected from special surveys like the couple's data set used here are relatively smaller in terms of sample size but large with regard to the number of data elements available. In such high dimensional data, there are many irrelevant dimensions which can mask existing clusters in noisy data, making more difficult the development of effective clustering methods [3, 23]. Several approaches have been proposed to address this problem. They can be grouped into two categories: static or adaptive *dimensionality reduction*, including principal components analysis (PCA) [24, 25] and *subspace clustering* consisting on selecting a small number of original dimensions (features) in some unsupervised way or using expert knowledge so that clusters become more obvious in the subspace [26, 27] . In this study we combined subspace clustering using expert knowledge and adaptive dimensionality reduction (Supplementary Figure 1) to find subspace where clusters are most well separated and well defined. Therefore, as part of subspace clustering, we chose to start with couples' survey data, including variables related to socio demographic characteristic, phone ownership, use and literacy (Supplementary Table 1). Emergent clusters were overlapping. We decided to use men's survey data on phone access and use as a starting point.

Step 1. Defining variables which characterise homogenous groups

Analyses started with a predefined set of data elements captured as part of a men's cross-sectional survey including sociodemographic characteristics and phone access and use. K-Means clustering was used to identify clusters and the elbow method was used to define the optimal number of clusters. Strong signals were then identified. Variables which had at least a prevalence of 70% in one or more clusters and differed from another cluster by 50% or more were considered to have a strong signal.

Step 2. Model strengthen through the identification and addition of new variables

Once an initial model was developed drawing from the predefined set of data from the men's survey and strong signals were identified, we reviewed available data from the combined dataset (data from the men's survey and women's survey). Signal strength was used as an outcome variable or target in a linear regression with L1 regularization or Lasso regression (Least Absolute Shrinkage and Selection Operator). Regularization is a technique used in supervised learning to avoid overfitting. Lasso Regression adds absolute value of magnitude of coefficient as penalty term to the loss function. The loss function becomes:

$$Loss = Error(y,y) + \alpha\sum_{i=1}^{N}|\omega_i|$$

where ω_i are coefficients of linear regression $y = \omega_1 x_1 + \omega_2 x_2 + \ldots + \omega_N x_N + b$

Lasso Regression works well for selecting features in very large datasets as it shrinks the less important features of coefficients to zero [28, 29]. Merged women's survey and men's survey data were used as predictors for the regression, excluding variables related to heath knowledge and practices. We ended up with a sample of 3,484 rows and 1,725 variables after data pre-processing.

Step 3. Refining clusters using supervised learning

We then re-ran K-Means clustering with three clusters (K=3) using important features selected by Lasso regression. This methodology was used to refine the clusters and subsequently identify new strong signals. After step 3 was conducted, we repeated step 2, and kept on iteratively repeating step 2 and 3 until there was no gain in strong signals. Data preparation and results formatting have been conducted in R 4.1.1 [30], K-means clustering has been performed in python 3.8.5 [31].

For peer review only

Figure 1. Framework for segmentation analysis

K-Means algorithm

As part of Steps 1 and 3, K-means algorithms were used (Box 1). We chose to use K-means algorithm because of its simplicity and speed to handle large dataset compared to hierarchical clustering [32]. A K-Means algorithm is one method of cluster analysis designed to uncover natural groupings within a heterogeneous population by minimizing Euclidean distance between them [33]. When using a K-Means algorithm, the first step is to choose the number of clusters K that will be generated. The algorithm starts by selecting K points randomly as the initial centres (also known as cluster means or centroids) and then iteratively assigns each observation to the nearest centre. Next, the algorithm computes the new mean value (centroid) of each cluster's new set of observation. K-Means re-iterates this process, assigning observations to the nearest centre. This process repeats until a new iteration no longer reassigns any observations to a new cluster (convergence). Four metrics have been used for the validation of clustering: within cluster sum of squares, silhouette index, Ray-Turi criterion and Calinski-Harabatz criterion. Elbow method was used to find the right K (number of clusters) [34]. Figure 2 is a chart showing the within cluster sum of squares (or inertia) by the number of groups (k value) chosen for several executions of the algorithm.

Figure 2. Elbow method used to help decide ultimate number of clusters appropriate for the data.

Inertia is a metric that shows how dissimilar the members of a group are. The less inertia there is, the more similarity there is within a cluster (compactness). The main purpose of clustering is not to find 100% compactness, it is rather to find a fair number of groups that could explain with satisfaction a considerable part of the data (k=3 in this case). Silhouette analysis helped to evaluate the goodness of clustering or clustering validation (*Figure 3*). It can be used to study the separation distance between the resulting clusters. The silhouette plot displays a measure of how close each point in one cluster is to points in the neighbouring clusters. This measure has a range of [-1, 1]. Silhouette coefficients near +1 indicate that the sample is far from the neighbouring clusters. A value of 0 indicates that the sample is very close to the decision boundary between two neighbouring clusters and negative values indicate that those samples might have been assigned to the wrong cluster. Figure 3 shows that choosing three clusters was more efficient than four for the data from the available surveys for two reasons: 1) there were less points with negative silhouettes, 2) the cluster size (thickness) was more uniform for three groupings. Other criterions used to evaluate quality of clustering are obtained by combining the 'within cluster compactness index' and 'between-cluster spacing index' [35]. Calinski-Harabatz criterion is given by: $C(k) = \frac{Trace(B)\ (n-k)}{Trace(W)\ (k-1)}$ and Ray-Turi criterion is given by $r(k) = \frac{distance\ (W)}{distance\ (B)}$ where B is the between-cluster covariance matrix (so high values of B denote well-separated clusters) and W is the within-cluster covariance matrix (so low values of W correspond to compact clusters) . They both ended up with same conclusions that 3 clusters were the best choice for the data we had. Supplementary Table 2 gives different metrics used and values obtained for various clusters.

Figure 3. Silhouette analysis for three and four clusters

Results

Sample characteristics

Supplementary Tables 3a and 3b summarise the sample characteristics by cluster for men and women interviewed. Figure 4 and Supplementary Table 4 presents select characteristics with 'strong signals' for each cluster.

For peer review only - http://bmjopen.bmj.com/site/about/guidelines.xhtml

Cluster 1 (n=1,408) constitutes 40% of the sample population and was comprised of men and women with low levels of digital access and skills (Figure 4). This cluster included the poorest segment of the sample population: 36% had a primary school or lower education and 40% were from a scheduled tribe/caste. Most men owned a feature (68%) or brick phone (22%); used the phone daily (89%); and while able to navigate IVR prompts (91%), only 29% were able to perform all of the five basic digital skills assessed. Women in this cluster similarly had lower levels of education as compared to other clusters (39% have primary school or less education); used feature (74%) or brick phones (8%); and had low digital skills (15% were able to perform the five basic digital skills assessed).

Cluster 2 (n=666; 19% of sample population), is comprised of men with mid-level and women with low digital access and skills. In this cluster, 75% of men owned smartphones, 65% were observed to successfully perform the five basic digital skills assessed, and 36% could perform a basic internet search. Men in Cluster 2 also self-reported accessing videos from YouTube (84%) and using WhatsApp (95%). Women in Cluster 2 had low phone ownership; nearly half of women reported owning a phone (38% owned a phone and did not share it, 22% owned and shared a phone) — findings which contradict their husbands' reports of 0% women's phone ownership. Only 21% of women in this cluster were observed to be able to successfully perform the five basic digital skills assessed. However, based on husband's reporting of their wives' digital skills, 36% of women could search the internet, 37% used WhatsApp, and 66% watched shows on someone else's phone.

Cluster 3 (n=1,410; 40% of sample population) is comprised of couples with high level digital access among both husbands and wives, and lower-level digital skill among wives (Figure 4). An estimated 67% of couples in this cluster were in the richer or richest socioeconomic strata, while 71% of men and 58% of women had high school or higher levels of education. Men in this cluster reported using the internet frequently (85%), were observed to own smart phones (88%), and had high levels of digital skills: 77% could perform the five basic digital skills assessed, 77% could perform a basic internet search, and 85% could send a WhatsApp message When reporting on their wife's digital access and skills, all men in this cluster reported that their wives' owned phones (100%), but often shared these phones with their husbands (77%), using them to watch shows (75%), search the internet (55%), or use WhatsApp (57%). However, a much lower level of women interviewed in this cluster were observed to own Feature (57%) or Smart phones (34%) and had moderate digital skills with 41% being able to successfully perform the five basic digital skills assessed.

Figure 4. Distribution of select characteristics with strong signals by Cluster

Differences in health outcomes by Cluster

Table 1 presents differences in health outcomes by Cluster among those exposed and not exposed to Kilkari as part of the randomised controlled trial in Madhya Pradesh. Findings suggest that the greatest impact was observed among those exposed to Kilkari in Cluster 2, which is the smallest cluster identified (19% of the sample population). Amongst this population, differences between exposed and not exposed were 8% for reversible modern contraceptive methods, 7% for immunisation at 10 weeks, 3% for immunisation at 9 months, and 4% for timely immunisation at 10 weeks and 9 months. Additionally, an 8% difference between exposed and not exposed was observed for the proportion of women who report being involved in the decision about what complementary foods to give child.

Among Clusters 1 and 3, improvements were observed among those exposed to Kilkari for a small number of outcomes. In Cluster 1, those exposed to Kilkari had a 3-4% higher rate of immunisation at 6, 10, 14 weeks than those not exposed. In both Clusters 1 and 3 the timeliness of immunisation improved at 10

For peer review only

weeks amongst those exposed. No improvements were observed for use of modern reversible contraception in either cluster.

For peer review only

Table 1. Differential impact of Kilkari exposure on family planning, infant feeding and immunizations per cluster

	Cluster1						Cluster2						Cluster3					
	Not exposed			Exposed			Not exposed			Exposed			Not exposed			Exposed		
	%	N	SE	%	N	SE	%	N	SE	%	N	SE	%	N	SE	%	N	SE
**Family planning**																		
Current modern family planning use	42	269	0.02	41	316	0.018	42	130	0.028	44	157	0.026	50	340	0.019	51	368	0.019
Reversible methods	29	183	0.018	30	232	0.017	30	94	0.026	38	133	0.026	41	280	0.019	44	319	0.018
Sterilized	12	77	0.013	10	80	0.011	11	33	0.017	8	30	0.015	10	66	0.011	7	54	0.01
Sterilized	18	114	0.015	16	121	0.013	15	47	0.02	12	44	0.018	14	99	0.013	12	84	0.012
**Infant and young child feeding**																		
Immediate breastfeeding	96	610	0.008	95	736	0.008	93	291	0.014	95	336	0.012	94	645	0.009	93	675	0.009
Gave child semi solid food yesterday	98	624	0.005	99	762	0.004	99	309	0.006	99	350	0.006	99	676	0.004	98	715	0.005
Exclusive breastfeeding	6	39	0.01	6	48	0.009	7	21	0.014	8	28	0.014	6	43	0.009	7	51	0.009
Fed child solid, semi-solid or soft foods the minimum number of times during the previous day	54	344	0.02	55	423	0.018	62	193	0.028	64	228	0.025	66	450	0.018	65	469	0.018
Minimum acceptable diet	27	171	0.018	28	219	0.016	29	91	0.026	26	92	0.023	25	170	0.017	27	198	0.017
Women involved in the decision about what complementary foods to give child	89	569	0.012	92	708	0.01	82	256	0.022	90	319	0.016	88	604	0.012	87	634	0.012
**Immunization**																		
Fully immunized	44	280	0.02	44	340	0.018	45	139	0.028	49	173	0.027	51	350	0.019	48	352	0.019
Birth	70	444	0.018	70	542	0.016	71	223	0.026	73	259	0.024	72	493	0.017	74	534	0.016
6 weeks	75	475	0.017	78	600	0.015	78	242	0.024	79	280	0.022	77	528	0.016	78	568	0.015
10 weeks	72	460	0.018	76	584	0.015	72	225	0.025	79	279	0.022	75	514	0.017	76	554	0.016
14 weeks	68	432	0.019	71	550	0.016	74	230	0.025	74	263	0.023	75	511	0.017	75	541	0.016
9 months	68	433	0.018	68	522	0.017	69	214	0.026	72	255	0.024	75	510	0.017	74	538	0.016
Timeliness: birth	69	438	0.018	67	515	0.017	68	213	0.026	69	246	0.025	70	477	0.018	72	525	0.017
Timeliness: 6 weeks	45	287	0.02	46	353	0.018	45	139	0.028	44	155	0.026	51	349	0.019	51	371	0.019
Timeliness: 10 weeks	25	162	0.017	28	217	0.016	23	71	0.024	27	94	0.024	31	213	0.018	34	248	0.018
Timeliness: 14 weeks	13	85	0.014	13	102	0.012	14	43	0.02	14	51	0.019	19	131	0.015	22	162	0.015
Timeliness: 9 months	14	89	0.014	13	99	0.012	12	37	0.018	16	55	0.019	18	126	0.015	17	126	0.014

Discussion

Evidence on the impact of direct to beneficiary mobile health communication programs is limited but broadly suggests that they can cost-effectively improve some reproductive, maternal and child health practices. This analysis aims to serve as a proof of concept for segmenting beneficiary populations to support the design of more targeted mobile health communication programs. We used a three-step iterative process involving a combination of supervised and unsupervised learning (K-means clustering and Lasso regression) to segment couples into distinct clusters. Three identifiable groups emerge each with differing health behaviours. Findings suggest that exposure the D2B program Kilkari may have a differential impact among the clusters.

Implications for designing future digital solutions

Findings demonstrate that the impact of the D2B solution Kilkari varied across homogenous clusters of women with access to mobile phones and their husbands in Madhya Pradesh. Across delivery channels, our analysis indicates that mobile health communication could not be effectively delivered to husbands and wives in Cluster 1 using WhatsApp, because smartphone ownership and WhatsApp use in this cluster are negligible. IVR, on the other hand, could be used to reach couples in Cluster 1, but reach is likely to be sporadic because of high levels of phone sharing with others (78% among men and 57% among women). On the other hand, WhatsApp and YouTube are likely to be effective digital channels for communicating with both husbands and wives in Cluster 3, where most men and women own or use smartphones and WhatsApp.

Beyond delivery channels, study findings raise a number of important learnings for content development as well as optimising beneficiary reach and exposure. The creative approach to content created for Cluster 3, where 40% of women are from the richest socio-economic status and only 17% have never been to school or have a Primary School education or less, would need to be very different from the creative approach to content created for Cluster 1, where 53% have a poorest or poorer socio-economic status, and 39% have never been to school or have a Primary School education or less. Similarly, this analysis adds to qualitative findings [17] and provides important insights into how gender norms related to women's use of mobile phones may effect reach and impact. While few (13-15%) husbands indicated that 'adults' need oversight to use mobile phones, men's perceptions varied when asked about specific use cases. Across all Clusters, nearly half of husbands indicated that their wives needed permission to pick up phone calls from unknown numbers – an important insight for IVR programs which may make outbound calls without pre-warning to beneficiaries. In Clusters 1 and 2, 25% and 29% of husband's, respectively, report that their wives need permission to answer calls from health workers – as compared to 15% in Cluster 3. While restrictions on SMS and WhatsApp were lower than making or receiving calls, these channels are less viable given women's limited access to smartphones, low literacy and digital skills. Overall, men's perceptions on the restrictions needed on the receipt and placement of calls by women was lower for Cluster 3. However, despite the relative wealth of beneficiaries in Cluster 3 (67% were in the richer or richest socioeconomic strata), 48% of women had zero balance on their mobile phones at the time of interview. Collectively, these findings highlight the immense challenges which underpin efforts to facilitate women's phone access and use. They too underline the criticality of designing mobile health communication content for couples, rather than just wives to ensure the buy-in of male gatekeepers, and for continuing to prioritize face to face communication with women on critical health issues.

Approach to segmentation

Data in our sample were captured as part of special surveys carried out through the impact evaluation of Kilkari. Future programs may be tempted to apply the approach undertaken here to existing datasets, including routine health information systems or other forms of government tracking data. In the India context, while these data are likely to be less costly than special surveys, they are comparatively limited in terms of data elements captured – particularly in terms of data ownership of different types of mobile devices, digital skill levels and usage of specific applications or social media platforms. Data quality may

also be a significant issue in existing datasets . For example, we estimate that SIM change in our study population was 44% over a 12-month period – a factor which when coupled with the absence of systems to update government tracking registries raises important questions about who is retained in these databases, and therefore able to receive mobile health communications—and who is missing. Amongst the variables used, men's phone access and use were most integral to developing distinct clusters. We recommend that future surveys seeking to generate data for designing digital services for women ensure that data elements are captured on men's phone access and use practices as well as their perception of their wife's phone access and use.

In addition to underlying data, our analytic approach differed from other segmentation analyses. . Our work is relatively new in global health literature related to digital health programs that are positioned as D2B programs. While similar ML models are being tested in various domains related to public health, they consist exclusively of unsupervised learning [36, 37] or supervised learning [1, 6, 38, 39], this analysis is the first of its kind focusing on the use of a combination of supervised and unsupervised learning to identify homogenous clusters for targeting of digital health programs. Data collected from special surveys like the couple's data set used here are comparatively smaller in terms of sample size but large with regard to the number of data elements available. An alternative approach to that described in this manuscript might be to develop strata based on population characteristics. Indeed, findings from the impact evaluation published elsewhere suggest that women with access to phones in the most disadvantaged sociodemographic strata (poorest (15.8% higher) and disadvantaged castes (12% higher)) had greater impact when exposed to 50% or more of the Kilkari content as compared to those not exposed. With an approach to segmentation based on these strata of highest impact, we know and understand what divides or groups respondents (e.g. socioeconomic status, education) but this may not be enough when they do not explain the underlying reasons for change. In the approach used here, the study population is segmented using multiple characteristics (sociodemographic, digital access and use) simultaneously. The results are clusters comprised of individuals with mixed sociodemographic characteristics which may help to explain the reduced impact observed on health outcomes. Designing a strategy based on previously known / identifiable strata alone has been the basis of targeting in public health but has not maximized reach, exposure and effect to its fullest potential. The approach used here may better group beneficiaries based on their digital access and use characteristics which may serve to increase reach and exposure. However, further research is needed to determine how to deepen impact within these digital clusters.

Conclusions

Study findings sought to identify distinct clusters of husbands and wives based on their sociodemographic, phone access and use characteristics, and to explore the differential impact of a maternal mobile messaging program across these clusters. Three identifiable groups emerge each with differing levels of digital access and use. Descriptive analyses suggest that improvements in some health behaviours were observed for a greater number of outcomes in Cluster 2, than in Clusters 1 and 3. These findings suggest that one size fits all mobile health communications solutions may only engage one segment of a target beneficiary population, and offer much promise for future direct to beneficiary and other digital health programs which could see greater reach, exposure and impact through differentiated design and implementation. More quantitative and qualitative work is needed to better understand factors driving the differences in impact and what is likely to motivate adoption of target behaviours in different clusters. Our work opens up a new avenue of research into better targeting of beneficiaries using data on variety of domains including socio-demographics, mobile phone access and use. Future work will entail evaluation of the actual platform used for targeting and delivery of the program in pilot projects. Successful pilots can be scaled up to larger swathes of the population in India and similar setting around the world.

For peer review only

Acknowledgments: We thank the women and families of Madhya Pradesh who generously gave of their time to support this work. We are humbled by the opportunity to convey their perspectives and experiences. We additionally are grateful to Dr. Rajani Ved at the National Health Systems Resource Centre for her support. This work was made possible by the Bill and Melinda Gates Foundation. We thank Diva Dhar, Suhel Bidani, Rahul Mullick, Dr. Suneeta Krishnan, Dr. Neeta Goel and Dr. Priya Nanda for believing in us and giving us this opportunity. We additionally wish to thank BBC Media Action teams in India and London for their partnership and collaboration. The evaluation was unquestionably strengthened by their support, transparency, and willingness to work with us on all facets of the research. We too are grateful to the larger team of enumerators from OPM-India who worked tirelessly over many months to implement the surveys that form the backbone of our analyses. We additionally thank Prabal Singh, Vinit Pattnaik at OPM and Alain Labrique, Smisha Agarwal, and Erica Crawford at Johns Hopkins University for their support. Lastly, our figures have been beautified by the great and ever patient Dan Harder of the Creativity Club UK. We thank him for his work.

Contributions: JJHB conducted the analysis and wrote the paper with AEL and inputs from DM, SC, and other authors. AEL is the overall study PI, helped to secure the funding, led the design of the study tools, supported oversight of field work and analysis, and wrote the manuscript with JJHB and DM. DM helped to secure funding, helmed the study design including sampling and randomisation, helped draft study tools, provided input to data analysis, and edited the manuscript. SC helped to secure the funding, draft and review study tools, interpret data analyses and study findings, and edit the manuscript. AG, KS, helped to draft and review study tools, interpret data analyses and study findings, and edit the manuscript. OU help to revise study tools, interpret data analyses, and edited the manuscript. NM is the UCT study PI and provided input to study design, oversight to the analysis and interpretation, and edited the manuscript.

Competing interests: All authors have completed the Unified Competing Interest form (available on request from the corresponding author) and declare that the research reported was funded by the Bill and Melinda Gates Foundation. AG and SC are employed by BBC Media Action; one of the entities supporting program implementation. The authors do not have other relationships and are not engaged in activities that could appear to have influenced the submitted work.

Funding: Bill and Melinda Gates Foundation grant number OPP1179252

Data sharing: The anonymised raw data are available upon request.

Ethics: Institutional Review Boards from the Johns Hopkins Bloomberg School of Public Health in Baltimore, Maryland USA and Sigma Research and Consulting in Delhi, India provided ethical clearance for study activities. Verbal informed consent was obtained from all study participants.

References

[1] A.K. Dey, N. Dehingia, N. Bhan, E.E. Thomas, L. McDougal, S. Averbach, J. McAuley, A. Singh, A.J.S.-P.H. Raj, Using machine learning to understand determinants of IUD use in India: Analyses of the National Family Health Surveys (NFHS-4), 19 (2022) 101234.
[2] N.U.Z. Khan, S. Rasheed, T. Sharmin, A. Siddique, M. Dibley, A.J.B.h.s.r. Alam, How can mobile phones be used to improve nutrition service delivery in rural Bangladesh?, 18(1) (2018) 1-10.

[3] A.E. LeFevre, N. Shah, K. Scott, S. Chamberlain, O. Ummer, J.J.H. Bashingwa, A. Chakraborty, A. Godfrey, P. Dutt, R.J.B.g.h. Ved, The impact of a direct to beneficiary mobile communication program on reproductive and child health outcomes: a randomised controlled trial in India, 6(Suppl 5) (2022) e008838.
[4] D. Mohan, J.J.H. Bashingwa, K. Scott, S. Arora, S. Rahul, N. Mulder, S. Chamberlain, A.E.J.B.g.h. LeFevre, Optimising the reach of mobile health messaging programmes: an analysis of system generated data for the Kilkari programme across 13 states in India, 6(Suppl 5) (2022) e009395.
[5] M. Njoroge, D. Zurovac, E.A. Ogara, J. Chuma, D.J.B.r.n. Kirigia, Assessing the feasibility of eHealth and mHealth: a systematic review and analysis of initiatives implemented in Kenya, 10(1) (2017) 1-11.
[6] A. Raj, N. Dehingia, A. Singh, L. McDougal, J.J.S.-p.h. McAuley, Application of machine learning to understand child marriage in India, 12 (2020) 100687.
[7] S. Siddique, J.C.J.E. Chow, Machine learning in healthcare communication, 1(1) (2021) 220-239.
[8] M. Deshmukh, P.J.W. Mechael, DC: mHealth Alliance, Addressing gender and women's empowerment in mHealth for MNCH: An analytical framework, (2013).
[9] S. Lund, M. Hemed, B.B. Nielsen, A. Said, K. Said, M. Makungu, V.J.B.A.I.J.o.O. Rasch, Gynaecology, Mobile phones as a health communication tool to improve skilled attendance at delivery in Zanzibar: a cluster-randomised controlled trial, 119(10) (2012) 1256-1264.
[10] J.J.H. Bashingwa, D. Mohan, S. Chamberlain, S. Arora, J. Mendiratta, S. Rahul, V. Chauhan, K. Scott, N. Shah, O.J.B.G.H. Ummer, Assessing exposure to Kilkari: a big data analysis of a large maternal mobile messaging service across 13 states in India, 6(Suppl 5) (2021) e005213.
[11] J.J.H. Bashingwa, N. Shah, D. Mohan, K. Scott, S. Chamberlain, N. Mulder, S. Rahul, S. Arora, A. Chakraborty, O.J.B.g.h. Ummer, Examining the reach and exposure of a mobile phone-based training programme for frontline health workers (ASHAs) in 13 states across India, 6(Suppl 5) (2021) e005299.
[12] A. LeFevre, S. Chamberlain, N. Singh, K. Scott, P. Menon, P. Barron, R. Ved, A. George, Avoiding the Road to Nowhere: Policy Insights on Scaling up and Sustaining Digital Health, Global Policy (2021).
[13] A. Swartz, A.E. LeFevre, S. Perera, M.V. Kinney, A.S. George, Multiple pathways to scaling up and sustainability: an exploration of digital health solutions in South Africa, Global Health 17(1) (2021) 77.
[14] GSMA, Connected women: The mobile gender gap report 2020, GSM Association (2020).
[15] A.E. LeFevre, N. Shah, J.J.H. Bashingwa, A.S. George, D. Mohan, Does women's mobile phone ownership matter for health? Evidence from 15 countries, BMJ Glob Health 5(5) (2020).
[16] D. Mohan, J.J.H. Bashingwa, N. Tiffin, D. Dhar, N. Mulder, A. George, A.E. LeFevre, Does having a mobile phone matter? Linking phone access among women to health in India: An exploratory analysis of the National Family Health Survey, PLoS One 15(7) (2020) e0236078.
[17] K. Scott, O. Ummer, A. Shinde, M. Sharma, S. Yadav, A. Jairath, N. Purty, N. Shah, D. Mohan, S.J.B.G.H. Chamberlain, Another voice in the crowd: the challenge of changing family planning and child feeding practices through mHealth messaging in rural central India, 6(Suppl 5) (2021) e005868.

For peer review only

[18] D. Mohan, J.J.H. Bashingwa, P. Dane, S. Chamberlain, N. Tiffin, A.J.J.r.p. Lefevre, Use of big data and machine learning methods in the monitoring and evaluation of digital health programs in India: An exploratory protocol, 8(5) (2019) e11456.
[19] A. LeFevre, N. Shah, K. Scott, S. Chamberlain, O. Ummer, J.J.H. Bashingwa, A. Chakraborty, A. Godfrey, P. Dutt, D. Mohan, Are stage-based, direct to beneficiary mobile communication programs effective in improving maternal newborn and child health outcomes in India? Results from an individually randomised controlled trial of a national programme, BMJ Global Health, In press (2021).
[20] A. LeFevre, S. Agarwal, S. Chamberlain, K. Scott, A. Godfrey, R. Chandra, A. Singh, N. Shah, D. Dhar, A. Labrique, A. Bhatnagar, D. Mohan, Are stage-based health information messages effective and good value for money in improving maternal newborn and child health outcomes in India? Protocol for an individually randomized controlled trial, Trials 20(1) (2019) 272.
[21] I.I.f.P. Sciences, National Family Health Survey 2015-2016 State Fact Sheet Madhya Pradesh. Mumbai: International Institute for Population Sciences, Government of India, Ministry of Health and Family Welfare; 2016.
[22] A. LeFevre, N. Shah, K. Scott, S. Chamberlain, O. Ummer, J.J. Bashingwa, A. Chakraborty, R. Ved, D. Mohan, Are stage-based mobile health information messages effective in improving maternal newborn and child health outcomes in India? Results from an individually randomized controlled trial Submitted Lancet GH (2021).
[23] B. Dash, D. Mishra, A. Rath, M.J.I.J.o.E. Acharya, Science, Technology, A hybridized K-means clustering approach for high dimensional dataset, 2(2) (2010) 59-66.
[24] C. Ding, X. He, H. Zha, H.D. Simon, Adaptive dimension reduction for clustering high dimensional data, 2002 IEEE International Conference on Data Mining, 2002. Proceedings., IEEE, 2002, pp. 147-154.
[25] S.J.a.p.a. Dasgupta, Experiments with random projection, (2013).
[26] L. Parsons, E. Haque, H.J.A.s.e.n. Liu, Subspace clustering for high dimensional data: a review, 6(1) (2004) 90-105.
[27] C. Ding, T. Li, Adaptive dimension reduction using discriminant analysis and k-means clustering, Proceedings of the 24th international conference on Machine learning, 2007, pp. 521-528.
[28] R. Muthukrishnan, R. Rohini, LASSO: a feature selection technique in predictive modeling for machine learning, 2016 IEEE international conference on advances in computer applications (ICACA), IEEE, 2016, pp. 18-20.
[29] M. Yamada, W. Jitkrittum, L. Sigal, E.P. Xing, M.J.N.c. Sugiyama, High-dimensional feature selection by feature-wise kernelized lasso, 26(1) (2014) 185-207.
[30] J.M. Chambers, Software for data analysis: programming with R, Springer2008.
[31] F. Milano, A Python-based software tool for power system analysis, 2013 IEEE Power & Energy Society General Meeting, IEEE, 2013, pp. 1-5.
[32] N. Dhanachandra, K. Manglem, Y.J.J.P.C.S. Chanu, Image segmentation using K-means clustering algorithm and subtractive clustering algorithm, 54 (2015) 764-771.
[33] A. Likas, N. Vlassis, J.J.J.P.r. Verbeek, The global k-means clustering algorithm, 36(2) (2003) 451-461.
[34] T.M. Kodinariya, P.R.J.I.J. Makwana, Review on determining number of Cluster in K-Means Clustering, 1(6) (2013) 90-95.

For peer review only

[35] C. Genolini, X. Alacoque, M. Sentenac, C.J.J.o.S.S. Arnaud, kml and kml3d: R packages to cluster longitudinal data, 65(4) (2015) 1-34.
[36] M. Liao, Y. Li, F. Kianifard, E. Obi, S.J.B.n. Arcona, Cluster analysis and its application to healthcare claims data: a study of end-stage renal disease patients who initiated hemodialysis, 17(1) (2016) 1-14.
[37] C. Violán, A. Roso-Llorach, Q. Foguet-Boreu, M. Guisado-Clavero, M. Pons-Vigués, E. Pujol-Ribera, J.M.J.B.f.p. Valderas, Multimorbidity patterns with K-means nonhierarchical cluster analysis, 19(1) (2018) 1-11.
[38] R. Das, S. Saleh, I. Nielsen, A. Kaviraj, P. Sharma, K. Dey, S.J.I.J.o.M.I. Saha, Performance analysis of machine learning algorithms and screening formulae for β–thalassemia trait screening of Indian antenatal women, 167 (2022) 104866.
[39] T.M. Santos, B.O. Cata-Preta, C.G. Victora, A.J.J.V. Barros, Finding Children with High Risk of Non-Vaccination in 92 Low-and Middle-Income Countries: A Decision Tree Approach, 9(6) (2021) 646.

Figure 1. Framework for segmentation analysis
Figure 2. Elbow method used to help decide ultimate number of clusters appropriate for the data.
Figure 3. Silhouette analysis for three and four clusters
Figure 4. Distribution of select characteristics with strong signals by Cluster.
Variables which had at least a prevalence of 70% in one or more clusters and differed from another cluster by 50% or more were considered to have a strong signal (*Reported by men interviewed, **Observed by survey enumerators)

For peer review only

Figure 1. Framework for segmentation analysis.

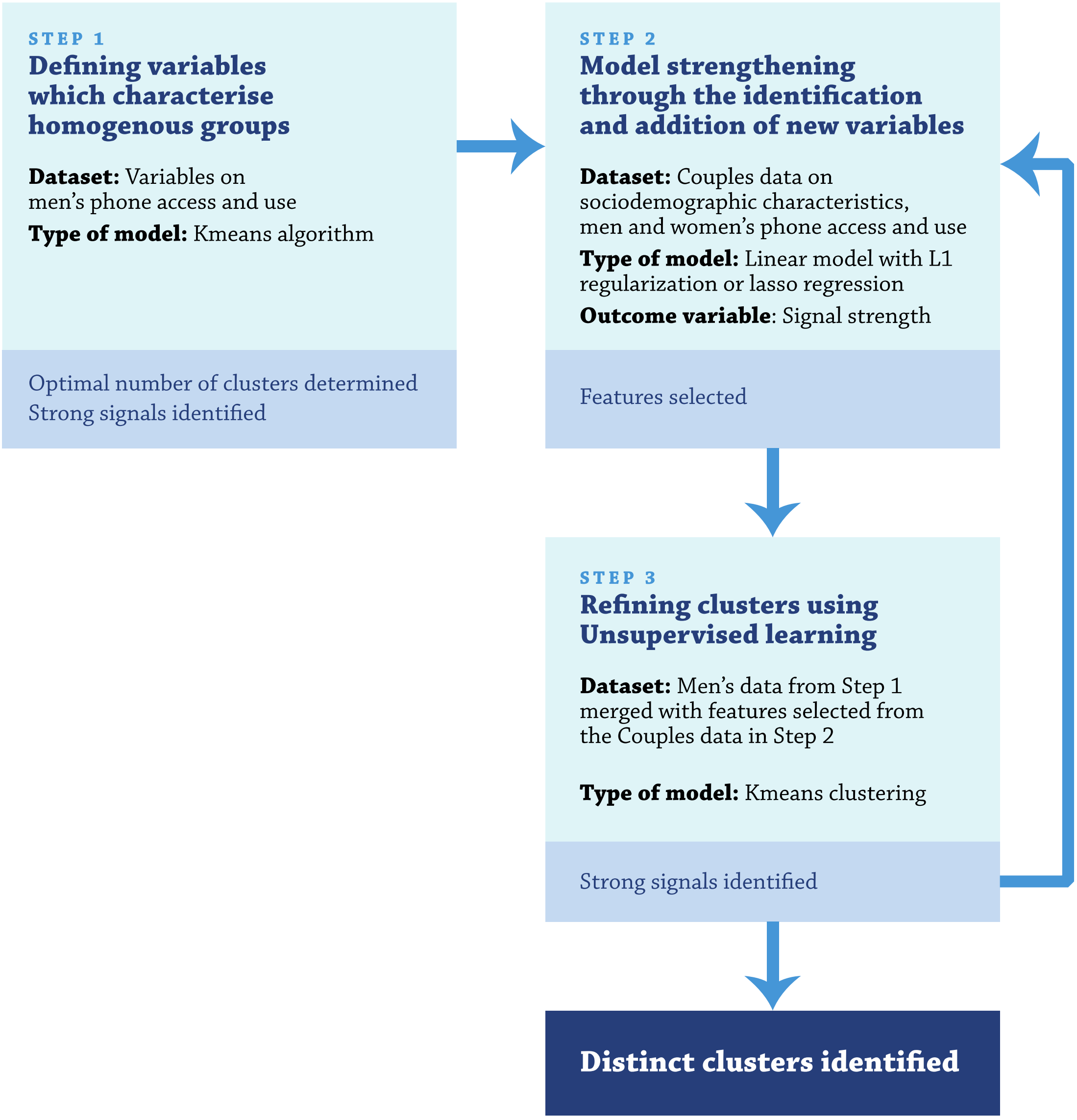

Figure 2. Elbow method used to help decide ultimate number of clusters appropriate for the data.

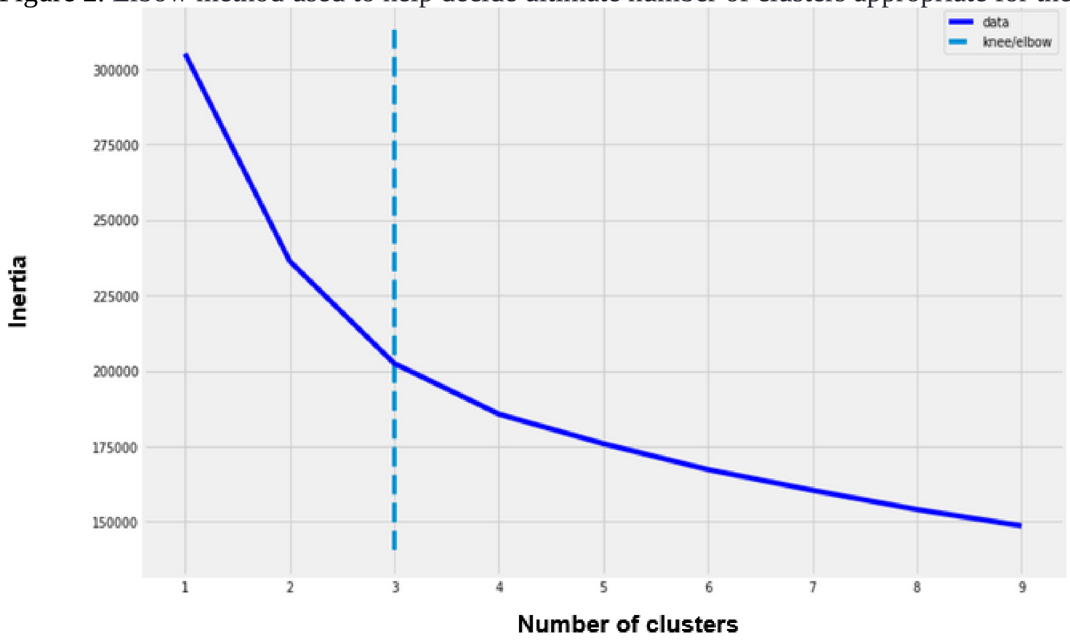

For peer review only

Figure 3. Silhouette analysis for three and four clusters

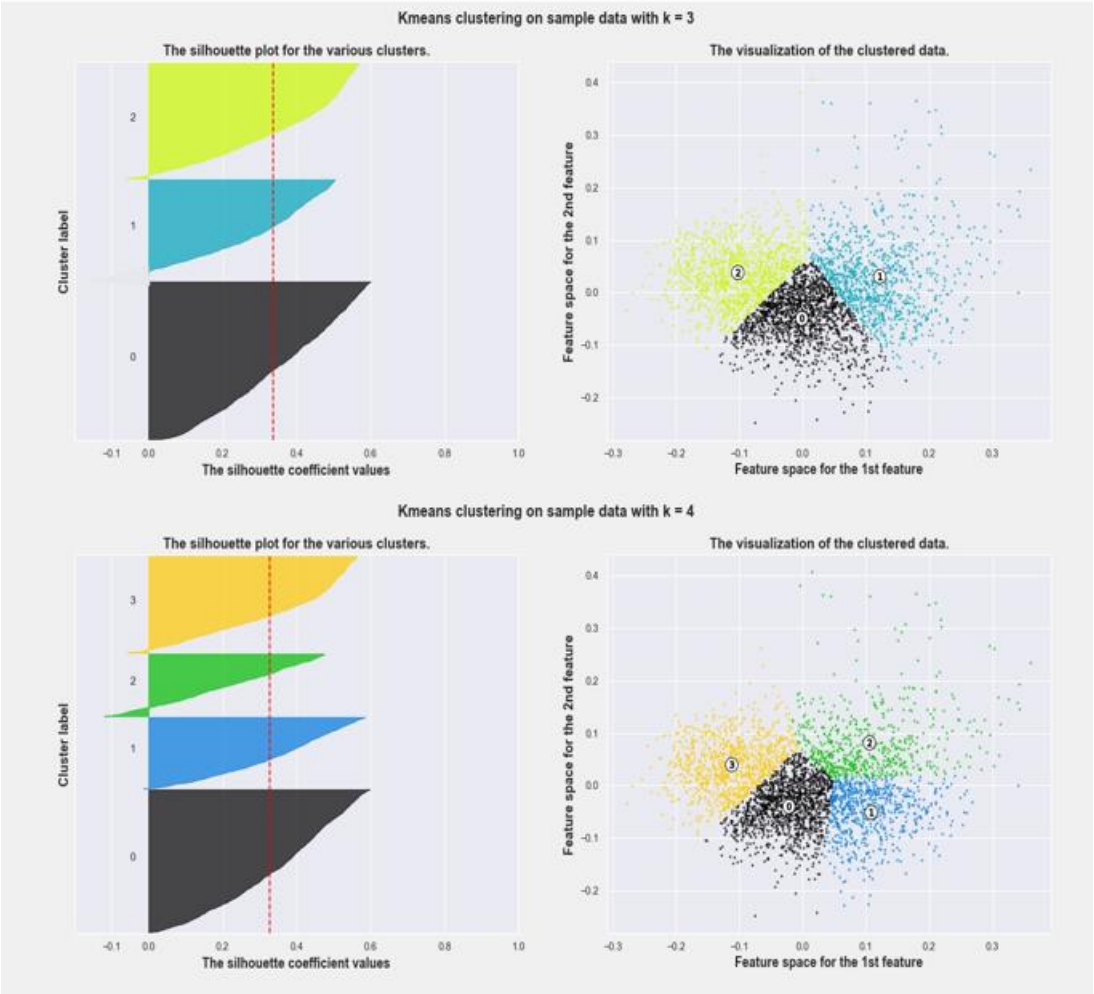

Figure 4. Distribution of select characteristics with strong signals by Cluster. Variables which had at least a prevalence of 70% in one or more clusters and differed from another cluster by 50% or more were considered to have a strong signal.

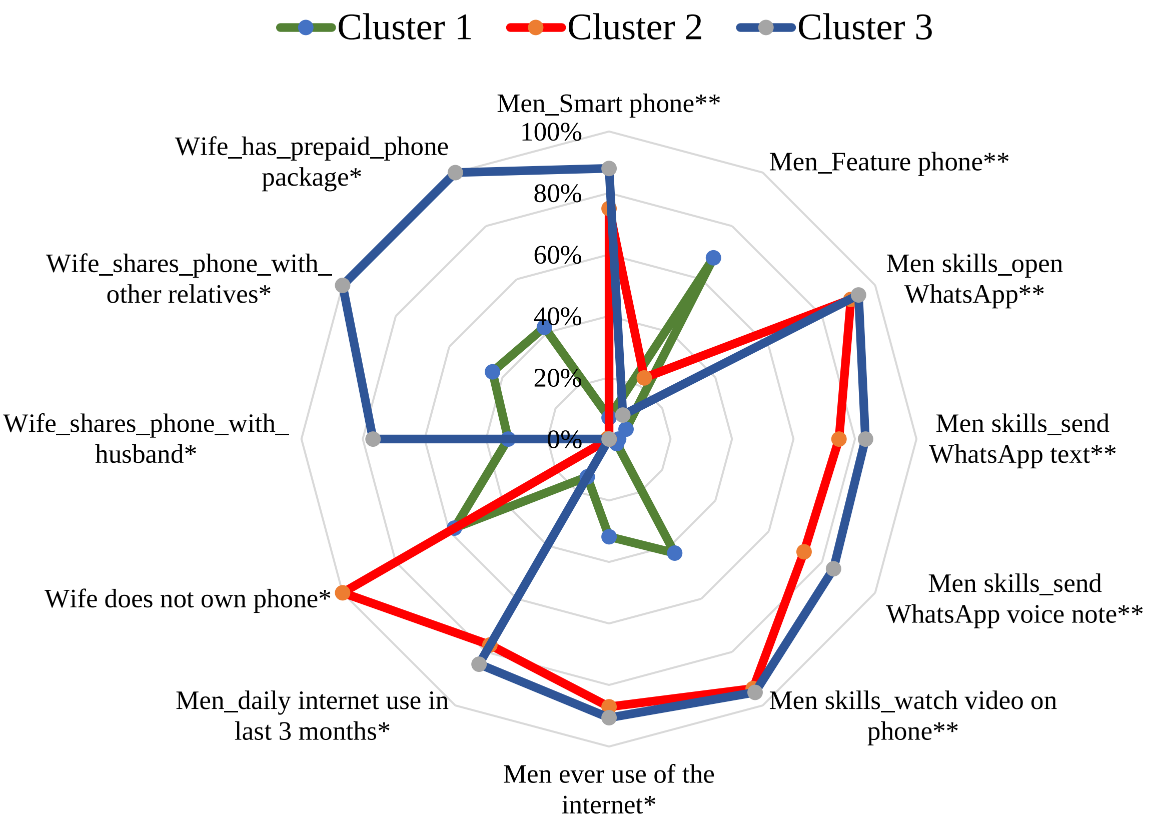

*Reported by men interviewed
**Observed by survey enumerators

For peer review only - http://bmjopen.bmj.com/site/about/guidelines.xhtml

Supplementary Table1. Study sample characteristics (variables used as starting point for couple's survey data)

	Women's survey		Men's survey	
**Variables**	N	%	N	%
**Education**				
0-5 years	610	18	586	17
>5 years	2874	82	2898	83
**District**				
Hoshangabad	345	10	345	10
Mandsaur	676	19	676	19
Rajgarh	791	23	791	23
Rewa	1672	48	1672	48
**Ethnicity/Caste**				
General	780	22	698	20
OBC	1690	49	1738	50
Scheduled caste	647	19	690	20
Scheduled tribe	345	10	357	10
**Age at time of enrollment in years**				
18-24	2027	58	564	16
25-34	1391	40	2477	71
35+	66	2	443	13
**Education**				
Never been to school	347	10	100	3
Primary school or less	610	18	586	17
Middle school	1042	30	932	27
High school	1168	34	1322	38
Higher education	317	9	544	16
**MNO**				
Airtel	893	26	791	23
Idea	1572	45	967	28
Jio	229	7	1270	36
Tata	9	0	4	0
vodafone	781	22	427	12
BSNL			24	1
**Frequency of most recent top up**				
More than 3 months	299	9		
Within 1 month	1626	47		

Within 1 week	718	21		
Within 3 months	841	24		
**Who topped up credit**				
Husband	2784	80		
Other	357	10		
self	343	10		
**Who taught respondent how to use phone**				
Husband	794	23		
Other	178	5		
Self	2512	72		
**Permission for wife's phone use**				
Wife takes permission to make call	1133	33		
Wife takes permission before picking up call	1614	46		
Wife takes permission to recharge	838	24		
Women need oversight to use phone	2514	72		
**Type of phone**				
Brick phone	454	13	357	10
Feature phone	2206	63	1234	35
Smart phone	824	24	1838	53
**Use phone to call spouse**	2563	74	2926	84
**Use phone to call ASHAs**	293	8	2478	71
**Use phone for internet**	1	0	1417	41
**Use phone to listen radio**	1	0	1868	54
**Observe phone**				
Phone working	2820	81	3251	93
**Digital Tasks**				
Able to navigate IVR prompts	2995	86	3319	95
Give a missed call	2409	69	2890	83
Store contacts on phone	2845	82	2999	86
Open SMS	1654	47	2966	85
Read SMS	1102	32	2188	63
Overall digital literacy	937	27	1938	56
Open and read SMS	1102	32	2188	63
**Involvement in Decision making**				
About daily household expenditures	713	20	2065	59
About big expenditures	623	18	2243	64

About health during pregnancy	937	27	3081	88
**Employment status**	1398	40	3458	99
**Socio-economic status**				
Poorest	542	16	542	16
Poorer	646	19	646	19
Middle	710	20	710	20
Richer	760	22	760	22
Richest	826	24	826	24
**Phone in the household**				
1	759	22	759	22
2	1437	41	1437	41
>2	1288	37	1288	37
**Parity**				
No child	1406	40	1406	40
One child	1256	36	1256	36
Two and more	822	24	822	24
**Religion**				
Hindu	3297	95	3297	95
Muslim	183	5	183	5
Other	4	0	4	0
**Frequency of phone use in last 3 months**				
Every day	2700	77		
not every day	784	23		
**Age at marriage**				
0-15 years	416	12		
>15 years	3068	88		

For peer review only - http://bmjopen.bmj.com/site/about/guidelines.xhtml

Supplementary Table 2. Metrics used for cluster validation (Davies-Bouldin and Calinski-Harabatz criterions have been normalized to [0,1] ,1 indicating a good partition)

Number of clusters	**Within cluster sum of square**	**Silhouette index**	**Ray -Turi index**	**Calinski – Harabatz index**
**2**	64791,07	0,812424	0,873942	0,820123
**3**	62595,37	0,801119	1	0,9563
**4**	60983,52	0,509252	0,853942	0,360082
**5**	59662,45	0,466859	0,529231	0,243941
**6**	58571,27	0,454165	0,482203	0,161834
**7**	57686,73	0,420884	0,427094	0,096974
**8**	56943,46	0,402445	0,249373	0,044445
**9**	56322,05	0,386873	0,268434	0

Table 3a. Men's sample characteristics by cluster based on Men's survey data from four districts of Madhya Pradesh

	**Total** n=3,484		**Cluster 1** n=1,408		**Cluster 2** n=666		**Cluster 3** n=1,410	
	%	n	%	n	%	n	%	n
**Sociodemographic characteristics**								
**Caste**								
General	20	698	15	208	17	112	27	378
OBC	50	1 738	45	637	50	334	54	767
Scheduled tribe	10	357	15	213	11	73	5	71
Scheduled caste	20	690	25	350	22	146	14	194
**Education**								
Never been to school	3	100	7	92	1	6	-	2
Primary school or less	17	586	29	403	13	84	7	99
Middle school	27	932	32	446	28	189	21	297
High school	38	1 322	29	415	42	280	44	627
Higher education	16	544	4	52	16	107	27	385
**Number of phones in the household**								
0-1	22	759	34	476	24	157	9	126
2	41	1 437	45	629	43	284	37	524
3+	37	1 288	22	303	34	225	54	760

**Phone ownership and sharing**								
Own phone and do not share	17	578	16	221	8	50	22	307
Own phone and do share	78	2 730	73	1 031	91	607	77	1 092
Share only	3	93	5	73	1	9	1	11
**Phone type (observed)**								
Brick phone	10	357	22	304	3	17	3	36
Feature phone	35	1 234	68	953	23	151	9	130
Smart phone	53	1 838	7	96	75	498	88	1 244
**Men's phone use**								
Daily phone use (reported)	95	3 327	89	1 260	99	662	100	1 405
**Phone features used (reported)**								
Calls	98	3 422	96	1 350	100	666	100	1 406
SMS	46	1 615	19	263	55	369	70	983
WhatsApp	61	2 109	7	97	95	635	98	1 377
Watch video	80	2 784	52	726	99	659	99	1 399
Share video	58	2 008	6	87	89	591	94	1 330
Make video	35	1 209	9	121	47	316	55	772
Download Apps	47	1 640	2	29	70	468	81	1 143
Music	86	2 984	68	959	97	649	98	1 376
Radio	26	889	14	200	32	210	34	479
Search Google	55	1 925	9	128	82	548	89	1 249
Search YouTube	67	2 327	21	300	98	653	97	1 374
Camera	84	2 921	61	857	99	659	100	1 405
Share photo	59	2 039	7	93	90	602	95	1 344
Mobile money	16	560	0	3	15	103	32	454
Transfer mobile money	13	463	0	1	12	82	27	380
Transfer mobile credit	13	459	0	1	12	83	27	375
**Men's Digital skills (observed)**								
Able to navigate IVR prompts	95	3 319	91	1 280	98	656	98	1 383
Give a missed call	83	2 890	72	1 020	88	588	91	1 282
Store contacts on phone	86	2 999	73	1 031	94	623	95	1 345
Open SMS	85	2 966	71	994	94	624	96	1 348
Read SMS	63	2 188	38	530	73	483	83	1 175
Overall Basic Digital Skill Level	56	1 938	29	415	65	432	77	1 091
**WhatsApp skills (observed)**								
Open WhatsApp	58	2 017	6	91	91	605	94	1 321
Send WhatsApp text	49	1 718	3	44	75	498	83	1 176
Send WhatsApp voice note	49	1 719	3	42	73	488	84	1 189
**Watch video on phone (observed)**	74	2 568	43	603	94	624	95	1 341
**Men report getting images and videos from**								

Internet: YouTube	59	2 062	19	274	83	554	88	1 234
Internet: Google	45	1 569	9	130	64	429	72	1 010
Other relatives	36	1 249	4	63	54	360	59	826
Friends locally	55	1 916	11	153	83	550	86	1 213
Friends other states	25	885	1	21	36	238	44	626
**Computer/ tablet ownership and use**								
Own Computer/ tablet	6	220	1	13	4	28	13	179
Daily computer / tablet use	5	184	0	3	5	30	11	151
Ever use of the internet from any device/ location (reported)	66	2 305	32	447	87	580	91	1 278
Daily internet use in last 3 months (reported)	55	1 906	14	199	77	515	85	1 192
**Wife owns phone**	57	3 484	42	591	-	-	100	1 410
**Wife's phone type**								
Brick phone	10	363	10	134	0	1	16	228
Feature phone	29	1 016	27	375	-	-	45	641
Smart phone	19	647	8	106	-	-	38	541
**Wife shares phone with**								
Husband	44	1 543	33	461	-	-	77	1 082
Children (male or female)	5	180	4	52	-	-	9	128
Parents in law	9	329	6	83	-	-	17	246
Wife's parents	3	107	2	33	-	-	5	74
Other relatives	58	2 028	44	615	0	3	100	1 410
Friend/ neighbour	1	30	1	9	-	-	1	21
**Phone features wife uses (reported)**								
Calls: receive, dial, or speak	100	3 475	100	1 404	100	663	100	1 408
SMS	33	1 146	16	228	28	185	52	733
WhatsApp	35	1 225	11	155	38	255	58	815
Watch shows	54	1 871	26	368	68	450	75	1 053
Music or radio	100	3 484	100	1 408	100	666	100	1 410
Search internet	34	1 192	12	168	36	240	56	784
Camera	74	2 589	55	772	84	559	89	1 258
**Men's perceptions about restrictions (if any) which should be placed on phone use**								
**No restrictions should be placed on adult phone use**	86	2 992	85	1 192	86	571	87	1 229
**Oversight needed for**								
Men	47	1 647	54	767	46	307	41	573
Women	72	2 514	79	1 114	71	476	66	924
Male children	82	2 863	86	1 207	79	523	80	1 133
Female children	92	3 198	93	1 311	91	608	91	1 279
**Men report that their wife needs their permission to pick up**								

**calls from**								
Someone unknown	46	1 614	46	653	51	341	44	620
Family	13	461	17	237	18	122	7	102
Friends/ Neighbours	32	1 121	35	488	41	274	25	359
Health workers	22	757	25	356	29	195	15	206
Business associates	28	990	29	410	35	232	25	348
**Men report women need their permission to make a call to**								
Family	17	600	21	293	24	162	10	145
Friends/ Neighbours	21	735	25	345	28	187	14	203
Health workers	20	692	22	315	29	192	13	185
Business associates	14	484	17	236	16	109	10	139
Unknown to husband	17	608	20	286	20	134	13	188
**Men report women need their permission to send SMS or WhatsApp to**								
Family	2	72	1	12	4	28	2	32
Friends/ Neighbours	3	101	1	12	6	41	3	48
Health workers	2	77	1	9	5	30	3	38
Business associates	2	54	1	11	3	18	2	25
Unknown to husband	3	100	1	13	5	35	4	52
**Man has concerns about wife's phone ownership or use**	1	24	1	10	2	11	0	3
**Reasons for concern (multi-select):**								
Cost of phone	0	3	0	1	0	2	-	-
Cost of using phone	0	9	0	4	0	2	0	3
Reputational risk	0	13	0	5	1	8	-	-
Relationships with other men	0	3	0	2	0	1	-	-
Bad friendships with other women	0	3	0	1	0	2	-	-
Financially defrauded	0	1	-	-	0	1	-	-
**Men would like their wives to use the mobile phone to**								
Transfer money	41	1 439	30	423	42	281	52	735
Buy/ pay for things	37	1 304	26	368	38	256	48	680

Table 3b. Women's sample characteristics by cluster based on women's baseline survey data from four districts of Madhya Pradesh

	Total n=3,484		Cluster 1 n=1,408		Cluster 2 n=666		Cluster 3 n=1,410	
	%	n	%	n	%	n	%	n
**Sociodemographic characteristics**								
**Socioeconomic status**								
Poorest	16	542	26	369	13	88	6	85
Poorer	19	646	27	379	18	117	11	150
Middle	20	710	22	313	25	167	16	230
Richer	22	760	15	214	25	165	27	381
Richest	24	826	9	133	19	129	40	564
**District**								
Hoshangabad	10	345	11	151	11	76	8	118
Mandsaur	19	676	13	181	14	95	28	400
Rajgarh	23	791	21	302	29	191	21	298
Rewa	48	1 672	55	774	46	304	42	594
**Mean age (years)**	72	3 484	25	1 408	23	666	24	1 410
**Ethnicity/Caste**								
General	22	780	17	242	19	129	29	409
OBC	49	1 690	45	628	48	321	53	741
Scheduled caste	19	647	23	322	21	140	13	185
Scheduled tribe	10	345	14	203	11	72	5	70
**Education**								
Never been to school	10	347	16	229	8	50	5	68
Primary school or less	18	610	23	327	17	114	12	169
Middle school	30	1 042	32	451	35	236	25	355
High school	34	1 168	26	363	33	223	41	582
Higher education	9	317	3	38	6	43	17	236
**Phone ownership and sharing**								
Own phone and do not share	51	1 781	43	609	38	256	65	916
Own phone and share	22	772	23	318	22	145	22	309
Share only	26	923	34	475	40	264	13	184
**Phone type (observed)**								
Brick phone	7	248	8	113	8	50	6	85
Feature phone	63	2 206	74	1 040	54	359	57	807
Smart phone	24	824	11	158	28	188	34	478
No phone observed	6	206	7	97	10	69	3	40
**Women's phone characteristics**								
**Phone features (observed)**								
Call	79	2 765	76	1 072	71	470	87	1 223

Speaker	79	2 762	76	1 072	71	470	87	1 220
SMS	79	2 768	76	1 074	71	471	87	1 223
Contacts	79	2 766	76	1 072	71	471	87	1 223
Camera	66	2 302	63	889	60	398	72	1 015
Music/ audio content	69	2 419	66	923	63	419	76	1 077
Internet	49	1 712	42	596	47	312	57	804
Bluetooth	64	2 243	60	842	59	390	72	1 011
Radio/FM	69	2 416	64	907	62	415	78	1 094
**Applications installed on phone (observed)**								
Facebook	25	859	17	237	23	156	33	466
WhatsApp	17	603	8	113	18	117	26	373
Shareit	10	364	4	61	11	71	16	232
**Proportion of phones with zero balance at time of interview**	48	1 666	47	655	50	334	48	677
**Who topped up credit?**								
Husband	80	2 784	79	1 109	81	537	81	1 138
Self	10	357	11	157	12	79	9	121
Other	10	343	10	142	8	50	11	151
**Frequency of most recent top-up**								
Within 1 week	21	718	24	343	19	125	18	250
Within 1 month	47	1 626	46	645	46	309	48	672
Within 3 months	24	841	21	299	23	155	27	387
More than 3 months	9	299	9	121	12	77	7	101
**Total amount of last top up**								
>50	55	1 902	59	831	47	311	54	760
0-50	45	1 582	41	577	53	355	46	650
**Women's phone use**								
**Digital skill (observed)**								
Able to navigate IVR prompts	69	2 409	81	1 142	87	578	90	1 275
Give a missed call	82	2 845	64	895	60	401	79	1 113
Store contacts on phone	47	1 654	73	1 021	83	555	90	1 269
Open SMS	32	1 102	33	471	39	263	65	920
Read SMS	32	1 102	18	255	26	171	48	676
Overall Basic Digital Skill Level	27	937	15	213	21	139	41	585
**Communication**	74	2 563	65	917	68	455	84	1 191
Call with spouse	73	2 542	81	905	80	454	89	1 183
Call with friends, relatives	43	1 485	83	478	87	297	82	710
Call with health workers	32	1 132	99	317	99	196	97	619
SMS with husband	16	545	97	103	99	91	96	351

SMS with friends, relatives	9	330	98	45	100	49	100	236
SMS with health workers	6	213	100	27	100	24	99	162
Dialled a number and listened to pre-recorded message	77	2 700	72	1 010	73	489	85	1 201
**Who taught respondent how to use phone?**								
Spouse	5	178	5	72	5	35	5	71
Self	72	2 512	70	986	71	472	75	1 054
Other	23	794	25	350	24	159	20	285

For peer review only

Supplementary Table 4. Strong signals (variable used for the spide charts are highlighted)

	Cluster 1 (n=1408)	Cluster 2 (n=666)	Cluster 3 (n=1410)
**Men paid for wife's balance**	37	0	90
**Men can perform basic internet search**	7	66	77
**Men report that their wife uses prepaid pack**	42	0	100
**Men report that women need their permission to add credit**	18	0	42
**Men report ever use of internet**	31	87	91
**Observe men watching Video**	42	93	95
**Men can send WhatsApp text**	3	77	85
**Men report use of WhatsApp**	7	91	95
**Men report that their wife's use the phone to**			
Search internet	12	36	55
Watch show	26	66	75
WhatsApp	11	37	57
Men report that they can send photo on WhatsApp	4	88	93
Men report that they can send a WhatsApp voice message	3	73	84
**Men report getting images and videos from**			
Internet: YouTube	19	84	88
Internet: Google	9	64	71
Other relatives	4	55	59
Friends locally	11	83	87
Friends other states	2	36	44
**Men report not using the internet frequently**	86	23	15
**Men have smart phone**	6	75	88
**Men report using the internet frequently**	14	77	85
**Men have feature phone**	68	23	9
**Number of phones in the household**			
3+	19	32	61
0-1	43	39	2
**Men report that their wife own's a phone**	42	0	100
**Men report that their wife does not own a phone**	58	100	0
**Men report their wife shares phone she owns with husband**	32	0	77
**Men observed to open WhatsApp**	6	91	94
**Men's observed digital literacy**	29	64	77
**Men observed to read SMS**	37	72	82
**Features men report using on their phone**			
Share photo	7	90	96
Search YouTube	21	98	98
Search Google	9	82	88
Download Apps	2	70	82
Make video	8	48	55
Share video	6	88	94
Watch video	51	99	99
WhatsApp	7	95	98
SMS	18	55	69
**Observe TikTok App on men's phone**	1	36	48
**Men have internet in their household**	25	54	69
**Men report women having a phone other than Samsung or Jio**	24	0	53

**Men report that women have a feature phone**	26	0	46

Supplementary Figure 1. PCA with 95% of cumulative explained variance on couples' data.

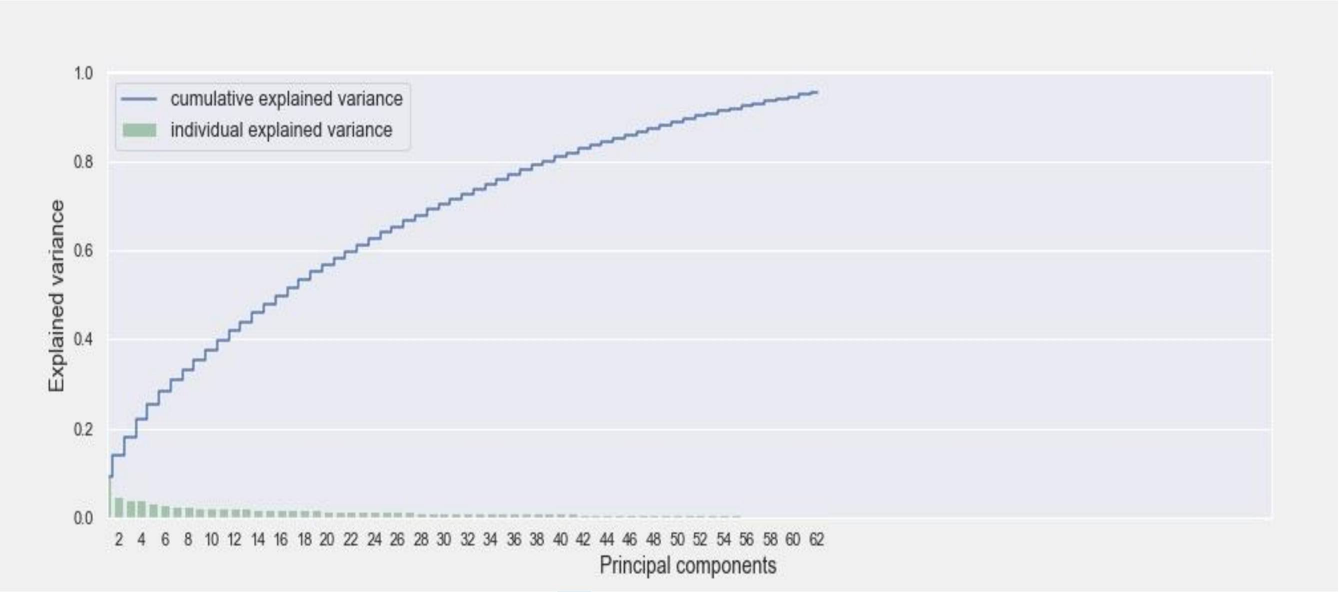

For peer review only

Reporting checklist for quality improvement in health care.

Based on the SQUIRE guidelines.

Instructions to authors

Complete this checklist by entering the page numbers from your manuscript where readers will find each of the items listed below.

Your article may not currently address all the items on the checklist. Please modify your text to include the missing information. If you are certain that an item does not apply, please write "n/a" and provide a short explanation.

Upload your completed checklist as an extra file when you submit to a journal.

In your methods section, say that you used the SQUIREreporting guidelines, and cite them as:

Ogrinc G, Davies L, Goodman D, Batalden P, Davidoff F, Stevens D. SQUIRE 2.0 (Standards for QUality Improvement Reporting Excellence): revised publication guidelines from a detailed consensus process

For peer review only

		Reporting Item	Page Number
**Title**			
	#1	Indicate that the manuscript concerns an initiative to improve healthcare (broadly defined to include the quality, safety,	1

		effectiveness, patientcenteredness, timeliness, cost, efficiency, and equity of healthcare)	
**Abstract**			3
	#02a	Provide adequate information to aid in searching and indexing	3
	#02b	Summarize all key information from various sections of the text using the abstract format of the intended publication or a structured summary such as: background, local problem, methods, interventions, results, conclusions	
**Introduction**			4
Problem description	#3	Nature and significance of the local problem	4
Available knowledge	#4	Summary of what is currently known about the problem, including relevant previous studies	4
Rationale	#5	Informal or formal frameworks, models, concepts, and / or theories used to explain the problem, any reasons or assumptions that were used to develop the intervention(s), and reasons why the intervention(s) was expected to work	4
Specific aims	#6	Purpose of the project and of this report	4
**Methods**			4
Context	#7	Contextual elements considered important at the outset of introducing the intervention(s)	5

For peer review only

Intervention(s)	#08a	Description of the intervention(s) in sufficient detail that others could reproduce it	5
Intervention(s)	#08b	Specifics of the team involved in the work	5
Study of the Intervention(s)	#09a	Approach chosen for assessing the impact of the intervention(s)	6
Study of the Intervention(s)	#09b	Approach used to establish whether the observed outcomes were due to the intervention(s)	6
Measures	#10a	Measures chosen for studying processes and outcomes of the intervention(s), including rationale for choosing them, their operational definitions, and their validity and reliability	6
Measures	#10b	Description of the approach to the ongoing assessment of contextual elements that contributed to the success, failure, efficiency, and cost	7
Measures	#10c	Methods employed for assessing completeness and accuracy of data	7
Analysis	#11a	Qualitative and quantitative methods used to draw inferences from the data	7
Analysis	#11b	Methods for understanding variation within the data, including the effects of time as a variable	7
Ethical considerations	#12	Ethical aspects of implementing and studying the intervention(s) and how they were addressed, including, but	NA

For peer review only

		not limited to, formal ethics review and potential conflict(s) of interest	
**Results**			7
	#13a	Initial steps of the intervention(s) and their evolution over time (e.g., time-line diagram, flow chart, or table), including modifications made to the intervention during the project	7
	#13b	Details of the process measures and outcome	8
	#13c	Contextual elements that interacted with the intervention(s)	8
	#13d	Observed associations between outcomes, interventions, and relevant contextual elements	9
	#13e	Unintended consequences such as unexpected benefits, problems, failures, or costs associated with the intervention(s).	NA
	#13f	Details about missing data	NA
**Discussion**			
Summary	#14a	Key findings, including relevance to the rationale and specific aims	10
Summary	#14b	Particular strengths of the project	10
Interpretation	#15a	Nature of the association between the intervention(s) and the outcomes	10
Interpretation	#15b	Comparison of results with findings from other publications	11

For peer review only

Interpretation	#15c	Impact of the project on people and systems	11
Interpretation	#15d	Reasons for any differences between observed and anticipated outcomes, including the influence of context	11
Interpretation	#15e	Costs and strategic trade-offs, including opportunity costs	11
Limitations	#16a	Limits to the generalizability of the work	11
Limitations	#16b	Factors that might have limited internal validity such as confounding, bias, or imprecision in the design, methods, measurement, or analysis	11
Limitations	#16c	Efforts made to minimize and adjust for limitations	11
Conclusion	#17a	Usefulness of the work	
Conclusion	#17b	Sustainability	11
Conclusion	#17c	Potential for spread to other contexts	12
Conclusion	#17d	Implications for practice and for further study in the field	12
Conclusion	#17e	Suggested next steps	12
**Other information**			12
Funding	#18	Sources of funding that supported this work. Role, if any, of the funding organization in the design, implementation, interpretation, and reporting	2

For peer review only

None The SQUIRE 2.0 checklist is distributed under the terms of the Creative Commons Attribution License CC BY-NC 4.0. This checklist can be completed online using https://www.goodreports.org/, a tool made by the EQUATOR Network in collaboration with Penelope.ai

For peer review only

BMJ Open

Can we design the next generation of digital health communication programs by leveraging the power of artificial intelligence to segment target audiences, bolster impact, and deliver differentiated services? A machine learning analysis of survey data from rural India

Journal:	*BMJ Open*
Manuscript ID	bmjopen-2022-063354.R2
Article Type:	Original research
Date Submitted by the Author:	27-Jan-2023
Complete List of Authors:	Bashingwa, Jean ; University of Cape Town Faculty of Health Sciences,<br>Mohan, Diwakar; Johns Hopkins University Bloomberg School of Public Health<br>Chamberlain, Sara; BBC Media Action, BBC Media Action, India; BBC Media Action, Asia<br>Scott, Kerry; Johns Hopkins University Bloomberg School of Public Health;<br>Ummer, Osama; Oxford Policy Management, ; BBC Media Action,<br>Godfrey, Anna; BBC Media Action,<br>Mulder, Nicola; University of Cape Town<br>Moodley, Deshen; University of Cape Town, Department of Computer Science<br>LeFevre, Amnesty; Johns Hopkins University, International Health
**Primary Subject Heading**:	Public health
Secondary Subject Heading:	Health services research
Keywords:	Public health < INFECTIOUS DISEASES, HEALTH ECONOMICS, Community child health < PAEDIATRICS, Information technology < BIOTECHNOLOGY & BIOINFORMATICS

SCHOLARONE™ Manuscripts

I, the Submitting Author has the right to grant and does grant on behalf of all authors of the Work (as defined in the below author licence), an exclusive licence and/or a non-exclusive licence for contributions from authors who are: i) UK Crown employees; ii) where BMJ has agreed a CC-BY licence shall apply, and/or iii) in accordance with the terms applicable for US Federal Government officers or employees acting as part of their official duties; on a worldwide, perpetual, irrevocable, royalty-free basis to BMJ Publishing Group Ltd ("BMJ") its licensees and where the relevant Journal is co-owned by BMJ to the co-owners of the Journal, to publish the Work in this journal and any other BMJ products and to exploit all rights, as set out in our licence.

The Submitting Author accepts and understands that any supply made under these terms is made by BMJ to the Submitting Author unless you are acting as an employee on behalf of your employer or a postgraduate student of an affiliated institution which is paying any applicable article publishing charge ("APC") for Open Access articles. Where the Submitting Author wishes to make the Work available on an Open Access basis (and intends to pay the relevant APC), the terms of reuse of such Open Access shall be governed by a Creative Commons licence – details of these licences and which Creative Commons licence will apply to this Work are set out in our licence referred to above.

Other than as permitted in any relevant BMJ Author's Self Archiving Policies, I confirm this Work has not been accepted for publication elsewhere, is not being considered for publication elsewhere and does not duplicate material already published. I confirm all authors consent to publication of this Work and authorise the granting of this licence.

For peer review only

Can we design the next generation of digital health communication programs by leveraging the power of artificial intelligence to segment target audiences, bolster impact, and deliver differentiated services? A machine learning analysis of survey data from rural India

Jean Juste Harrisson Bashingwa, PhD (corresponding author)
MRC/Wits-Agincourt Unit, School of Public Health, University of the Witwatersrand, 27 St. Andrews Road, Parktown, 2193, South Africa
Email: jeanjuste@aims.ac.za

Diwakar Mohan, DrPH
Department of International Health, Johns Hopkins Bloomberg School of Public Health, 615 N. Wolfe St, Baltimore, Maryland, USA
Email: dmohan3@jhu.edu

Sara Chamberlain, MA
Innov8 Old Fort Saket District Mall, Saket District Centre, Sector 6, Pushp Vihar, New Delhi, Delhi 110017, India
Email: sara.chamberlain@in.bbcmediaaction.org

Kerry Scott, PhD
Department of International Health, Johns Hopkins Bloomberg School of Public Health, 615 N. Wolfe St, Baltimore, Maryland, USA
Email: kscott26@jhu.edu

Osama Ummer, MHA
(1) BBC Media Action-India, Innov8 Old Fort Saket District Mall, Saket District Centre, Sector 6, Pushp Vihar, New Delhi, Delhi 110017, India
(2) Oxford Policy Management-Delhi, 4/6 First Floor, Siri Fort Institutional Area, New Delhi, Delhi 110049, India
Email: kposamaummer@gmail.com

Anna Godfrey, PhD
BBC Media Action, Ibex House, 42-47 Minories, London, EC3N 1DY, England
Email: anna.godfrey@bbc.co.uk

Nicola Mulder, PhD
Computational Biology Division, Department of Integrative Biomedical Sciences, Institute of Infectious Disease and Molecular Medicine, Faculty of Health Sciences, University of Cape Town
Anzio Road, Observatory, 7925, Cape Town, South Africa
Email: nicola.mulder@uct.ac.za

Deshen Moodley, PhD
Department of Computer Science,
18 University Avenue, University of Cape Town
Rondebosch, Cape Town, South Africa
Email: deshen@cs.uct.ac.za

For peer review only

Amnesty E. LeFevre PhD
1. School of Public Health and Family Medicine, University of Cape Town, Falmouth Rd, Observatory, Cape Town, 7925, South Africa
2. Department of International Health, Johns Hopkins Bloomberg School of Public Health, 615 N. Wolfe St, Baltimore, Maryland, USA
Email: aelefevre@gmail.com

Abstract (268 of 300 words)
Objectives
Direct to beneficiary (D2B) mobile health communication programs have been used to provide reproductive, maternal, neonatal and child health (RMNC) information to women and their families in a number of countries globally. Programs to date have provided the same content, at the same frequency, using the same channel to large beneficiary populations. This manuscript presents a proof of concept approach that uses machine learning to segment populations of women with access to phones and their husbands into distinct clusters to support differential digital program design and delivery.
Setting
Data used in this study were drawn from cross-sectional survey conducted in four districts of Madhya Pradesh, India.
Participants
Study participant included pregnant women with access to a phone (n=5,095) and their husbands (n=3,842)
Results
We used an iterative process involving K-means clustering and Lasso regression to segment couples into three distinct clusters. Cluster 1 (n=1,408) tended to be poorer, lessor educated men and women, with low levels of digital access and skills. Cluster 2 (n=666) had a mid-level of digital access and skills among men but not women. Cluster 3 (n=1,410) had high digital access and skill among men and moderate access and skills among women. Exposure to the D2B program 'Kilkari' showed the greatest difference in Cluster 2, including an 8% difference in use of reversible modern contraceptives, 7% in child immunisation at 10 weeks, 3% in child immunisation at 9 months, and 4% in the timeliness of immunisation at 10 weeks and 9 months.
Conclusions
Findings suggest that segmenting populations into distinct clusters for differentiated program design and delivery may serve to improve reach and impact.

Strengths and limitations of this study:
Strengths

- Segmenting populations into homogeneous groups can help to booster uptake of (D2B) mobile health communication programs.
- The step-wise approach combining K-means and Lasso regression is well superior compared to other approaches involving only either supervised or unsupervised machine learning to handle data from household surveys.

Limitations
- Our sample included men and women with a certain threshold of mobile phone access, possibly limiting the generalizability to populations with these characteristics.

For peer review only

- Survey data included a vast number of questions on mobile phone access and use, including observed digital skills, which to our knowledge are not widely available in India or elsewhere globally.
- K-means algorithm has certain limitations, including problems associated with random initialization of the centroids which leads to unexpected convergence.

Introduction

Digital health solutions have the potential to address critical gaps in information access and service delivery, which underpin high mortality [1-9]. Mobile health communication programs, which provide information directly to beneficiaries, are among the few examples of digital health solutions to have scaled widely in a range of settings [10, 11]. Historically, these solutions have been designed as 'blunt instruments' – providing the same content, with the same frequency, using the same digital channel to large target populations. While this approach has enabled solutions to scale, it has contributed to variability in their reach and impact, due in part to differences in women's access to and use of mobile phones, particularly in low- and middle-income countries [12, 13].

Despite near ubiquitous ownership of mobile phones at a household level, a growing body of evidence suggests that there is a substantial gap between men and women's ownership, access to and use of mobile phones [14-16]. In India, there is a 45% gap between women's reported access to a phone and ownership at a household level [16]. Variations in the size of the gap have been observed across states and urban/rural areas, and by sociodemographic characteristics, including education, caste, and socioeconomic status [16]. Amongst women with reported access to a mobile phone, the gender gap further persists in the use of mobiles, in part because of patriarchal gender norms and limited digital skills [17]. Collectively, these gender gaps underscore the need to consider inequities in phone access and use patterns when designing and implementing D2B mobile health communication programs.

Kilkari, designed and scaled by BBC Media Action in collaboration with the Ministry of Health and Family Welfare, is India's largest direct to beneficiary mobile health information program. When BBC Media Action transitioned Kilkari to the national government in April 2019, it had been implemented in 13 states and reached over 10 million women and their families [3, 18, 19] . Evidence on the program's impact from a randomized control trial conducted in Madhya Pradesh, India, between 2018 and 2021, suggests that across study arms, Kilkari was associated with a 3.7% increase in modern reversible contraceptive use (RR: 1.12, 95% CI: 1.03 to 1.21, p=0.007), and a 2.0% decrease in the proportion of male or females sterilized since the birth of the child (RR: 0.85, 95% CI: 0.74 to 0.97, p=0.016) [3, 19]. The program's impact on contraceptive use, however, varied across key population sub-groups. Among women exposed to 50% or more of the Kilkari content as compared to those not exposed, differences in reversible method use were greatest for those in the poorest socioeconomic strata (15.8% higher), for those in disadvantaged castes (12.0% higher), and for those with any male child (9.9% higher) [3, 19]. Kilkari's overall and varied impact across beneficiary groups raises important questions about whether the differential targeting of women and their families might lead to efficiency gains and deepen impact.

In this manuscript, we argue that to maximize reach, exposure, and deepen impact, the future design of mobile health communication solutions will need to consider the heterogeneity of beneficiaries, including within husband-wife couples, and move away from a one-size-fits all model towards differentiated program design and delivery. Drawing from husbands' and wives' survey data captured as part of a randomised controlled trial of Kilkari in Madhya Pradesh India, we used a three-step process involving K-means clustering and Lasso (Least Absolute Shrinkage and Selection Operator) regression to segment couples into distinct clusters. We then assess differences in health behaviours across respondents in both study arms of the RCT. Findings are anticipated to inform future efforts to capture data and refine methods for segmenting

For peer review only - http://bmjopen.bmj.com/site/about/guidelines.xhtml

beneficiary populations and in turn optimizing the design and delivery of mobile health communication programs in India and elsewhere globally.

Methods

Kilkari program overview

Kilkari is an outbound service that makes weekly, stage-based, pre-recorded calls about reproductive, maternal, neonatal and child health (RMNCH) directly to families' mobile phones, starting from the second trimester of pregnancy until the child is one year old. Kilkari is comprised of 90 minutes of reproductive, maternal, newborn and child health content sent via 72 once weekly voice calls (average call duration: 1 minute, 15 seconds). Approximately 18% of cumulative call content is on family planning; 13% on child immunisation; 13% on nutrition; 12% on infant feeding; 10% on pregnancy care; 7% on entitlements; 7% on diarrhoea; 7% on postnatal care; and the remainder on a range of topics including intrapartum care, water and sanitation (WASH), and early childhood development. BBC Media Action designed and piloted Kilkari in the Indian state of Bihar in 2012-2013, and then redesigned and scaled it in collaboration with the Ministry of Health and Family Welfare between 2015 and 2019. Evidence on the evaluation design and program impact are reported elsewhere [20].

Setting

Data used in this analysis were collected from four districts of the central Indian state of Madhya Pradesh as part of the impact evaluation of Kilkari described elsewhere [3, 19]. Madhya Pradesh (population 75 million) is home to an estimated 20% of India's population and falls below national averages for most sociodemographic and health indicators [21]. Wide differences by gender and between urban and rural areas persist for wide range of indicators including literacy, phone access and health seeking behaviours. Among men and women 15-49 years of age, 59% of women (78% urban and 51% rural) were literate as compared to 82% of men in 2015-2016 [21]. Amongst literate women, 23% had 10 or more years of schooling (44% urban and 14% rural) [21]. Despite near universal access to phones at a household level, only 19% of women in rural areas and 50% in urban had access to a phone that they themselves could use in 2015 [21]. Among pregnant women, over half (52%) of pregnant women received the recommended four ANC visits in urban areas as compared to only 30% in rural areas [21]. Despite high rates of institutional delivery (94%) in urban areas, only 76% of women in rural areas reported delivering in a health facility in 2015 [21]. These disparities underscore the population heterogeneity within and across Madhya Pradesh.

Sample population

The sample for this study were obtained through cross-sectional surveys administered between 2018 and 2020 to women (n=5,095) with access to a mobile phone and their husbands (n=3,842) in four districts of Madhya Pradesh [20]. At the time of the first survey (2018-2019), the women were 4-7 months pregnant; the latter survey (2019-2020) re-interviewed the same women at 12 months postpartum. Their husbands were only interviewed once, during the latter survey round. The surveys spanned 1.5 hours in length. In this analysis, modules on household assets and member characteristics; phone access and use, including observed digital skills (navigate IVR prompts, give a missed call, store contacts on a phone, open SMS, read SMS) were used to develop models. Data on practice for maternal and child health behaviours, including infant and young child feeding, family planning, pregnancy and postpartum care were used to explore the differential impact of Kilkari across clusters but not used in the development of clusters [20].

Approach to segmentation

Figure 1 presents a framework used for developing homogenous clusters of men and women in four districts of rural Madhya Pradesh India. Box 1 describes the steps undertaken at each point in the framework in detail. We started with data elements collected on phone access and use as well as population sociodemographic characteristics collected as part of a cross-sectional survey described elsewhere[3, 22]. Unsupervised learning was undertaken using K-Means cluster and strong signals were identified. Strong signals were defined as variables that had at least a prevalence of 70% in one or more clusters and differed

For peer review only - http://bmjopen.bmj.com/site/about/guidelines.xhtml

from another cluster by 50% or more. For example, 6% of men own a smart phone in cluster 1, 88% in cluster 2 and 75% in cluster 3. Therefore, having a smart phone can be considered as a strong signal. Additional details are summarised in Box 1. Once defined, we then explored differences in health care practices across study clusters among those exposed and not exposed to Kilkari within each cluster.

Patient and public involvement

Patients were first engaged upon identification in their households as part of a household listing carried out in mid/ late 2018. Those meeting eligibility criteria were interviewed as part of the baseline survey, and ultimately randomized to the intervention and control arms. Prior to the administration of the baseline, a small number of patients were involved in the refinement of survey tools through qualitative interviews, including cognitive interviews, which were carried out to optimise survey questions, including the language and translation used. Finalised tools were administered to patients at baseline and endline, and for a sub-sample of the study population, additional interviews carried out over the phone and via qualitative interviews between the baseline and endline surveys. Unfortunately, because of COVID-19 patients and associated travel restrictions could not be involved in the dissemination of study findings.

For peer review only

Box 1. Step-wise process for developing and refining a machine learning approach for population segmentation

Data collected from special surveys like the couple's data set used here are relatively smaller in terms of sample size but large with regard to the number of data elements available. In such high dimensional data, there are many irrelevant dimensions which can mask existing clusters in noisy data, making more difficult the development of effective clustering methods [3, 23]. Several approaches have been proposed to address this problem. They can be grouped into two categories: static or adaptive *dimensionality reduction*, including principal components analysis (PCA) [24, 25] and *subspace clustering* consisting on selecting a small number of original dimensions (features) in some unsupervised way or using expert knowledge so that clusters become more obvious in the subspace [26, 27] . In this study we combined subspace clustering using expert knowledge and adaptive dimensionality reduction (Supplementary Figure 1) to find subspace where clusters are most well separated and well defined. Therefore, as part of subspace clustering, we chose to start with couples' survey data, including variables related to socio demographic characteristic, phone ownership, use and literacy (Supplementary Table 1). Emergent clusters were overlapping. We decided to use men's survey data on phone access and use as a starting point.

Step 1. Defining variables which characterise homogenous groups

Analyses started with a predefined set of data elements captured as part of a men's cross-sectional survey including sociodemographic characteristics and phone access and use. K-Means clustering was used to identify clusters and the elbow method was used to define the optimal number of clusters. Strong signals were then identified. Variables which had at least a prevalence of 70% in one or more clusters and differed from another cluster by 50% or more were considered to have a strong signal.

Step 2. Model strengthen through the identification and addition of new variables

Once an initial model was developed drawing from the predefined set of data from the men's survey and strong signals were identified, we reviewed available data from the combined dataset (data from the men's survey and women's survey). Signal strength was used as an outcome variable or target in a linear regression with L1 regularization or Lasso regression (Least Absolute Shrinkage and Selection Operator). Regularization is a technique used in supervised learning to avoid overfitting. Lasso Regression adds absolute value of magnitude of coefficient as penalty term to the loss function. The loss function becomes:

$$Loss = Error(y,y) + \alpha\sum_{i=1}^{N}|\omega_i|$$

where ω_i are coefficients of linear regression $y = \omega_1 x_1 + \omega_2 x_2 + ... + \omega_N x_N + b$

Lasso Regression works well for selecting features in very large datasets as it shrinks the less important features of coefficients to zero [28, 29]. Merged women's survey and men's survey data were used as predictors for the regression, excluding variables related to heath knowledge and practices. We ended up with a sample of 3,484 rows and 1,725 variables after data pre-processing.

Step 3. Refining clusters using supervised learning

We then re-ran K-Means clustering with three clusters (K=3) using important features selected by Lasso regression. This methodology was used to refine the clusters and subsequently identify new strong signals. After step 3 was conducted, we repeated step 2, and kept on iteratively repeating step 2 and 3 until there was no gain in strong signals. Data preparation and results formatting have been conducted in R 4.1.1 [30], K-means clustering has been performed in python 3.8.5 [31].

For peer review only - http://bmjopen.bmj.com/site/about/guidelines.xhtml

Figure 1. Framework for segmentation analysis

K-Means algorithm

As part of Steps 1 and 3, K-means algorithms were used (Box 1). We chose to use K-means algorithm because of its simplicity and speed to handle large dataset compared to hierarchical clustering [32]. A K-Means algorithm is one method of cluster analysis designed to uncover natural groupings within a heterogeneous population by minimizing Euclidean distance between them [33]. When using a K-Means algorithm, the first step is to choose the number of clusters K that will be generated. The algorithm starts by selecting K points randomly as the initial centres (also known as cluster means or centroids) and then iteratively assigns each observation to the nearest centre. Next, the algorithm computes the new mean value (centroid) of each cluster's new set of observation. K-Means re-iterates this process, assigning observations to the nearest centre. This process repeats until a new iteration no longer reassigns any observations to a new cluster (convergence). Four metrics have been used for the validation of clustering: within cluster sum of squares, silhouette index, Ray-Turi criterion and Calinski-Harabatz criterion. Elbow method was used to find the right K (number of clusters) [34]. Figure 2 is a chart showing the within cluster sum of squares (or inertia) by the number of groups (k value) chosen for several executions of the algorithm.

Figure 2. Elbow method used to help decide ultimate number of clusters appropriate for the data.

Inertia is a metric that shows how dissimilar the members of a group are. The less inertia there is, the more similarity there is within a cluster (compactness). The main purpose of clustering is not to find 100% compactness, it is rather to find a fair number of groups that could explain with satisfaction a considerable part of the data (k=3 in this case). Silhouette analysis helped to evaluate the goodness of clustering or clustering validation (*Figure 3)*. It can be used to study the separation distance between the resulting clusters. The silhouette plot displays a measure of how close each point in one cluster is to points in the neighbouring clusters. This measure has a range of [-1, 1]. Silhouette coefficients near +1 indicate that the sample is far from the neighbouring clusters. A value of 0 indicates that the sample is very close to the decision boundary between two neighbouring clusters and negative values indicate that those samples might have been assigned to the wrong cluster. Figure 3 shows that choosing three clusters was more efficient than four for the data from the available surveys for two reasons: 1) there were less points with negative silhouettes, 2) the cluster size (thickness) was more uniform for three groupings. Other criterions used to evaluate quality of clustering are obtained by combining the 'within cluster compactness index' and 'between-cluster spacing index' [35]. Calinski-Harabatz criterion is given by: $C(k) = \frac{Trace(B)\ (n-k)}{Trace(W)\ (k-1)}$ and Ray-Turi criterion is given by $r(k) = \frac{distance\ (W)}{distance\ (B)}$ where B is the between-cluster covariance matrix (so high values of B denote well-separated clusters) and W is the within-cluster covariance matrix (so low values of W correspond to compact clusters) . They both ended up with same conclusions that 3 clusters were the best choice for the data we had. Supplementary Table 2 gives different metrics used and values obtained for various clusters.

Figure 3. Silhouette analysis for three and four clusters

Results

Sample characteristics

Supplementary Tables 3a and 3b summarise the sample characteristics by cluster for men and women interviewed. Figure 4 and Supplementary Table 4 presents select characteristics with 'strong signals' for each cluster.

For peer review only - http://bmjopen.bmj.com/site/about/guidelines.xhtml

Cluster 1 (n=1,408) constitutes 40% of the sample population and was comprised of men and women with low levels of digital access and skills (Figure 4). This cluster included the poorest segment of the sample population: 36% had a primary school or lower education and 40% were from a scheduled tribe/caste. Most men owned a feature (68%) or brick phone (22%); used the phone daily (89%); and while able to navigate IVR prompts (91%), only 29% were able to perform all of the five basic digital skills assessed. Women in this cluster similarly had lower levels of education as compared to other clusters (39% have primary school or less education); used feature (74%) or brick phones (8%); and had low digital skills (15% were able to perform the five basic digital skills assessed).

Cluster 2 (n=666; 19% of sample population), is comprised of men with mid-level and women with low digital access and skills. In this cluster, 75% of men owned smartphones, 65% were observed to successfully perform the five basic digital skills assessed, and 36% could perform a basic internet search. Men in Cluster 2 also self-reported accessing videos from YouTube (84%) and using WhatsApp (95%). Women in Cluster 2 had low phone ownership; nearly half of women reported owning a phone (38% owned a phone and did not share it, 22% owned and shared a phone) — findings which contradict their husbands' reports of 0% women's phone ownership. Only 21% of women in this cluster were observed to be able to successfully perform the five basic digital skills assessed. However, based on husband's reporting of their wives' digital skills, 36% of women could search the internet, 37% used WhatsApp, and 66% watched shows on someone else's phone.

Cluster 3 (n=1,410; 40% of sample population) is comprised of couples with high level digital access among both husbands and wives, and lower-level digital skill among wives (Figure 4). An estimated 67% of couples in this cluster were in the richer or richest socioeconomic strata, while 71% of men and 58% of women had high school or higher levels of education. Men in this cluster reported using the internet frequently (85%), were observed to own smart phones (88%), and had high levels of digital skills: 77% could perform the five basic digital skills assessed, 77% could perform a basic internet search, and 85% could send a WhatsApp message When reporting on their wife's digital access and skills, all men in this cluster reported that their wives' owned phones (100%), but often shared these phones with their husbands (77%), using them to watch shows (75%), search the internet (55%), or use WhatsApp (57%). However, a much lower level of women interviewed in this cluster were observed to own Feature (57%) or Smart phones (34%) and had moderate digital skills with 41% being able to successfully perform the five basic digital skills assessed.

Figure 4. Distribution of select characteristics with strong signals by Cluster

Differences in health outcomes by Cluster

Table 1 presents differences in health outcomes by Cluster among those exposed and not exposed to Kilkari as part of the randomised controlled trial in Madhya Pradesh. Findings suggest that the greatest impact was observed among those exposed to Kilkari in Cluster 2, which is the smallest cluster identified (19% of the sample population). Amongst this population, differences between exposed and not exposed were 8% for reversible modern contraceptive methods, 7% for immunisation at 10 weeks, 3% for immunisation at 9 months, and 4% for timely immunisation at 10 weeks and 9 months. Additionally, an 8% difference between exposed and not exposed was observed for the proportion of women who report being involved in the decision about what complementary foods to give child.

Among Clusters 1 and 3, improvements were observed among those exposed to Kilkari for a small number of outcomes. In Cluster 1, those exposed to Kilkari had a 3-4% higher rate of immunisation at 6, 10, 14 weeks than those not exposed. In both Clusters 1 and 3 the timeliness of immunisation improved at 10

For peer review only

weeks amongst those exposed. No improvements were observed for use of modern reversible contraception in either cluster.

For peer review only

Table 1. Differential impact of Kilkari exposure on family planning, infant feeding and immunizations per cluster

	Cluster1						Cluster2						Cluster3					
	Not exposed			Exposed			Not exposed			Exposed			Not exposed			Exposed		
	%	N	SE	%	N	SE	%	N	SE	%	N	SE	%	N	SE	%	N	SE
**Family planning**																		
Current modern family planning use	42	269	0.02	41	316	0.018	42	130	0.028	44	157	0.026	50	340	0.019	51	368	0.019
Reversible methods	29	183	0.018	30	232	0.017	30	94	0.026	38	133	0.026	41	280	0.019	44	319	0.018
Sterilized	12	77	0.013	10	80	0.011	11	33	0.017	8	30	0.015	10	66	0.011	7	54	0.01
Sterilized	18	114	0.015	16	121	0.013	15	47	0.02	12	44	0.018	14	99	0.013	12	84	0.012
**Infant and young child feeding**																		
Immediate breastfeeding	96	610	0.008	95	736	0.008	93	291	0.014	95	336	0.012	94	645	0.009	93	675	0.009
Gave child semi solid food yesterday	98	624	0.005	99	762	0.004	99	309	0.006	99	350	0.006	99	676	0.004	98	715	0.005
Exclusive breastfeeding	6	39	0.01	6	48	0.009	7	21	0.014	8	28	0.014	6	43	0.009	7	51	0.009
Fed child solid, semi-solid or soft foods the minimum number of times during the previous day	54	344	0.02	55	423	0.018	62	193	0.028	64	228	0.025	66	450	0.018	65	469	0.018
Minimum acceptable diet	27	171	0.018	28	219	0.016	29	91	0.026	26	92	0.023	25	170	0.017	27	198	0.017
Women involved in the decision about what complementary foods to give child	89	569	0.012	92	708	0.01	82	256	0.022	90	319	0.016	88	604	0.012	87	634	0.012
**Immunization**																		
Fully immunized	44	280	0.02	44	340	0.018	45	139	0.028	49	173	0.027	51	350	0.019	48	352	0.019
Birth	70	444	0.018	70	542	0.016	71	223	0.026	73	259	0.024	72	493	0.017	74	534	0.016
6 weeks	75	475	0.017	78	600	0.015	78	242	0.024	79	280	0.022	77	528	0.016	78	568	0.015
10 weeks	72	460	0.018	76	584	0.015	72	225	0.025	79	279	0.022	75	514	0.017	76	554	0.016
14 weeks	68	432	0.019	71	550	0.016	74	230	0.025	74	263	0.023	75	511	0.017	75	541	0.016
9 months	68	433	0.018	68	522	0.017	69	214	0.026	72	255	0.024	75	510	0.017	74	538	0.016
Timeliness: birth	69	438	0.018	67	515	0.017	68	213	0.026	69	246	0.025	70	477	0.018	72	525	0.017
Timeliness: 6 weeks	45	287	0.02	46	353	0.018	45	139	0.028	44	155	0.026	51	349	0.019	51	371	0.019
Timeliness: 10 weeks	25	162	0.017	28	217	0.016	23	71	0.024	27	94	0.024	31	213	0.018	34	248	0.018
Timeliness: 14 weeks	13	85	0.014	13	102	0.012	14	43	0.02	14	51	0.019	19	131	0.015	22	162	0.015
Timeliness: 9 months	14	89	0.014	13	99	0.012	12	37	0.018	16	55	0.019	18	126	0.015	17	126	0.014

Discussion

Evidence on the impact of direct to beneficiary mobile health communication programs is limited but broadly suggests that they can cost-effectively improve some reproductive, maternal and child health practices. This analysis aims to serve as a proof of concept for segmenting beneficiary populations to support the design of more targeted mobile health communication programs. We used a three-step iterative process involving a combination of supervised and unsupervised learning (K-means clustering and Lasso regression) to segment couples into distinct clusters. Three identifiable groups emerge each with differing health behaviours. Findings suggest that exposure the D2B program Kilkari may have a differential impact among the clusters.

Implications for designing future digital solutions

Findings demonstrate that the impact of the D2B solution Kilkari varied across homogenous clusters of women with access to mobile phones and their husbands in Madhya Pradesh. Across delivery channels, our analysis indicates that mobile health communication could not be effectively delivered to husbands and wives in Cluster 1 using WhatsApp, because smartphone ownership and WhatsApp use in this cluster are negligible. IVR, on the other hand, could be used to reach couples in Cluster 1, but reach is likely to be sporadic because of high levels of phone sharing with others (78% among men and 57% among women). On the other hand, WhatsApp and YouTube are likely to be effective digital channels for communicating with both husbands and wives in Cluster 3, where most men and women own or use smartphones and WhatsApp.

Beyond delivery channels, study findings raise a number of important learnings for content development as well as optimising beneficiary reach and exposure. The creative approach to content created for Cluster 3, where 40% of women are from the richest socio-economic status and only 17% have never been to school or have a Primary School education or less, would need to be very different from the creative approach to content created for Cluster 1, where 53% have a poorest or poorer socio-economic status, and 39% have never been to school or have a Primary School education or less. Similarly, this analysis adds to qualitative findings [17] and provides important insights into how gender norms related to women's use of mobile phones may effect reach and impact. While few (13-15%) husbands indicated that 'adults' need oversight to use mobile phones, men's perceptions varied when asked about specific use cases. Across all Clusters, nearly half of husbands indicated that their wives needed permission to pick up phone calls from unknown numbers – an important insight for IVR programs which may make outbound calls without pre-warning to beneficiaries. In Clusters 1 and 2, 25% and 29% of husband's, respectively, report that their wives need permission to answer calls from health workers – as compared to 15% in Cluster 3. While restrictions on SMS and WhatsApp were lower than making or receiving calls, these channels are less viable given women's limited access to smartphones, low literacy and digital skills. Overall, men's perceptions on the restrictions needed on the receipt and placement of calls by women was lower for Cluster 3. However, despite the relative wealth of beneficiaries in Cluster 3 (67% were in the richer or richest socioeconomic strata), 48% of women had zero balance on their mobile phones at the time of interview. Collectively, these findings highlight the immense challenges which underpin efforts to facilitate women's phone access and use. They too underline the criticality of designing mobile health communication content for couples, rather than just wives to ensure the buy-in of male gatekeepers, and for continuing to prioritize face to face communication with women on critical health issues.

Approach to segmentation

Data in our sample were captured as part of special surveys carried out through the impact evaluation of Kilkari. Future programs may be tempted to apply the approach undertaken here to existing datasets, including routine health information systems or other forms of government tracking data. In the India context, while these data are likely to be less costly than special surveys, they are comparatively limited in terms of data elements captured – particularly in terms of data ownership of different types of mobile devices, digital skill levels and usage of specific applications or social media platforms. Data quality may

For peer review only - http://bmjopen.bmj.com/site/about/guidelines.xhtml

also be a significant issue in existing datasets . For example, we estimate that SIM change in our study population was 44% over a 12-month period – a factor which when coupled with the absence of systems to update government tracking registries raises important questions about who is retained in these databases, and therefore able to receive mobile health communications—and who is missing. Amongst the variables used, men's phone access and use were most integral to developing distinct clusters. We recommend that future surveys seeking to generate data for designing digital services for women ensure that data elements are captured on men's phone access and use practices as well as their perception of their wife's phone access and use.

In addition to underlying data, our analytic approach differed from other segmentation analyses. . Our work is relatively new in global health literature related to digital health programs that are positioned as D2B programs. While similar ML models are being tested in various domains related to public health, they consist exclusively of unsupervised learning [36, 37] or supervised learning [1, 6, 38, 39], this analysis is the first of its kind focusing on the use of a combination of supervised and unsupervised learning to identify homogenous clusters for targeting of digital health programs. Data collected from special surveys like the couple's data set used here are comparatively smaller in terms of sample size but large with regard to the number of data elements available. An alternative approach to that described in this manuscript might be to develop strata based on population characteristics. Indeed, findings from the impact evaluation published elsewhere suggest that women with access to phones in the most disadvantaged sociodemographic strata (poorest (15.8% higher) and disadvantaged castes (12% higher)) had greater impact when exposed to 50% or more of the Kilkari content as compared to those not exposed. With an approach to segmentation based on these strata of highest impact, we know and understand what divides or groups respondents (e.g. socioeconomic status, education) but this may not be enough when they do not explain the underlying reasons for change. In the approach used here, the study population is segmented using multiple characteristics (sociodemographic, digital access and use) simultaneously. The results are clusters comprised of individuals with mixed sociodemographic characteristics which may help to explain the reduced impact observed on health outcomes. Designing a strategy based on previously known / identifiable strata alone has been the basis of targeting in public health but has not maximized reach, exposure and effect to its fullest potential. The approach used here may better group beneficiaries based on their digital access and use characteristics which may serve to increase reach and exposure. However, further research is needed to determine how to deepen impact within these digital clusters.

Conclusions

Study findings sought to identify distinct clusters of husbands and wives based on their sociodemographic, phone access and use characteristics, and to explore the differential impact of a maternal mobile messaging program across these clusters. Three identifiable groups emerge each with differing levels of digital access and use. Descriptive analyses suggest that improvements in some health behaviours were observed for a greater number of outcomes in Cluster 2, than in Clusters 1 and 3. These findings suggest that one size fits all mobile health communications solutions may only engage one segment of a target beneficiary population, and offer much promise for future direct to beneficiary and other digital health programs which could see greater reach, exposure and impact through differentiated design and implementation. More quantitative and qualitative work is needed to better understand factors driving the differences in impact and what is likely to motivate adoption of target behaviours in different clusters. Our work opens up a new avenue of research into better targeting of beneficiaries using data on variety of domains including socio-demographics, mobile phone access and use. Future work will entail evaluation of the actual platform used for targeting and delivery of the program in pilot projects. Successful pilots can be scaled up to larger swathes of the population in India and similar setting around the world.

For peer review only

Acknowledgments: We thank the women and families of Madhya Pradesh who generously gave of their time to support this work. We are humbled by the opportunity to convey their perspectives and experiences. We additionally are grateful to Dr. Rajani Ved at the National Health Systems Resource Centre for her support. This work was made possible by the Bill and Melinda Gates Foundation. We thank Diva Dhar, Suhel Bidani, Rahul Mullick, Dr. Suneeta Krishnan, Dr. Neeta Goel and Dr. Priya Nanda for believing in us and giving us this opportunity. We additionally wish to thank BBC Media Action teams in India and London for their partnership and collaboration. The evaluation was unquestionably strengthened by their support, transparency, and willingness to work with us on all facets of the research. We too are grateful to the larger team of enumerators from OPM-India who worked tirelessly over many months to implement the surveys that form the backbone of our analyses. We additionally thank Prabal Singh, Vinit Pattnaik at OPM and Alain Labrique, Smisha Agarwal, and Erica Crawford at Johns Hopkins University for their support. Lastly, our figures have been beautified by the great and ever patient Dan Harder of the Creativity Club UK. We thank him for his work.

Contributions: JJHB conducted the analysis and wrote the paper with AEL and inputs from DM, SC, and other authors. AEL is the overall study PI, helped to secure the funding, led the design of the study tools, supported oversight of field work and analysis, and wrote the manuscript with JJHB and DM. DM helped to secure funding, helmed the study design including sampling and randomisation, helped draft study tools, provided input to data analysis, and edited the manuscript. SC helped to secure the funding, draft and review study tools, interpret data analyses and study findings, and edit the manuscript. AG, KS, helped to draft and review study tools, interpret data analyses and study findings, and edit the manuscript. OU help to revise study tools, interpret data analyses, and edited the manuscript. NM is the UCT study PI and provided input to study design, oversight to the analysis and interpretation, and edited the manuscript.

Competing interests: All authors have completed the Unified Competing Interest form (available on request from the corresponding author) and declare that the research reported was funded by the Bill and Melinda Gates Foundation. AG and SC are employed by BBC Media Action; one of the entities supporting program implementation. The authors do not have other relationships and are not engaged in activities that could appear to have influenced the submitted work.

Funding: Bill and Melinda Gates Foundation grant number OPP1179252

Data sharing: The anonymised raw data are available upon request.

Ethics: Institutional Review Boards from the Johns Hopkins Bloomberg School of Public Health in Baltimore, Maryland USA and Sigma Research and Consulting in Delhi, India provided ethical clearance for study activities. Verbal informed consent was obtained from all study participants.

References

[1] A.K. Dey, N. Dehingia, N. Bhan, E.E. Thomas, L. McDougal, S. Averbach, J. McAuley, A. Singh, A.J.S.-P.H. Raj, Using machine learning to understand determinants of IUD use in India: Analyses of the National Family Health Surveys (NFHS-4), 19 (2022) 101234.

[2] N.U.Z. Khan, S. Rasheed, T. Sharmin, A. Siddique, M. Dibley, A.J.B.h.s.r. Alam, How can mobile phones be used to improve nutrition service delivery in rural Bangladesh?, 18(1) (2018) 1-10.

For peer review only

[3] A.E. LeFevre, N. Shah, K. Scott, S. Chamberlain, O. Ummer, J.J.H. Bashingwa, A. Chakraborty, A. Godfrey, P. Dutt, R.J.B.g.h. Ved, The impact of a direct to beneficiary mobile communication program on reproductive and child health outcomes: a randomised controlled trial in India, 6(Suppl 5) (2022) e008838.
[4] D. Mohan, J.J.H. Bashingwa, K. Scott, S. Arora, S. Rahul, N. Mulder, S. Chamberlain, A.E.J.B.g.h. LeFevre, Optimising the reach of mobile health messaging programmes: an analysis of system generated data for the Kilkari programme across 13 states in India, 6(Suppl 5) (2022) e009395.
[5] M. Njoroge, D. Zurovac, E.A. Ogara, J. Chuma, D.J.B.r.n. Kirigia, Assessing the feasibility of eHealth and mHealth: a systematic review and analysis of initiatives implemented in Kenya, 10(1) (2017) 1-11.
[6] A. Raj, N. Dehingia, A. Singh, L. McDougal, J.J.S.-p.h. McAuley, Application of machine learning to understand child marriage in India, 12 (2020) 100687.
[7] S. Siddique, J.C.J.E. Chow, Machine learning in healthcare communication, 1(1) (2021) 220-239.
[8] M. Deshmukh, P.J.W. Mechael, DC: mHealth Alliance, Addressing gender and women's empowerment in mHealth for MNCH: An analytical framework, (2013).
[9] S. Lund, M. Hemed, B.B. Nielsen, A. Said, K. Said, M. Makungu, V.J.B.A.I.J.o.O. Rasch, Gynaecology, Mobile phones as a health communication tool to improve skilled attendance at delivery in Zanzibar: a cluster-randomised controlled trial, 119(10) (2012) 1256-1264.
[10] J.J.H. Bashingwa, D. Mohan, S. Chamberlain, S. Arora, J. Mendiratta, S. Rahul, V. Chauhan, K. Scott, N. Shah, O.J.B.G.H. Ummer, Assessing exposure to Kilkari: a big data analysis of a large maternal mobile messaging service across 13 states in India, 6(Suppl 5) (2021) e005213.
[11] J.J.H. Bashingwa, N. Shah, D. Mohan, K. Scott, S. Chamberlain, N. Mulder, S. Rahul, S. Arora, A. Chakraborty, O.J.B.g.h. Ummer, Examining the reach and exposure of a mobile phone-based training programme for frontline health workers (ASHAs) in 13 states across India, 6(Suppl 5) (2021) e005299.
[12] A. LeFevre, S. Chamberlain, N. Singh, K. Scott, P. Menon, P. Barron, R. Ved, A. George, Avoiding the Road to Nowhere: Policy Insights on Scaling up and Sustaining Digital Health, Global Policy (2021).
[13] A. Swartz, A.E. LeFevre, S. Perera, M.V. Kinney, A.S. George, Multiple pathways to scaling up and sustainability: an exploration of digital health solutions in South Africa, Global Health 17(1) (2021) 77.
[14] GSMA, Connected women: The mobile gender gap report 2020, GSM Association (2020).
[15] A.E. LeFevre, N. Shah, J.J.H. Bashingwa, A.S. George, D. Mohan, Does women's mobile phone ownership matter for health? Evidence from 15 countries, BMJ Glob Health 5(5) (2020).
[16] D. Mohan, J.J.H. Bashingwa, N. Tiffin, D. Dhar, N. Mulder, A. George, A.E. LeFevre, Does having a mobile phone matter? Linking phone access among women to health in India: An exploratory analysis of the National Family Health Survey, PLoS One 15(7) (2020) e0236078.
[17] K. Scott, O. Ummer, A. Shinde, M. Sharma, S. Yadav, A. Jairath, N. Purty, N. Shah, D. Mohan, S.J.B.G.H. Chamberlain, Another voice in the crowd: the challenge of changing family planning and child feeding practices through mHealth messaging in rural central India, 6(Suppl 5) (2021) e005868.

For peer review only

[18] D. Mohan, J.J.H. Bashingwa, P. Dane, S. Chamberlain, N. Tiffin, A.J.J.r.p. Lefevre, Use of big data and machine learning methods in the monitoring and evaluation of digital health programs in India: An exploratory protocol, 8(5) (2019) e11456.
[19] A. LeFevre, N. Shah, K. Scott, S. Chamberlain, O. Ummer, J.J.H. Bashingwa, A. Chakraborty, A. Godfrey, P. Dutt, D. Mohan, Are stage-based, direct to beneficiary mobile communication programs effective in improving maternal newborn and child health outcomes in India? Results from an individually randomised controlled trial of a national programme, BMJ Global Health, In press (2021).
[20] A. LeFevre, S. Agarwal, S. Chamberlain, K. Scott, A. Godfrey, R. Chandra, A. Singh, N. Shah, D. Dhar, A. Labrique, A. Bhatnagar, D. Mohan, Are stage-based health information messages effective and good value for money in improving maternal newborn and child health outcomes in India? Protocol for an individually randomized controlled trial, Trials 20(1) (2019) 272.
[21] I.I.f.P. Sciences, National Family Health Survey 2015-2016 State Fact Sheet Madhya Pradesh. Mumbai: International Institute for Population Sciences, Government of India, Ministry of Health and Family Welfare; 2016.
[22] A. LeFevre, N. Shah, K. Scott, S. Chamberlain, O. Ummer, J.J. Bashingwa, A. Chakraborty, R. Ved, D. Mohan, Are stage-based mobile health information messages effective in improving maternal newborn and child health outcomes in India? Results from an individually randomized controlled trial Submitted Lancet GH (2021).
[23] B. Dash, D. Mishra, A. Rath, M.J.I.J.o.E. Acharya, Science, Technology, A hybridized K-means clustering approach for high dimensional dataset, 2(2) (2010) 59-66.
[24] C. Ding, X. He, H. Zha, H.D. Simon, Adaptive dimension reduction for clustering high dimensional data, 2002 IEEE International Conference on Data Mining, 2002. Proceedings., IEEE, 2002, pp. 147-154.
[25] S.J.a.p.a. Dasgupta, Experiments with random projection, (2013).
[26] L. Parsons, E. Haque, H.J.A.s.e.n. Liu, Subspace clustering for high dimensional data: a review, 6(1) (2004) 90-105.
[27] C. Ding, T. Li, Adaptive dimension reduction using discriminant analysis and k-means clustering, Proceedings of the 24th international conference on Machine learning, 2007, pp. 521-528.
[28] R. Muthukrishnan, R. Rohini, LASSO: a feature selection technique in predictive modeling for machine learning, 2016 IEEE international conference on advances in computer applications (ICACA), IEEE, 2016, pp. 18-20.
[29] M. Yamada, W. Jitkrittum, L. Sigal, E.P. Xing, M.J.N.c. Sugiyama, High-dimensional feature selection by feature-wise kernelized lasso, 26(1) (2014) 185-207.
[30] J.M. Chambers, Software for data analysis: programming with R, Springer2008.
[31] F. Milano, A Python-based software tool for power system analysis, 2013 IEEE Power & Energy Society General Meeting, IEEE, 2013, pp. 1-5.
[32] N. Dhanachandra, K. Manglem, Y.J.J.P.C.S. Chanu, Image segmentation using K-means clustering algorithm and subtractive clustering algorithm, 54 (2015) 764-771.
[33] A. Likas, N. Vlassis, J.J.J.P.r. Verbeek, The global k-means clustering algorithm, 36(2) (2003) 451-461.
[34] T.M. Kodinariya, P.R.J.I.J. Makwana, Review on determining number of Cluster in K-Means Clustering, 1(6) (2013) 90-95.

For peer review only

[35] C. Genolini, X. Alacoque, M. Sentenac, C.J.J.o.S.S. Arnaud, kml and kml3d: R packages to cluster longitudinal data, 65(4) (2015) 1-34.
[36] M. Liao, Y. Li, F. Kianifard, E. Obi, S.J.B.n. Arcona, Cluster analysis and its application to healthcare claims data: a study of end-stage renal disease patients who initiated hemodialysis, 17(1) (2016) 1-14.
[37] C. Violán, A. Roso-Llorach, Q. Foguet-Boreu, M. Guisado-Clavero, M. Pons-Vigués, E. Pujol-Ribera, J.M.J.B.f.p. Valderas, Multimorbidity patterns with K-means nonhierarchical cluster analysis, 19(1) (2018) 1-11.
[38] R. Das, S. Saleh, I. Nielsen, A. Kaviraj, P. Sharma, K. Dey, S.J.I.J.o.M.I. Saha, Performance analysis of machine learning algorithms and screening formulae for β–thalassemia trait screening of Indian antenatal women, 167 (2022) 104866.
[39] T.M. Santos, B.O. Cata-Preta, C.G. Victora, A.J.J.V. Barros, Finding Children with High Risk of Non-Vaccination in 92 Low-and Middle-Income Countries: A Decision Tree Approach, 9(6) (2021) 646.

Figure 1. Framework for segmentation analysis
Figure 2. Elbow method used to help decide ultimate number of clusters appropriate for the data.
Figure 3. Silhouette analysis for three and four clusters
Figure 4. Distribution of select characteristics with strong signals by Cluster.
Variables which had at least a prevalence of 70% in one or more clusters and differed from another cluster by 50% or more were considered to have a strong signal (*Reported by men interviewed, **Observed by survey enumerators)

For peer review only

Figure 1. Framework for segmentation analysis.

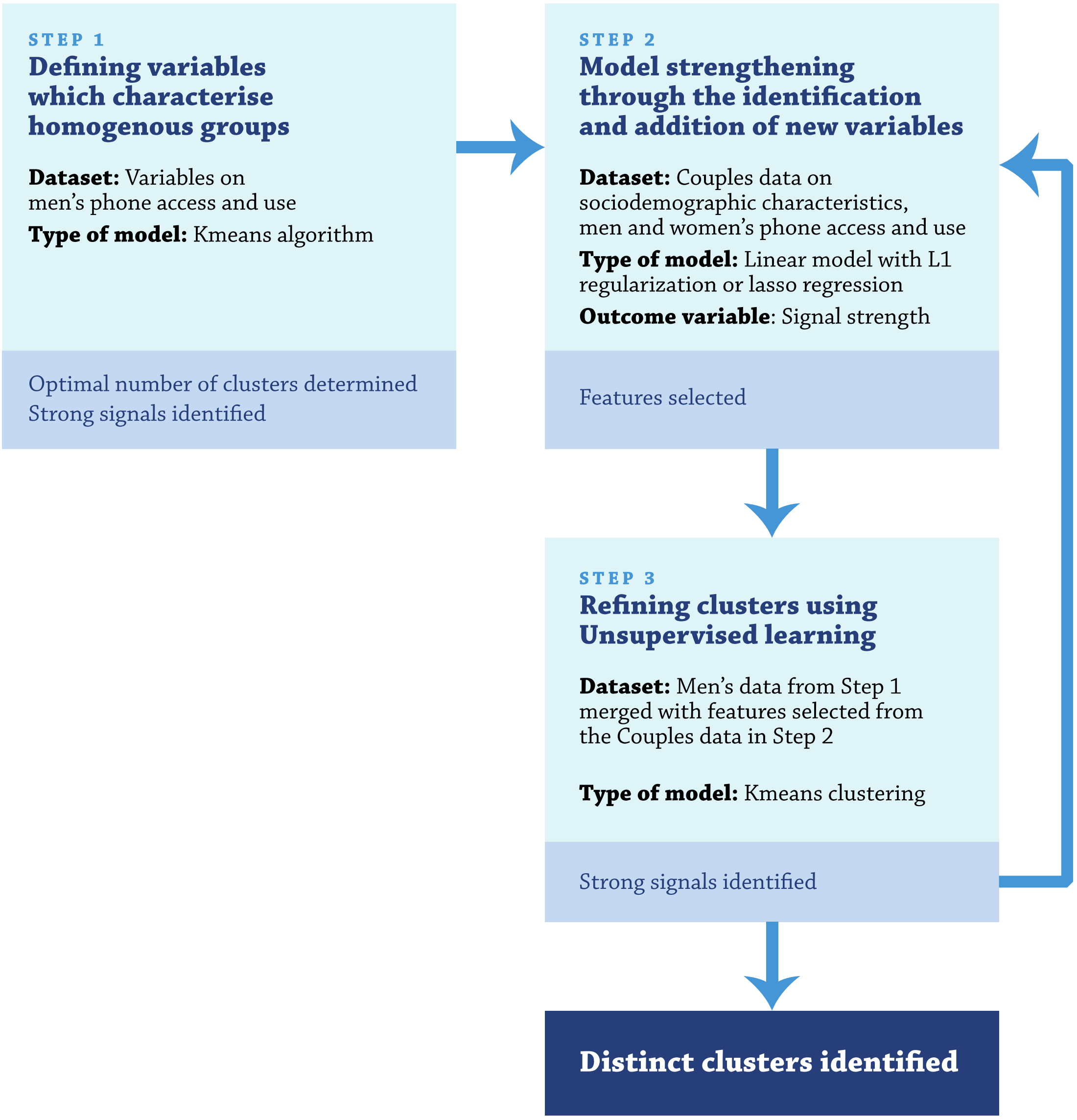

Figure 2. Elbow method used to help decide ultimate number of clusters appropriate for the data.

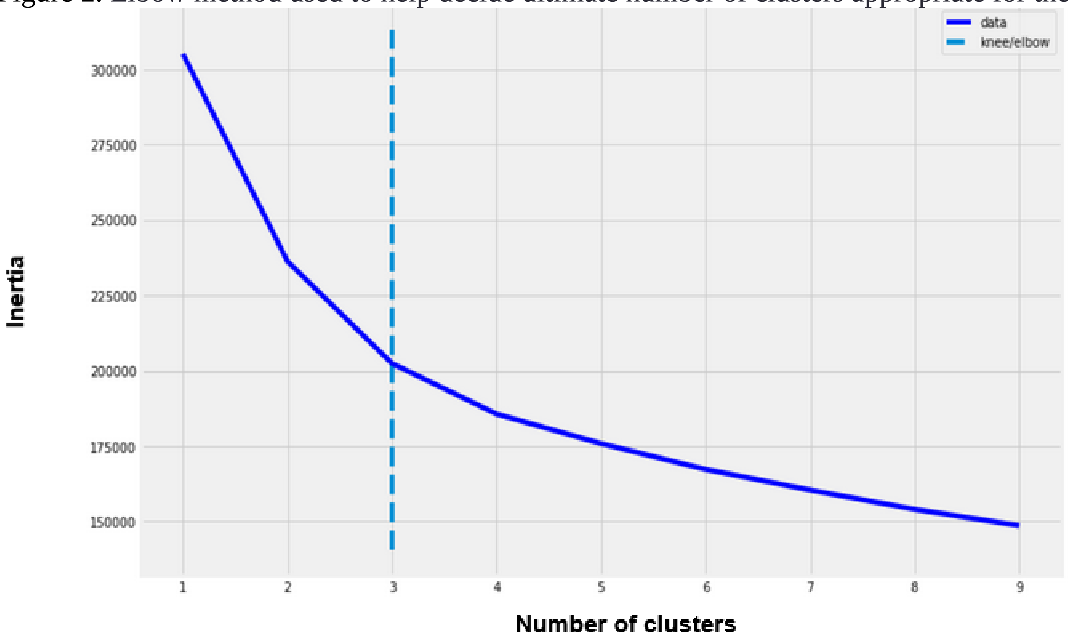

For peer review only

Figure 3. Silhouette analysis for three and four clusters

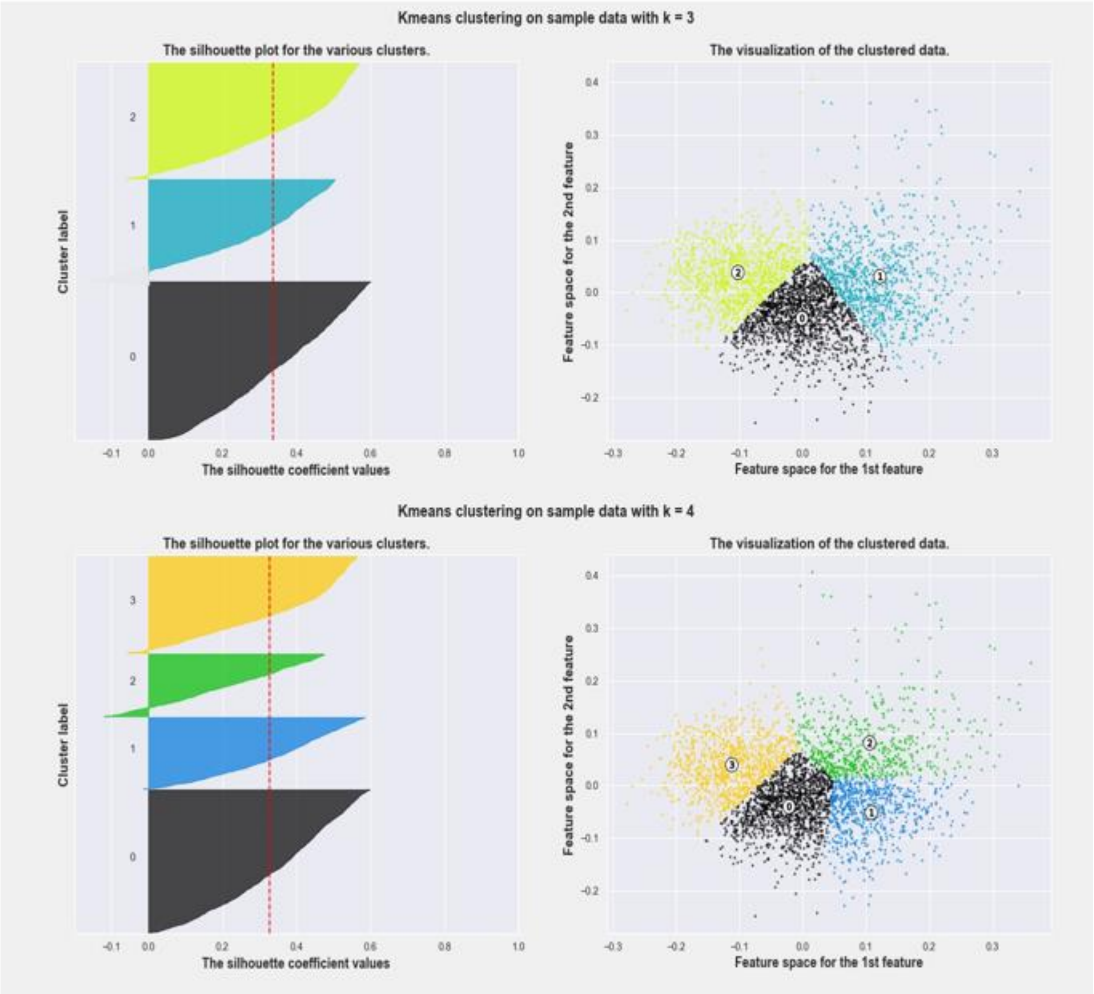

Figure 4. Distribution of select characteristics with strong signals by Cluster. Variables which had at least a prevalence of 70% in one or more clusters and differed from another cluster by 50% or more were considered to have a strong signal.

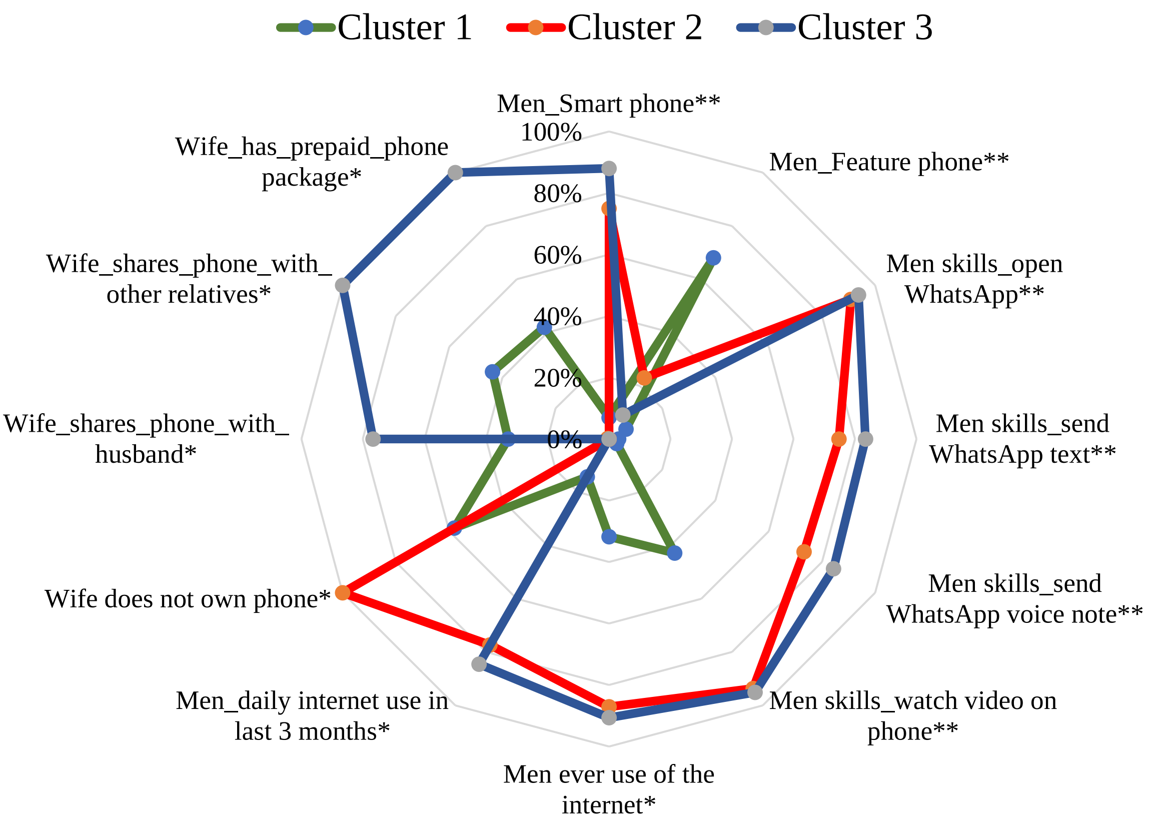

*Reported by men interviewed
**Observed by survey enumerators

For peer review only

Supplementary Table1. Study sample characteristics (variables used as starting point for couple's survey data)

	Women's survey		Men's survey	
**Variables**	N	%	N	%
**Education**				
0-5 years	610	18	586	17
>5 years	2874	82	2898	83
**District**				
Hoshangabad	345	10	345	10
Mandsaur	676	19	676	19
Rajgarh	791	23	791	23
Rewa	1672	48	1672	48
**Ethnicity/Caste**				
General	780	22	698	20
OBC	1690	49	1738	50
Scheduled caste	647	19	690	20
Scheduled tribe	345	10	357	10
**Age at time of enrollment in years**				
18-24	2027	58	564	16
25-34	1391	40	2477	71
35+	66	2	443	13
**Education**				
Never been to school	347	10	100	3
Primary school or less	610	18	586	17
Middle school	1042	30	932	27
High school	1168	34	1322	38
Higher education	317	9	544	16
**MNO**				
Airtel	893	26	791	23
Idea	1572	45	967	28
Jio	229	7	1270	36
Tata	9	0	4	0
vodafone	781	22	427	12
BSNL			24	1
**Frequency of most recent top up**				
More than 3 months	299	9		
Within 1 month	1626	47		

Within 1 week	718	21		
Within 3 months	841	24		
**Who topped up credit**				
Husband	2784	80		
Other	357	10		
self	343	10		
**Who taught respondent how to use phone**				
Husband	794	23		
Other	178	5		
Self	2512	72		
**Permission for wife's phone use**				
Wife takes permission to make call	1133	33		
Wife takes permission before picking up call	1614	46		
Wife takes permission to recharge	838	24		
Women need oversight to use phone	2514	72		
**Type of phone**				
Brick phone	454	13	357	10
Feature phone	2206	63	1234	35
Smart phone	824	24	1838	53
**Use phone to call spouse**	2563	74	2926	84
**Use phone to call ASHAs**	293	8	2478	71
**Use phone for internet**	1	0	1417	41
**Use phone to listen radio**	1	0	1868	54
**Observe phone**				
Phone working	2820	81	3251	93
**Digital Tasks**				
Able to navigate IVR prompts	2995	86	3319	95
Give a missed call	2409	69	2890	83
Store contacts on phone	2845	82	2999	86
Open SMS	1654	47	2966	85
Read SMS	1102	32	2188	63
Overall digital literacy	937	27	1938	56
Open and read SMS	1102	32	2188	63
**Involvement in Decision making**				
About daily household expenditures	713	20	2065	59
About big expenditures	623	18	2243	64

About health during pregnancy	937	27	3081	88
**Employment status**	1398	40	3458	99
**Socio-economic status**				
Poorest	542	16	542	16
Poorer	646	19	646	19
Middle	710	20	710	20
Richer	760	22	760	22
Richest	826	24	826	24
**Phone in the household**				
1	759	22	759	22
2	1437	41	1437	41
>2	1288	37	1288	37
**Parity**				
No child	1406	40	1406	40
One child	1256	36	1256	36
Two and more	822	24	822	24
**Religion**				
Hindu	3297	95	3297	95
Muslim	183	5	183	5
Other	4	0	4	0
**Frequency of phone use in last 3 months**				
Every day	2700	77		
not every day	784	23		
**Age at marriage**				
0-15 years	416	12		
>15 years	3068	88		

Supplementary Table 2. Metrics used for cluster validation (Davies-Bouldin and Calinski-Harabatz criterions have been normalized to [0,1] ,1 indicating a good partition)

Number of clusters	**Within cluster sum of square**	**Silhouette index**	**Ray -Turi index**	**Calinski – Harabatz index**
**2**	64791,07	0,812424	0,873942	0,820123
**3**	62595,37	0,801119	1	0,9563
**4**	60983,52	0,509252	0,853942	0,360082
**5**	59662,45	0,466859	0,529231	0,243941
**6**	58571,27	0,454165	0,482203	0,161834
**7**	57686,73	0,420884	0,427094	0,096974
**8**	56943,46	0,402445	0,249373	0,044445
**9**	56322,05	0,386873	0,268434	0

Table 3a. Men's sample characteristics by cluster based on Men's survey data from four districts of Madhya Pradesh

	**Total** n=3,484		**Cluster 1** n=1,408		**Cluster 2** n=666		**Cluster 3** n=1,410	
	%	n	%	n	%	n	%	n
**Sociodemographic characteristics**								
**Caste**								
General	20	698	15	208	17	112	27	378
OBC	50	1 738	45	637	50	334	54	767
Scheduled tribe	10	357	15	213	11	73	5	71
Scheduled caste	20	690	25	350	22	146	14	194
**Education**								
Never been to school	3	100	7	92	1	6	-	2
Primary school or less	17	586	29	403	13	84	7	99
Middle school	27	932	32	446	28	189	21	297
High school	38	1 322	29	415	42	280	44	627
Higher education	16	544	4	52	16	107	27	385
**Number of phones in the household**								
0-1	22	759	34	476	24	157	9	126
2	41	1 437	45	629	43	284	37	524
3+	37	1 288	22	303	34	225	54	760

**Phone ownership and sharing**								
Own phone and do not share	17	578	16	221	8	50	22	307
Own phone and do share	78	2 730	73	1 031	91	607	77	1 092
Share only	3	93	5	73	1	9	1	11
**Phone type (observed)**								
Brick phone	10	357	22	304	3	17	3	36
Feature phone	35	1 234	68	953	23	151	9	130
Smart phone	53	1 838	7	96	75	498	88	1 244
**Men's phone use**								
Daily phone use (reported)	95	3 327	89	1 260	99	662	100	1 405
**Phone features used (reported)**								
Calls	98	3 422	96	1 350	100	666	100	1 406
SMS	46	1 615	19	263	55	369	70	983
WhatsApp	61	2 109	7	97	95	635	98	1 377
Watch video	80	2 784	52	726	99	659	99	1 399
Share video	58	2 008	6	87	89	591	94	1 330
Make video	35	1 209	9	121	47	316	55	772
Download Apps	47	1 640	2	29	70	468	81	1 143
Music	86	2 984	68	959	97	649	98	1 376
Radio	26	889	14	200	32	210	34	479
Search Google	55	1 925	9	128	82	548	89	1 249
Search YouTube	67	2 327	21	300	98	653	97	1 374
Camera	84	2 921	61	857	99	659	100	1 405
Share photo	59	2 039	7	93	90	602	95	1 344
Mobile money	16	560	0	3	15	103	32	454
Transfer mobile money	13	463	0	1	12	82	27	380
Transfer mobile credit	13	459	0	1	12	83	27	375
**Men's Digital skills (observed)**								
Able to navigate IVR prompts	95	3 319	91	1 280	98	656	98	1 383
Give a missed call	83	2 890	72	1 020	88	588	91	1 282
Store contacts on phone	86	2 999	73	1 031	94	623	95	1 345
Open SMS	85	2 966	71	994	94	624	96	1 348
Read SMS	63	2 188	38	530	73	483	83	1 175
Overall Basic Digital Skill Level	56	1 938	29	415	65	432	77	1 091
**WhatsApp skills (observed)**								
Open WhatsApp	58	2 017	6	91	91	605	94	1 321
Send WhatsApp text	49	1 718	3	44	75	498	83	1 176
Send WhatsApp voice note	49	1 719	3	42	73	488	84	1 189
**Watch video on phone (observed)**	74	2 568	43	603	94	624	95	1 341
**Men report getting images and videos from**								

Internet: YouTube	59	2 062	19	274	83	554	88	1 234
Internet: Google	45	1 569	9	130	64	429	72	1 010
Other relatives	36	1 249	4	63	54	360	59	826
Friends locally	55	1 916	11	153	83	550	86	1 213
Friends other states	25	885	1	21	36	238	44	626
**Computer/ tablet ownership and use**								
Own Computer/ tablet	6	220	1	13	4	28	13	179
Daily computer / tablet use	5	184	0	3	5	30	11	151
Ever use of the internet from any device/ location (reported)	66	2 305	32	447	87	580	91	1 278
Daily internet use in last 3 months (reported)	55	1 906	14	199	77	515	85	1 192
**Wife owns phone**	57	3 484	42	591	-	-	100	1 410
**Wife's phone type**								
Brick phone	10	363	10	134	0	1	16	228
Feature phone	29	1 016	27	375	-	-	45	641
Smart phone	19	647	8	106	-	-	38	541
**Wife shares phone with**								
Husband	44	1 543	33	461	-	-	77	1 082
Children (male or female)	5	180	4	52	-	-	9	128
Parents in law	9	329	6	83	-	-	17	246
Wife's parents	3	107	2	33	-	-	5	74
Other relatives	58	2 028	44	615	0	3	100	1 410
Friend/ neighbour	1	30	1	9	-	-	1	21
**Phone features wife uses (reported)**								
Calls: receive, dial, or speak	100	3 475	100	1 404	100	663	100	1 408
SMS	33	1 146	16	228	28	185	52	733
WhatsApp	35	1 225	11	155	38	255	58	815
Watch shows	54	1 871	26	368	68	450	75	1 053
Music or radio	100	3 484	100	1 408	100	666	100	1 410
Search internet	34	1 192	12	168	36	240	56	784
Camera	74	2 589	55	772	84	559	89	1 258
**Men's perceptions about restrictions (if any) which should be placed on phone use**								
**No restrictions should be placed on adult phone use**	86	2 992	85	1 192	86	571	87	1 229
**Oversight needed for**								
Men	47	1 647	54	767	46	307	41	573
Women	72	2 514	79	1 114	71	476	66	924
Male children	82	2 863	86	1 207	79	523	80	1 133
Female children	92	3 198	93	1 311	91	608	91	1 279
**Men report that their wife needs their permission to pick up**								

**calls from**								
Someone unknown	46	1 614	46	653	51	341	44	620
Family	13	461	17	237	18	122	7	102
Friends/ Neighbours	32	1 121	35	488	41	274	25	359
Health workers	22	757	25	356	29	195	15	206
Business associates	28	990	29	410	35	232	25	348
**Men report women need their permission to make a call to**								
Family	17	600	21	293	24	162	10	145
Friends/ Neighbours	21	735	25	345	28	187	14	203
Health workers	20	692	22	315	29	192	13	185
Business associates	14	484	17	236	16	109	10	139
Unknown to husband	17	608	20	286	20	134	13	188
**Men report women need their permission to send SMS or WhatsApp to**								
Family	2	72	1	12	4	28	2	32
Friends/ Neighbours	3	101	1	12	6	41	3	48
Health workers	2	77	1	9	5	30	3	38
Business associates	2	54	1	11	3	18	2	25
Unknown to husband	3	100	1	13	5	35	4	52
**Man has concerns about wife's phone ownership or use**	1	24	1	10	2	11	0	3
**Reasons for concern (multi-select):**								
Cost of phone	0	3	0	1	0	2	-	-
Cost of using phone	0	9	0	4	0	2	0	3
Reputational risk	0	13	0	5	1	8	-	-
Relationships with other men	0	3	0	2	0	1	-	-
Bad friendships with other women	0	3	0	1	0	2	-	-
Financially defrauded	0	1	-	-	0	1	-	-
**Men would like their wives to use the mobile phone to**								
Transfer money	41	1 439	30	423	42	281	52	735
Buy/ pay for things	37	1 304	26	368	38	256	48	680

Table 3b. Women's sample characteristics by cluster based on women's baseline survey data from four districts of Madhya Pradesh

	Total n=3,484		Cluster 1 n=1,408		Cluster 2 n=666		Cluster 3 n=1,410	
	%	n	%	n	%	n	%	n
**Sociodemographic characteristics**								
**Socioeconomic status**								
Poorest	16	542	26	369	13	88	6	85
Poorer	19	646	27	379	18	117	11	150
Middle	20	710	22	313	25	167	16	230
Richer	22	760	15	214	25	165	27	381
Richest	24	826	9	133	19	129	40	564
**District**								
Hoshangabad	10	345	11	151	11	76	8	118
Mandsaur	19	676	13	181	14	95	28	400
Rajgarh	23	791	21	302	29	191	21	298
Rewa	48	1 672	55	774	46	304	42	594
**Mean age (years)**	72	3 484	25	1 408	23	666	24	1 410
**Ethnicity/Caste**								
General	22	780	17	242	19	129	29	409
OBC	49	1 690	45	628	48	321	53	741
Scheduled caste	19	647	23	322	21	140	13	185
Scheduled tribe	10	345	14	203	11	72	5	70
**Education**								
Never been to school	10	347	16	229	8	50	5	68
Primary school or less	18	610	23	327	17	114	12	169
Middle school	30	1 042	32	451	35	236	25	355
High school	34	1 168	26	363	33	223	41	582
Higher education	9	317	3	38	6	43	17	236
**Phone ownership and sharing**								
Own phone and do not share	51	1 781	43	609	38	256	65	916
Own phone and share	22	772	23	318	22	145	22	309
Share only	26	923	34	475	40	264	13	184
**Phone type (observed)**								
Brick phone	7	248	8	113	8	50	6	85
Feature phone	63	2 206	74	1 040	54	359	57	807
Smart phone	24	824	11	158	28	188	34	478
No phone observed	6	206	7	97	10	69	3	40
**Women's phone characteristics**								
**Phone features (observed)**								
Call	79	2 765	76	1 072	71	470	87	1 223

Speaker	79	2 762	76	1 072	71	470	87	1 220
SMS	79	2 768	76	1 074	71	471	87	1 223
Contacts	79	2 766	76	1 072	71	471	87	1 223
Camera	66	2 302	63	889	60	398	72	1 015
Music/ audio content	69	2 419	66	923	63	419	76	1 077
Internet	49	1 712	42	596	47	312	57	804
Bluetooth	64	2 243	60	842	59	390	72	1 011
Radio/FM	69	2 416	64	907	62	415	78	1 094
**Applications installed on phone (observed)**								
Facebook	25	859	17	237	23	156	33	466
WhatsApp	17	603	8	113	18	117	26	373
Shareit	10	364	4	61	11	71	16	232
**Proportion of phones with zero balance at time of interview**	48	1 666	47	655	50	334	48	677
**Who topped up credit?**								
Husband	80	2 784	79	1 109	81	537	81	1 138
Self	10	357	11	157	12	79	9	121
Other	10	343	10	142	8	50	11	151
**Frequency of most recent top-up**								
Within 1 week	21	718	24	343	19	125	18	250
Within 1 month	47	1 626	46	645	46	309	48	672
Within 3 months	24	841	21	299	23	155	27	387
More than 3 months	9	299	9	121	12	77	7	101
**Total amount of last top up**								
>50	55	1 902	59	831	47	311	54	760
0-50	45	1 582	41	577	53	355	46	650
**Women's phone use**								
**Digital skill (observed)**								
Able to navigate IVR prompts	69	2 409	81	1 142	87	578	90	1 275
Give a missed call	82	2 845	64	895	60	401	79	1 113
Store contacts on phone	47	1 654	73	1 021	83	555	90	1 269
Open SMS	32	1 102	33	471	39	263	65	920
Read SMS	32	1 102	18	255	26	171	48	676
Overall Basic Digital Skill Level	27	937	15	213	21	139	41	585
**Communication**	74	2 563	65	917	68	455	84	1 191
Call with spouse	73	2 542	81	905	80	454	89	1 183
Call with friends, relatives	43	1 485	83	478	87	297	82	710
Call with health workers	32	1 132	99	317	99	196	97	619
SMS with husband	16	545	97	103	99	91	96	351

SMS with friends, relatives	9	330	98	45	100	49	100	236
SMS with health workers	6	213	100	27	100	24	99	162
Dialled a number and listened to pre-recorded message	77	2 700	72	1 010	73	489	85	1 201
**Who taught respondent how to use phone?**								
Spouse	5	178	5	72	5	35	5	71
Self	72	2 512	70	986	71	472	75	1 054
Other	23	794	25	350	24	159	20	285

For peer review only

Supplementary Table 4. Strong signals (variable used for the spide charts are highlighted)

	Cluster 1 (n=1408)	Cluster 2 (n=666)	Cluster 3 (n=1410)
**Men paid for wife's balance**	37	0	90
**Men can perform basic internet search**	7	66	77
**Men report that their wife uses prepaid pack**	42	0	100
**Men report that women need their permission to add credit**	18	0	42
**Men report ever use of internet**	31	87	91
**Observe men watching Video**	42	93	95
**Men can send WhatsApp text**	3	77	85
**Men report use of WhatsApp**	7	91	95
**Men report that their wife's use the phone to**			
Search internet	12	36	55
Watch show	26	66	75
WhatsApp	11	37	57
Men report that they can send photo on WhatsApp	4	88	93
Men report that they can send a WhatsApp voice message	3	73	84
**Men report getting images and videos from**			
Internet: YouTube	19	84	88
Internet: Google	9	64	71
Other relatives	4	55	59
Friends locally	11	83	87
Friends other states	2	36	44
**Men report not using the internet frequently**	86	23	15
**Men have smart phone**	6	75	88
**Men report using the internet frequently**	14	77	85
**Men have feature phone**	68	23	9
**Number of phones in the household**			
3+	19	32	61
0-1	43	39	2
**Men report that their wife own's a phone**	42	0	100
**Men report that their wife does not own a phone**	58	100	0
**Men report their wife shares phone she owns with husband**	32	0	77
**Men observed to open WhatsApp**	6	91	94
**Men's observed digital literacy**	29	64	77
**Men observed to read SMS**	37	72	82
**Features men report using on their phone**			
Share photo	7	90	96
Search YouTube	21	98	98
Search Google	9	82	88
Download Apps	2	70	82
Make video	8	48	55
Share video	6	88	94
Watch video	51	99	99
WhatsApp	7	95	98
SMS	18	55	69
**Observe TikTok App on men's phone**	1	36	48
**Men have internet in their household**	25	54	69
**Men report women having a phone other than Samsung or Jio**	24	0	53

**Men report that women have a feature phone**	26	0	46

Supplementary Figure 1. PCA with 95% of cumulative explained variance on couples' data.

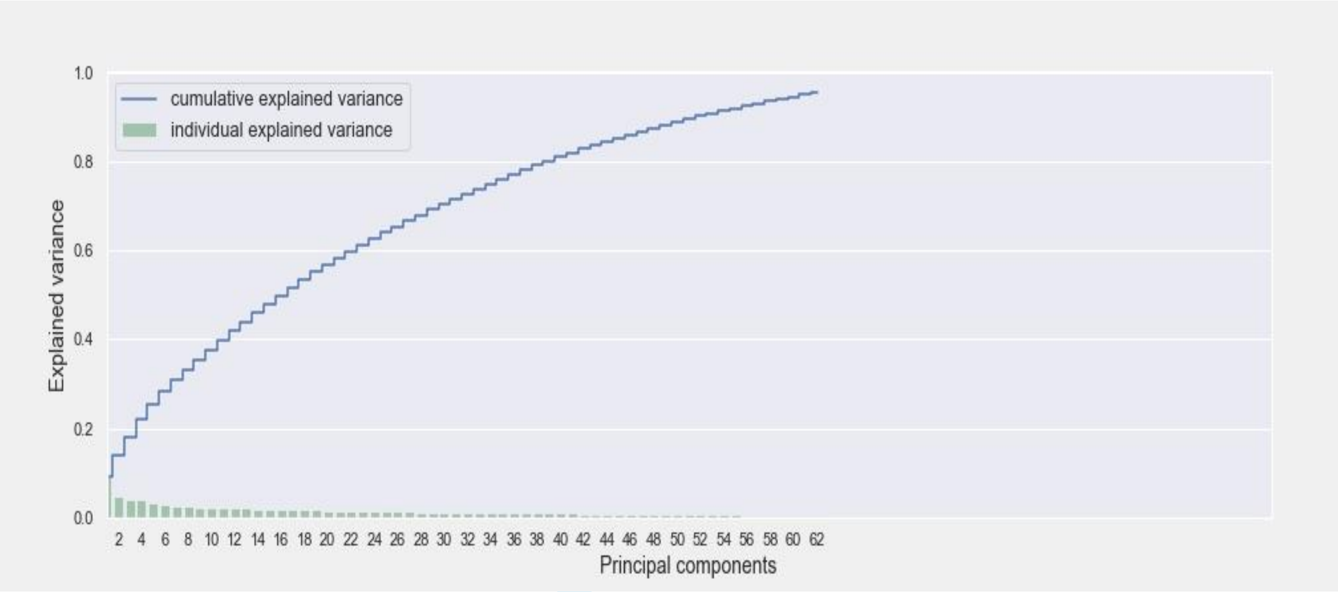

For peer review only

Reporting checklist for quality improvement in health care.

Based on the SQUIRE guidelines.

Instructions to authors

Complete this checklist by entering the page numbers from your manuscript where readers will find each of the items listed below.

Your article may not currently address all the items on the checklist. Please modify your text to include the missing information. If you are certain that an item does not apply, please write "n/a" and provide a short explanation.

Upload your completed checklist as an extra file when you submit to a journal.

In your methods section, say that you used the SQUIREreporting guidelines, and cite them as:

Ogrinc G, Davies L, Goodman D, Batalden P, Davidoff F, Stevens D. SQUIRE 2.0 (Standards for QUality Improvement Reporting Excellence): revised publication guidelines from a detailed consensus process

		Reporting Item	Page Number
**Title**			
	#1	Indicate that the manuscript concerns an initiative to improve healthcare (broadly defined to include the quality, safety,	1

For peer review only

		effectiveness, patientcenteredness, timeliness, cost, efficiency, and equity of healthcare)	
**Abstract**			3
	#02a	Provide adequate information to aid in searching and indexing	3
	#02b	Summarize all key information from various sections of the text using the abstract format of the intended publication or a structured summary such as: background, local problem, methods, interventions, results, conclusions	
**Introduction**			4
Problem description	#3	Nature and significance of the local problem	4
Available knowledge	#4	Summary of what is currently known about the problem, including relevant previous studies	4
Rationale	#5	Informal or formal frameworks, models, concepts, and / or theories used to explain the problem, any reasons or assumptions that were used to develop the intervention(s), and reasons why the intervention(s) was expected to work	4
Specific aims	#6	Purpose of the project and of this report	4
**Methods**			4
Context	#7	Contextual elements considered important at the outset of introducing the intervention(s)	5

For peer review only

Intervention(s)	#08a	Description of the intervention(s) in sufficient detail that others could reproduce it	5
Intervention(s)	#08b	Specifics of the team involved in the work	5
Study of the Intervention(s)	#09a	Approach chosen for assessing the impact of the intervention(s)	6
Study of the Intervention(s)	#09b	Approach used to establish whether the observed outcomes were due to the intervention(s)	6
Measures	#10a	Measures chosen for studying processes and outcomes of the intervention(s), including rationale for choosing them, their operational definitions, and their validity and reliability	6
Measures	#10b	Description of the approach to the ongoing assessment of contextual elements that contributed to the success, failure, efficiency, and cost	7
Measures	#10c	Methods employed for assessing completeness and accuracy of data	7
Analysis	#11a	Qualitative and quantitative methods used to draw inferences from the data	7
Analysis	#11b	Methods for understanding variation within the data, including the effects of time as a variable	7
Ethical considerations	#12	Ethical aspects of implementing and studying the intervention(s) and how they were addressed, including, but	NA

For peer review only

		not limited to, formal ethics review and potential conflict(s) of interest	
**Results**			7
	#13a	Initial steps of the intervention(s) and their evolution over time (e.g., time-line diagram, flow chart, or table), including modifications made to the intervention during the project	7
	#13b	Details of the process measures and outcome	8
	#13c	Contextual elements that interacted with the intervention(s)	8
	#13d	Observed associations between outcomes, interventions, and relevant contextual elements	9
	#13e	Unintended consequences such as unexpected benefits, problems, failures, or costs associated with the intervention(s).	NA
	#13f	Details about missing data	NA
**Discussion**			
Summary	#14a	Key findings, including relevance to the rationale and specific aims	10
Summary	#14b	Particular strengths of the project	10
Interpretation	#15a	Nature of the association between the intervention(s) and the outcomes	10
Interpretation	#15b	Comparison of results with findings from other publications	11

For peer review only

Interpretation	#15c	Impact of the project on people and systems	11
Interpretation	#15d	Reasons for any differences between observed and anticipated outcomes, including the influence of context	11
Interpretation	#15e	Costs and strategic trade-offs, including opportunity costs	11
Limitations	#16a	Limits to the generalizability of the work	11
Limitations	#16b	Factors that might have limited internal validity such as confounding, bias, or imprecision in the design, methods, measurement, or analysis	11
Limitations	#16c	Efforts made to minimize and adjust for limitations	11
Conclusion	#17a	Usefulness of the work	
Conclusion	#17b	Sustainability	11
Conclusion	#17c	Potential for spread to other contexts	12
Conclusion	#17d	Implications for practice and for further study in the field	12
Conclusion	#17e	Suggested next steps	12
**Other information**			12
Funding	#18	Sources of funding that supported this work. Role, if any, of the funding organization in the design, implementation, interpretation, and reporting	2

For peer review only

None The SQUIRE 2.0 checklist is distributed under the terms of the Creative Commons Attribution License CC BY-NC 4.0. This checklist can be completed online using https://www.goodreports.org/, a tool made by the EQUATOR Network in collaboration with Penelope.ai

For peer review only
